# Investigation of the Protection of the C4 Hydroxyl Group in Macrobicyclic Kdo Donors

**DOI:** 10.3390/molecules28010102

**Published:** 2022-12-23

**Authors:** Shogo Hamajima, Naoko Komura, Hide-Nori Tanaka, Akihiro Imamura, Hideharu Ishida, Tsuyoshi Ichiyanagi, Hiromune Ando

**Affiliations:** 1 The United Graduate School of Agricultural Sciences, Gifu University, 1-1 Yanagido, Gifu 501-1193, Japan; 2 Institute for Glyco-Core Research, Gifu University, 1-1 Yanagido, Gifu 501-1193, Japan; 3 Department of Applied Bioorganic Chemistry, Faculty of Applied Biological Sciences, Gifu University, 1-1 Yanagido, Gifu 501-1193, Japan; 4 Department of Life and Environmental Sciences, Faculty of Agriculture, Tottori University, 4-101, Tottori 680-8553, Japan

**Keywords:** glycosidation, stereoselectivity, Kdo, macrobicyclic glycosyl donors, bacterial glycans

## Abstract

Chemical synthesis of 3-deoxy-d-manno-2-octulosonic acid (Kdo)-containing glycans, such as bacterial lipopolysaccharides (LPSs) and capsular polysaccharides (CPSs), is in high demand for the development of vaccines against pathogenic bacteria. We have recently achieved the complete α-stereoselective glycosidation of Kdo using a macrobicyclic donor tethered at the C1 and C5 positions. In this study, to expand the scope of Kdo glycosidation, we sought to protect the 4-OH group, thereby shortening the reaction time and ensuring the conversion of the glycosyl acceptor via its selective removal. The protection of the 4-OH group influenced the reactivity of the Kdo donor, and the triisopropylsilyl (TIPS) group acted as a selectively removable booster. The 4-*O*-TIPS donor allowed the synthesis of the α(2,4)-linked dimeric Kdo sequence, which is widely found in bacterial LPSs.

## 1. Introduction

3-Deoxy-d-manno-2-octulosonic acid (Kdo) constitutes bacterial glycans, such as lipopolysaccharides (LPSs) and capsular polysaccharides (CPSs), which are closely associated with the survival and virulence of pathogenic bacteria [1,2,3]. To understand and harness the functions of Kdo-containing LPSs and CPSs in bacterial infectious diseases, their synthesis is required. The chemical synthesis of structurally defined Kdo-containing glycans and their functionalized probes is highly profitable owing to their poor availability from natural sources. In particular, there has been growing interest in the field of vaccine development. However, the chemical synthesis of Kdo-containing glycans is challenging [4,5]; during the glycosylation of Kdo (referred to as Kdosidation), the stereocontrol is difficult, owing to the absence of a hydroxyl group at the position adjacent to the anomeric center. Previous studies on Kdosidation devised α-selective Kdo donors, such as those protected with bulky groups at the position diagonal to the anomeric position [6,7,8,9,10,11], appended with an auxiliary group at the C3 [12,13,14] and C5 [15] positions, and 2,3-ene derivatives as the precursor of the C3-appended intermediate [16,17,18,19,20]. Recently, DMF-assisted α-selective Kdosidation using a per-acetyl Kdo donor has been demonstrated [21].

**Figure 1:**
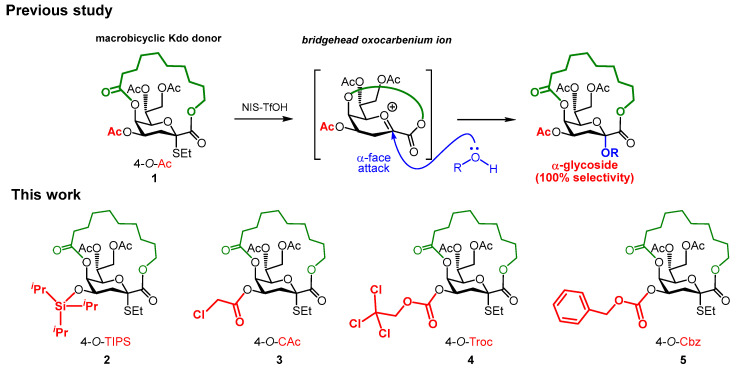
Macrobicyclic Kdo donors developed in previous and present studies.

Full α-selective Kdosidation was achieved using a macrobicyclic Kdo donor with α-configuration, which completely blocked the glycosidation at the β-face by the tether moiety between the C1 and C5 positions (Figure 1) [22]. In principle, this method does not produce any β-isomers and can achieve high yields using various acceptors, thereby facilitating the synthesis of α-Kdo-containing glycans. However, glycosidation using donor **1** required more time compared to glycosidation using a non-bicyclic Kdo donor [22]. In this study, we addressed this issue to expand the applicability of our method and revealed a profound effect of the protecting group at the C4 position.

To tune the reactivity of the macrobicyclic Kdo donor, we used the effect of the hydroxyl protecting group. Based on the enormous results of the reactivities of the glycosyl donors [23,24], we presumed that the protection of the C4 and C7 positions would influence the stability of the oxocarbenium ion of Kdo. Considering the higher efficiency of the protection of the C4 hydroxyl group than that at the C7, we examined the effect of protecting groups at the C4 position. The original 4-*O*-acetyl (Ac) Kdo donor **1** was used as the benchmark, and the protection at the hydroxyl was diversified with selectively removable triisopropylsilyl (TIPS), chloroacetyl (CAc), 2,2,2-trichloroethoxycarbonyl (Troc), and benzyloxycarbonyl (Cbz) groups, which were expected to exert different substituent effects, affording the macrobicylic Kdo donors **2**–**5**. Additionally, the installation of an orthogonal protecting group at this position would be useful for the synthesis of various Kdo-embedded glycans.

## 2. Results

### 2.1. Synthesis of Macrobicyclic Kdo Donors Diversified in the 4-OH Protection

**Scheme 1:**
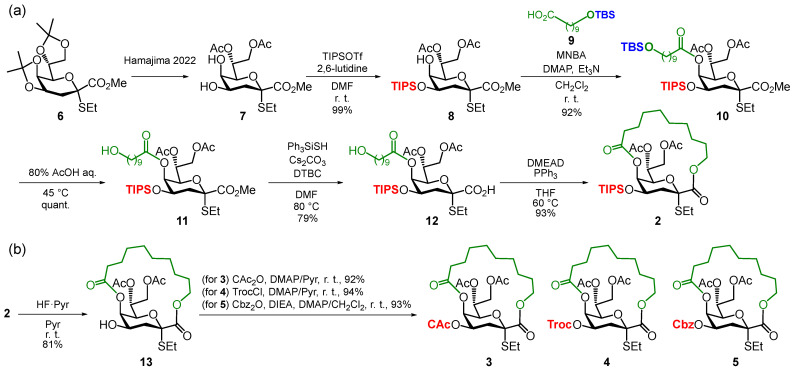
Synthesis of 4-*O*-modified macrobicyclic Kdo donors. (**a**) Synthesis of 4-*O*-TIPS donor **2** [22]; (**b**) Synthesis of 4-*O*-CAc, -Troc, and -Cbz donors **3**, **4,** and **5**. TBS: *tert*-butyldimethylsilyl; MNBA: 2-methyl-6-nitrobenzoic anhydride; DTBC: 2,6,-di-*tert*-butylcresol; DMEAD: di-2-methoxyethyl azodicarboxylate.

To efficiently synthesize the macrobicyclic donors **2**–**5**, the TIPS-protected donor **2** was used as a common intermediate for donors **3**, **4**, and **5** (Scheme 1a). To establish the α-ethylthioglycoside of Kdo, we exploited the reported intramolecular α-selective glycosidation using a dithioketal derivative to produce **6** [17]. Following the procedures reported by our group [22], compound **6** was converted into the 4,5-diol derivative **7**. The TIPS group was then regioselectively introduced at 4-OH, affording **8** in an excellent yield. Next, for macrolactonization, 5-OH was esterified with the tethering unit **9** [22] by Shiina esterification to afford compound **10** in 92% yield. Treatment with 80% aqueous acetic acid followed to selectively remove the TBS protection of the tether moiety in the presence of the TIPS group, yielding **11**. Compound **11** was then converted into the carboxylic acid derivative **12** via selective de-methylation using Ph_3_SiSH [25]. Finally, compound **12** underwent macrolactonization via the Mitsunobu reaction [22,26] to yield the TIPS-protected Kdo donor **2** in 93% yield.

The TIPS group of **2** was selectively removed using a fluoride anion to retrieve a free hydroxyl group at the C4 position (Scheme 1b) and synthesize the other donors. When compound **2** was treated with TBAF and acetic acid in THF, the migration of the ester groups at the O5 and O7 positions occurred randomly to give a complex mixture, whereas the use of HF·pyridine provided the 4-OH derivative **13** in high yield by decreasing the migrated byproduct. Next, the 4-OH, CAc, Troc, and Cbz groups were introduced to afford donors **3**, **4**, and **5**, respectively, in excellent yields.

### 2.2. Examination of α-Glycosidation Using the Macrobicyclic Kdo Donors

The glycosylation of the 6-OH of the glucosyl acceptor **14** was examined to compare the reactivities of the macrobicyclic Kdo donors (Table 1). The protection of the group at the C4 position affected the reactivity of the Kdo donors. Among the tested donors, the TIPS group boosted the reactivity of the donor most significantly. In all cases, equimolar amounts of donor and acceptor were reacted in the presence of *N*-iodosuccinimide (NIS), trifluoromethanesulfonic acid (TfOH), and 3 Å molecular sieves in CH_2_Cl_2_ at −80 °C. The reaction was quenched upon complete consumption of the donor or halt of the reaction, confirmed by TLC analysis. The previously reported result of the glycosidation of 4-*O*-Ac Kdo donor [22] is shown in Entry 1 as the benchmark for this study. Among the tested groups, only the TIPS group was able to shorten the reaction time (by a factor of ~0.62), achieving a 76% yield of the product **16**, comparable to that of Entry 1. However, the introduction of acyl protecting groups slowed down the reaction (Entries 3–5). The result of Entry 3 showed the significant effect of the electron-withdrawing CAc group on the reactivity of the macrobicyclic Kdo donor, in which the reaction time became much longer (96 h) by a factor of ~2.7. In this case, apart from the 2,3-ene derivative, the reaction produced complex side products of the donor, including a 4-OH derivative. Glycoside **17** was obtained in 17% yield, and the unreacted donor was recovered in 35% yield, indicating the significantly reduced reactivity of donor **3**. Likewise, the introduction of carbonate groups, such as Troc and Cbz, retarded the reaction to some extent (Entries 4 and 5). A comparison of Troc and Cbz revealed sensitivity to the electron-withdrawing group. Although the reactions provided similar yields as those of the Kdo glycosides (20% for **18** and 28% for **19**) and 2,3-ene derivatives as major side products (75% for **23** and ~70% for **24**), 4-*O*-Troc donor **4** took much longer (68 h) than the 4-*O*-Cbz donor **5** (42 h). Substitution at the C4 position may affect the electron density of the sulfur atom of the leaving group and the stability of the oxocarbenium ion intermediate, through an unknown mechanism. Therefore, donors with electron-withdrawing groups required a longer period and readily underwent 1,2-elimination rather than glycosidation to produce 2,3-ene side products.

### 2.3. Application of the 4-O-TIPS Kdo Donor 2 to the Synthesis of α(2,4)-Linked Dimeric Kdo

**Scheme 2:**
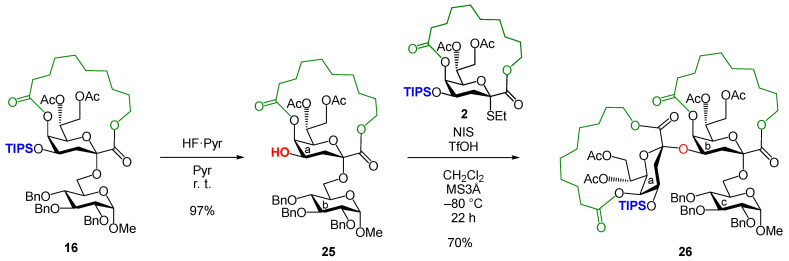
Construction of the α(2,4)−linked dimeric Kdo sequence using the 4−*O*−TIPS Kdo donor **2**.

The synthesis of an α(2,4)-linked dimeric Kdo was attempted, which is widely embedded in the core regions of bacterial LPSs [1], using the 4-*O*-TIPS Kdo donor **2** (Scheme 2). Disaccharide **16** was used as a model of Kdo glycoside and was synthesized following the aforementioned reaction conditions. First, **16** was treated with HF·pyridine in pyridine at room temperature. The reaction successfully removed TIPS without any sequential migration of acyl groups, such as those at 5-, 7-, and 8-OH, affording the disaccharide acceptor **25** in 97% yield. Subsequently, acceptor **25** was glycosylated with Kdo donor **2**. As expected, upon activation with NIS-TfOH in CH_2_Cl_2_ at −80 °C, the reaction proceeded smoothly and ended in 22 h, yielding the dimeric Kdo-containing trisaccharide **26** in 70% yield.

## 3. Materials and Methods

### 3.1. Chemicals

All chemicals were purchased from commercial suppliers and were used as received, unless otherwise noted. The molecular sieves were purchased from FUJIFILM Wako Pure Chemical Corporation (Osaka, Japan) and were pre-dried at 300 °C for 2 h in a muffle furnace followed by drying in a flask at 250 °C for 2 h under vacuum, prior to use. The dry solvents used for the reaction media (CH_2_Cl_2_, toluene, THF, MeCN, DMF, and pyridine) were purchased from Kanto Chemical Co. Inc. (Tokyo, Japan) and were used as received. Other reaction media solvents were dried over molecular sieves and used without purification.

### 3.2. TLC Analysis

TLC analyses were performed on Merck TLC plates (silica gel 60F_254_ on glass plate) (Darmstadt, Germany). Compounds were detected either by exposure to UV light (253.6 nm) or by soaking in a H_2_SO_4_ solution (10% in EtOH) or phosphomolybdic acid solution (20% in EtOH) followed by heating.

### 3.3. Chromatograhic Purification

Flash column chromatography separations were performed by using silica gel (80 mesh and 300 mesh; Fuji silysia Co. (Aichi, Japan)) or Biotage Isolera^TM^ (Uppsala, Sweden) equipped with Biotage SNAP Ultra Silica Cartridges (10 g, 25 g, 50 g, 100 g, and 340 g) (Uppsala, Sweden) or Biotage Sfär Silica HC D Cartridges (10 g, 25 g, 100 g, 200 g, and 350 g) (Uppsala, Sweden). The quantity of silica gel was typically 100 to 200 times the weight of the crude sample. Cytiva Sephadex LH-20 (Tokyo, Japan) was used for size-exclusion chromatography. Solvent systems for chromatography are specified as *v/v* ratios.

### 3.4. Structural Analysis and Acquisition of Physical Data

^1^H and ^13^C NMR spectra were recorded on an Avance III 500 spectrometer (Bruker, Billerica, MA, USA). The chemical shifts in the ^1^H NMR spectra are expressed in ppm (δ), relative to the Me_4_Si signal (0.00 ppm). The chemical shifts in the ^13^C NMR spectra are expressed in ppm (δ), relative to the Me_4_Si (0.00 ppm), CDCl_3_ (77.16 ppm), and adjusted CD_3_OD (49.00 ppm) signals. The data are presented as: chemical shift, multiplicity (s = singlet, br s = broad singlet, d = doublet, t = triplet, q = quartet, quint = quintet, dd = doublet of doublets, td = triplet of doublets, and m = multiplet and/or multiple resonances), integration, coupling constant in hertz (Hz), and position of the corresponding proton. COSY, HMBC, and HMQC methods were used to assign the NMR signals. High-resolution mass spectrometry (ESI-TOF MS) data were obtained using a micrOTOF, Bruker mass spectrometer. Optical rotations were measured with a high-sensitivity polarimeter (SEPA-500, Horiba (Kyoto, Japan)). The copies of ^1^H and ^13^C NMR spectra of the new compounds are provided in the Supplementary Material.

### 3.5. General Procedure for Chemical Reaction

All operations were carried out in a fume hood. All reactions were carried out under a positive pressure of argon unless otherwise noted. Evaporations and concentrations were carried out in vacuo. All heated reactions were carried out in an oil bath. All reactions below 0 °C were carried out in a low temperature reaction tank (PSL-1820, EYELA (Tokyo, Japan)).

### 3.6. Pretreatment for Glycosidation Reaction

A glycosyl donor (1.0–2.0 equiv.) and a glycosyl acceptor (1.0 equiv.) were mixed in a pear-shaped flask, and then residual water was azeotropically removed with dry toluene. The mixture was exposed to high vacuum for 3 h. 3Å molecular sieves (100 mg was used for 1 mL of reaction solvent) were pre-dried at 300 °C for 2 h in a muffle furnace. The pre-dried molecular sieves were added to a two-necked, round-bottomed flask and heated at 250 °C for 2 h in vacuo.

### 3.7. General Procedureof the Glycosidation Reaction

In a pear-shaped flask, a glycosyl donor and a glycosyl acceptor were dissolved in the reaction solvent (50 mM), and the mixture was then transferred via a cannula to a two-necked flask containing the pre-dried molecular sieves. After stirring for 1 h at −80 °C, the promoters were added to the mixture at the same temperature. The progress of the reaction was monitored using TLC analysis and/or mass spectrometry (MALDI-TOF performed with Autoflex, Bruker; matrix: CHCA). The reaction mixture was then quenched, filtered, subjected to an aqueous work-up, and concentrated. The resulting residue was purified by column chromatography. The yields of the isolated coupled products are reported.

### 3.8. Synthesis and Characterisation of the New Compounds

*Ethyl 7,8-di-O-acetyl-3-deoxy-5-O-(10′-hydroxydecanoyl)-2-thio-4-O-triisopropylsilyl-α-d-manno-2-octulopyranosidono-1,10′-lactone* (**2**)

A solution of **12** (1.07 g, 1.55 mmol) and DMEAD (1.45 g, 6.20 mmol) in THF (194 mL) was transferred to a solution of PPh_3_ (1.63 g, 6.20 mmol) in THF (705 mL) at 60 °C over a period of 2 h using a cannula. The cannula was then washed with THF (70.0 mL), and the washings were transferred to the reaction mixture. After stirring for 14 h at 60 °C and while the reaction was monitored using TLC (acetone/*n*-hexane = 1/4), MeOH (1.3 mL, 31.0 mmol) and AcOH (1.8 mL, 31.0 mmol) were added to the reaction mixture. The mixture was concentrated; diluted with CHCl_3_; washed with 2 M HCl aq., water, satd. NaHCO_3_ aq., and brine; dried over Na_2_SO_4_; and concentrated. The residue was purified by gel filtration column chromatography on Sephadex LH-20, using MeOH/CHCl_3_ (1/1) as the eluent, and column chromatography on silica gel, using EtOAc in *n*-hexane (10–14%) as the eluent, to yield **2** (975 mg, 93%) as a colorless syrup: [α]_D_ +127.7° (c 1.0, CHCl_3_); ^1^H NMR (500 MHz, CDCl_3_) δ 5.33 (near d, 1 H, H-5), 5.12–5.09 (m, 1 H, H-7), 4.60 (dd, 1 H, *J*_7,8a_ = 2.4 Hz, *J*_gem_ = 12.3 Hz, H-8a), 4.37–4.33 (m, 3 H, H-4, H-6, CO_2_C*H_2_*CH_2_), 4.29–4.25 (m, 1 H, CO_2_C*H_2_*CH_2_), 4.15 (dd, 1 H, *J*_7,8b_ = 3.6 Hz, H-8b), 2.66–2.46 (m, 2 H, SCH_2_), 2.38–2.26 (m, 3 H, H-3*ax*, OCOC*H_2_*CH_2_), 2.22 (dd, 1 H, *J*_3*eq*,4_ = 4.9 Hz, *J*_gem_ = 13.8 Hz, H-3*eq*), 2.08–2.04 (2 s, 6 H, 2 Ac), 1.76–1.71 (m, 2 H, CO_2_CH_2_C*H_2_*), 1.63–1.58 (m, 2 H, OCOCH_2_C*H_2_*), 1.49–1.26 (m, 10 H, 5 CH_2_), 1.20 (t, 3 H, *J*_vic_ = 7.6 Hz, SCH_2_C*H_3_*), 1.14–1.03 (m, 21 H, 3 *^i^*Pr); ^13^C NMR (125 MHz, CDCl_3_) δ 172.6, 170.5, 169.8, 168.7, 85.3, 68.8, 67.9, 67.2, 66.5, 66.0, 62.2, 36.5, 34.2, 29.0, 28.5, 28.4, 28.0, 27.9, 26.7, 25.0, 22.5, 20.8, 18.0, 17.9, 14.0, 12.2; HRMS (ESI) *m*/*z*: [M+Na]^+^ Calcd for C_33_H_58_O_10_SSiNa 697.3412; Found 697.3412.

*Ethyl 7,8-di-O-acetyl-4-O-chloroacetyl-3-deoxy-5-O-(10′-hydroxydecanoyl)-2-thio-α-d-manno-2-octulopyranosidono-1,10′-lactone* (**3**)

To a solution of **13** (60.3 mg, 116 µmol) in pyridine (1.2 mL), CAc_2_O (59.5 mg, 348 µmol) and DMAP (1.4 mg, 12 µmol) were added at 0 °C. After stirring for 30 min at room temperature and while the reaction was monitored by TLC (EtOAc/*n*-hexane = 1/2), MeOH was added at 0 °C. The mixture was co-evaporated with toluene; diluted with CHCl_3_; and washed with 2 M HCl aq., water, 5% NaHCO_3_ aq., and brine. The organic layer was dried over Na_2_SO_4_ and concentrated. The residue was purified by column chromatography on silica gel, using EtOAc in *n*-hexane (20%) as the eluent, to afford **3** (63.6 mg, 92%) as a white amorphous mass: [α]_D_ +162.8° (c 0.9, CHCl_3_); ^1^H NMR (500 MHz, CDCl_3_) δ 5.46–5.42 (m, 1 H, H-4), 5.37–5.36 (m, 1 H, H-5), 5.22–5.19 (m, 1 H, H-7), 4.63 (dd, 1 H, *J*_7,8a_ = 2.4 Hz, *J*_gem_ = 12.4 Hz, H-8a), 4.49–4.42 (m, 2 H, H-6, CO_2_C*H_2_*CH_2_), 4.24–4.20 (m, 1 H, CO_2_C*H_2_*CH_2_), 4.13 (dd, 1 H, *J*_7,8b_ = 3.6 Hz, H-8b), 4.01 (d, 1 H, *J*_gem_ = 15.4 Hz, ClH_2_C), 3.97 (d, 1 H, ClH_2_C), 2.71–2.50 (m, 2 H, SCH_2_), 2.43–2.38 (m, 2 H, H-3*ax*, OCOC*H_2_*CH_2_), 2.34–2.27 (m, 2 H, H-3*eq*, OCOC*H_2_*CH_2_), 2.09–2.01 (2 s, 6 H, 2 Ac), 1.78–1.55 (m, 4 H, CO_2_CH_2_C*H_2_*, OCOCH_2_C*H_2_*), 1.46–1.25 (m, 10 H, 5 CH_2_), 1.21 (t, 3 H, *J*_vic_ = 7.6 Hz, SCH_2_C*H_3_*); ^13^C NMR (125 MHz, CDCl_3_) δ 173.3, 170.4, 169.6, 167.9, 166.3, 84.6, 69.3, 68.2, 67.7, 66.3, 64.2, 61.8, 40.6, 33.9, 32.1, 29.0, 28.5, 28.3, 28.0, 27.9, 26.8, 25.3, 22.5, 20.8, 20.7, 13.8; HRMS (ESI) *m/z*: [M+Na]^+^ Calcd for C_26_H_39_ClO_11_SNa 617.1794; Found 617.1790.

*Ethyl 7,8-di-O-acetyl-4-O-(2,2,2-trichloroethoxycarbonyl)-3-deoxy-5-O-(10′-hydroxydecanoyl)-2-thio-α-d-manno-2-octulopyranosidono-1,10′-lactone* (**4**)

To a solution of **13** (60.4 mg, 117 µmol) in pyridine (1.2 mL), TrocCl (47.1 µL, 351 µmol) and DMAP (1.4 mg, 12 µmol) were added at 0 °C. After stirring for 30 min at room temperature, TrocCl (47.1 µL, 351 µmol) was added, and the mixture was stirred for a total of 1.5 h as the reaction was monitored by TLC (EtOAc/*n*-hexane = 1/2). MeOH was added to the reaction mixture at 0 °C. The mixture was co-evaporated with toluene; diluted with CHCl_3_; and washed with 2 M HCl aq., water, satd. NaHCO_3_ aq., and brine. The organic layer was dried over Na_2_SO_4_ and concentrated. The residue was purified by column chromatography on silica gel, using EtOAc in *n*-hexane (20%) as the eluent, to yield **4** (76.1 mg, 94%) as a white amorphous mass: [α]_D_ +145.5° (c 1.0, CHCl_3_); ^1^H NMR (500 MHz, CDCl_3_) δ 5.52 (near d, 1 H, H-5), 5.25–5.21 (m, 1 H, H-4), 5.20–5.17 (m, 1 H, H-7), 4.84 (d, 1 H, *J*_gem_ = 11.8 Hz, CCl_3_CH_2_), 4.68 (d, 1 H, CCl_3_CH_2_), 4.64 (dd, 1 H, *J*_7,8a_ = 2.4 Hz, *J*_gem_ = 12.4 Hz, H-8a), 4.47 (dd, 1 H, *J*_5,6_ = 1.1 Hz, *J*_6,7_ = 9.7 Hz, H-6), 4.45–4.21 (m, 2 H, CO_2_C*H_2_*CH_2_), 4.13 (dd, 1 H, *J*_7,8b_ = 3.5 Hz, H-8b), 2.72–2.50 (m, 2 H, SCH_2_), 2.44 (near t, 1 H, H-3*ax*), 2.40–2.30 (m, 3 H, H-3*eq*, OCOC*H_2_*CH_2_), 2.09–2.02 (2 s, 6 H, 2 Ac), 1.78–1.57 (m, 4 H, CO_2_CH_2_C*H_2_*, OCOCH_2_C*H_2_*), 1.50–1.25 (m, 10 H, 5 CH_2_), 1.21 (t, 3 H, *J*_vic_ = 7.6 Hz, SCH_2_C*H_3_*); ^13^C NMR (125 MHz, CDCl_3_) δ 173.0, 170.4, 169.6, 167.9, 152.7, 94.1, 84.6, 72.4, 68.2, 67.7, 66.3, 63.7, 61.8, 33.8, 32.3, 29.0, 28.4, 28.3, 28.1, 28.0, 26.7, 25.2, 22.5, 20.8, 20.7, 13.8; HRMS (ESI) *m/z*: [M+Na]^+^ Calcd for C_27_H_39_Cl_3_O_12_SNa 715.1120; Found 715.1123.

*Ethyl 7,8-di-O-acetyl-4-O-benzyloxycarbonyl-3-deoxy-5-O-(10′-hydroxydecanoyl)-2-thio-α-d-manno-2-octulopyranosidono-1,10′-lactone* (**5**)

To a solution of **13** (31.0 mg, 59.8 µmol) in CH_2_Cl_2_ (1.2 mL), DIEA (30.4 µL, 179 µmol), DMAP (7.3 mg, 60 µmol), and CbzCl (25.2 µL, 179 µmol) were added at 0 °C. To complete the reaction, DIEA (30.4 µL, 179 µmol), DMAP (7.3 mg, 60 µmol), and CbzCl (25.2 µL, 179 µmol) were added after 7, 10, and 11 h. The mixture was stirred for a total of 12.5 h as the reaction was monitored by TLC (EtOAc/*n*-hexane = 1/2), and MeOH was added to the reaction mixture at 0 °C. The mixture was co-evaporated with toluene; diluted with CHCl_3_; and washed with 2 M HCl aq., water, satd. NaHCO_3_ aq., and brine. The organic layer was dried over Na_2_SO_4_ and concentrated. The residue was purified by column chromatography on silica gel, using EtOAc in *n*-hexane (30%) as the eluent, to afford **5** (36.4 mg, 93%) as a white amorphous mass: [α]_D_ +173.8° (c 1.8, CHCl_3_); ^1^H NMR (500 MHz, CDCl_3_) δ 7.39–7.32 (m, 5 H, Ph), 5.51 (near d, 1 H, H-5), 5.22–5.15 (m, 3 H, H-4, H-7, PhC*H_2_*), 5.13 (d, 1 H, *J*_gem_ = 12.1 Hz, PhC*H_2_*), 4.64 (d, 1 H, *J*_7,8a_ = 2.4 Hz, *J*_gem_ = 12.3 Hz, H-8a), 4.46 (dd, 1 H, *J*_5,6_ = 0.9 Hz, *J*_6,7_ = 9.8 Hz, H-6), 4.43–4.20 (m, 2 H, CO_2_C*H_2_*CH_2_), 4.13 (dd, 1 H, *J*_7,8b_ = 3.5 Hz, H-8b), 2.70–2.49 (m, 2 H, SCH_2_), 2.41–2.30 (m, 4 H, H-3*ax*, H-3*eq*, OCOC*H_2_*CH_2_), 2.08–2.03 (2 s, 6 H, 2 Ac), 1.77–1.54 (m, 4 H, CO_2_CH_2_C*H_2_*, OCOCH_2_C*H_2_*), 1.51–1.23 (m, 10 H, 5 CH_2_), 1.20 (t, 3 H, *J*_vic_ = 7.6 Hz, SCH_2_C*H_3_*); ^13^C NMR (125 MHz, CDCl_3_) δ 172.9, 170.4, 169.6, 168.1, 153.9, 135.0, 128.6, 128.3, 84.7, 71.3, 70.0, 68.3, 67.7, 66.2, 64.1, 61.8, 33.9, 32.3, 29.0, 28.4, 28.0, 26.7, 25.1, 22.5, 20.8, 20.7, 13.8; HRMS (ESI) *m/z*: [M+Na]^+^ Calcd for C_32_H_44_O_12_SNa 675.2446; Found 675.2442.

*Methyl (ethyl 7,8-di-O-acetyl-3-deoxy-2-thio-4-O-triisopropylsilyl-α-d-manno-2-octulopyranosid)onate* (**8**)

To a solution of **7** (1.01 g, 2.66 mmol) in DMF (13.3 mL), 2,6-lutidine (930 µL, 7.98 mmol) and TIPSOTf (1.1 mL, 4.0 mmol) were added at 0 °C. After stirring for 16 h at room temperature and while the reaction was monitored by TLC (MeOH/CHCl_3_ = 1/10), the reaction was quenched with MeOH at 0 °C. The mixture was co-evaporated with toluene; diluted with CHCl_3_; and washed with 2 M HCl aq., water, satd. NaHCO_3_ aq., and brine. The organic layer was dried over Na_2_SO_4_ and concentrated. The residue was purified by column chromatography on silica gel, using EtOAc in *n*-hexane (20–25%) as the eluent, to afford **8** (1.41 g, 99%) as a colorless syrup: [α]_D_ +138.4° (c 0.9, CHCl_3_); ^1^H NMR (500 MHz, CDCl_3_) δ 5.45–5.42 (m, 1 H, H-7), 4.75 (dd, 1 H, *J*_7,8a_ = 2.4 Hz, *J*_gem_ = 12.3 Hz, H-8a), 4.30–4.26 (m, 1 H, H-4), 4.25–4.21 (m, 2 H, H-6, H-8b), 3.79 (s, 4 H, H-5, CO_2_CH_3_), 2.62–2.47 (m, 2 H, SCH_2_), 2.40 (s, 1 H, OH), 2.23 (dd, 1 H, *J*_3*ax*,4_ = 11.3 Hz, *J*_gem_ = 13.9 Hz, H-3*ax*), 2.14 (dd, 1 H, *J*_3*eq*,4_ = 5.0 Hz, H-3*eq*), 2.07–2.06 (2 s, 6 H, 2 Ac), 1.19 (t, 3 H, *J*_vic_ = 7.6 Hz, SCH_2_C*H_3_*), 1.13–1.05 (m, 21 H, 3 *^i^*Pr); ^13^C NMR (125 MHz, CDCl_3_) δ 170.1, 169.8, 169.1, 85.1, 70.0, 69.8, 67.5, 66.7, 62.4, 52.7, 34.7, 22.4, 20.9, 20.8, 18.0, 14.1, 12.2; HRMS (ESI) *m/z*: [M+Na]^+^ Calcd for C_24_H_44_O_9_SSiNa 559.2368; Found 559.2365.

*Methyl {ethyl 7,8-di-O-acetyl-5-O-[10-(tert-butyldimethylsiloxy)decanoyl]-3-deoxy-2-thio-4-O-triisopropylsilyl-α-d-manno-2-octulopyranosid}onate* (**10**)

To a solution of **8** (80.6 mg, 150 µmol) and **9** (136 mg, 450 µmol) in CH_2_Cl_2_ (5.0 mL), Et_3_N (62.7 µL, 450 µmol), DMAP (55.0 mg, 450 µmol), and MNBA (136 mg, 450 µmol) were added at 0 °C. The reaction mixture was stirred for 5 h at room temperature while the reaction was monitored by TLC (EtOAc/*n*-hexane = 1/4). The mixture was diluted with CHCl_3_; washed with 2 M HCl aq., water, satd. NaHCO_3_ aq., and brine; dried over Na_2_SO_4_; and concentrated. The residue was purified by column chromatography on silica gel, using EtOAc/*n*-hexane (8–10%) as the eluent, and gel filtration column chromatography on Sephadex LH-20, using MeOH/CHCl_3_ (1/1) as the eluent, to yield **10** (113 mg, 92%) as a colorless syrup: [α]_D_ +84.6° (c 0.8, CHCl_3_); ^1^H NMR (500 MHz, CDCl_3_) δ 5.34 (near d, 1 H, H-5), 5.14–5.11 (m, 1 H, H-7), 4.58 (dd, 1 H, *J*_7,8a_ = 2.4 Hz, *J*_gem_ = 12.3 Hz, H-8a), 4.39–4.35 (m, 2 H, H-4, H-6), 4.14 (dd, 1 H, *J*_7,8b_ = 3.7 Hz, H-8b), 3.82 (s, 3 H, CO_2_CH_3_), 3.59 (t, 2 H, *J*_vic_ = 6.7 Hz, TBSOC*H_2_*), 2.61–2.44 (m, 2 H, SCH_2_), 2.35–2.26 (m, 2 H, COC*H_2_*CH_2_), 2.24–2.16 (m, 2 H, H-3*ax*, H-3*eq*), 2.07–2.03 (2 s, 6 H, 2 Ac), 1.62–1.47 (m, 4 H, COCH_2_C*H_2_*, TBSOCH_2_C*H_2_*), 1.27 (br s, 10 H, 5 CH_2_), 1.19 (t, 3 H, *J*_vic_ = 7.6 Hz, SCH_2_C*H_3_*), 1.10–1.03 (m, 21 H, 3 *^i^*Pr), 0.89 (s, 9 H, *^t^*Bu), 0.04 (s, 6 H, Si(CH_3_)_2_); ^13^C NMR (125 MHz, CDCl_3_) δ 172.8, 170.5, 169.7, 169.0, 85.3, 68.9, 67.8, 66.8, 66.4, 63.3, 62.2, 52.8, 35.9, 34.1, 32.9, 29.5, 29.4, 29.3, 29.2, 26.0, 25.8, 24.7, 22.5, 20.8, 18.4, 18.0, 14.0, 12.2, -5.2; HRMS (ESI) *m/z*: [M+Na]^+^ Calcd for C_40_H_76_O_11_SSi_2_Na 843.4539; Found 843.4540.

*Methyl [ethyl 7,8-di-O-acetyl-3-deoxy-5-O-(10-hydroxydecanoyl)-2-thio-4-O-triisopropylsilyl-α-d-manno-2-octulopyranosid]onate* (**11**)

A solution of **10** (1.65 g, 2.01 mmol) in 80% AcOH aq. (20.1 mL) was stirred for 30 min at 45 °C while the reaction was monitored by TLC (EtOAc/*n*-hexane = 1/2). The reaction mixture was neutralized using satd. NaHCO_3_ aq. at 0 °C. The mixture was diluted with CHCl_3_; washed with satd. NaHCO_3_ aq. and brine; dried over Na_2_SO_4_; and concentrated. The residue was purified by column chromatography on silica gel, using EtOAc/*n*-hexane (40%) as the eluent, to afford **11** (1.51 g, quant.) as a colorless syrup: [α]_D_ +92.8° (c 1.3, CHCl_3_); ^1^H NMR (500 MHz, CDCl_3_) δ 5.34 (near d, 1 H, H-5), 5.14–5.11 (m, 1 H, H-7), 4.59 (dd, 1 H, *J*_7,8a_ = 2.5 Hz, *J*_gem_ = 12.3 Hz, H-8a), 4.39–4.35 (m, 2 H, H-4, H-6), 4.14 (dd, 1 H, *J*_7,8b_ = 3.6 Hz, H-8b), 3.82 (s, 3 H, CO_2_CH_3_), 3.63 (t, 2 H, *J*_vic_ = 6.7 Hz, HOC*H_2_*), 2.61–2.44 (m, 2 H, SCH_2_), 2.35–2.26 (m, 2 H, COC*H_2_*CH_2_), 2.24–2.16 (m, 2 H, H-3*ax*, H-3*eq*), 2.07–2.03 (2 s, 6 H, 2 Ac), 1.61–1.50 (m, 4 H, COCH_2_C*H_2_*, HOCH_2_C*H_2_*), 1.39–1.26 (m, 11 H, 5 CH_2_), 1.19 (t, 3 H, *J*_vic_ = 7.6 Hz, SCH_2_C*H_3_*), 1.14–1.03 (m, 21 H, 3 *^i^*Pr); ^13^C NMR (125 MHz, CDCl_3_) δ 172.8, 170.5, 169.7, 169.0, 85.3, 68.9, 67.9, 66.8, 66.4, 63.1, 62.2, 52.8, 35.9, 34.1, 32.8, 29.35, 29.34, 29.2, 29.1, 25.7, 24.7, 22.5, 20.8, 18.0, 14.0, 12.2; HRMS (ESI) *m/z*: [M+Na]^+^ Calcd for C_34_H_62_O_11_SSiNa 729.3674; Found 729.3673.

*Ethyl 7,8-di-O-acetyl-3-deoxy-5-O-(10-hydroxydecanoyl)-2-thio-4-triisopropylsilyl-α-d-manno-2-octulopyranosylonic acid* (**12**)

To a solution of **11** (1.53 g, 2.17 mmol) in DMF (27.1 mL), 2,6-di-*tert*-butyl-*p*-cresol (95.6 mg, 434 µmol), Ph_3_SiSH (1.90 g, 6.51 mmol), and Cs_2_CO_3_ (1.91 g, 5.86 mmol) were added at room temperature. After stirring for 4.5 h at 80 °C and while the reaction was monitored by TLC (MeOH/CHCl_3_ = 1/5), the solution was co-evaporated with toluene. The resulting mixture was diluted with EtOAc; washed with 2 M HCl aq., water, and brine; dried over Na_2_SO_4_; and concentrated. The residue was purified by column chromatography on silica gel, using MeOH in CHCl_3_ (2.5–10%) as the eluent, to afford **12** (1.19 g, 79%) as a white powder: [α]_D_ +64.8° (c 1.1, MeOH); ^1^H NMR (500 MHz, CD_3_OD) δ 5.33 (near d, 1 H, H-5), 5.07–5.05 (m, 1 H, H-7), 4.58 (dd, 1 H, *J*_7,8a_ = 2.1 Hz, *J*_gem_ = 12.3 Hz, H-8a), 4.43–4.39 (m, 2 H, H-4, H-6), 4.21 (dd, 1 H, *J*_7,8b_ = 3.8 Hz, H-8b), 3.54 (t, 2 H, *J*_vic_ = 6.7 Hz, HOC*H_2_*), 2.65–2.50 (m, 2 H, SCH_2_), 2.30–2.26 (m, 2 H, COC*H_2_*CH_2_, H-3*eq*), 2.04–1.99 (2 s, 6 H, 2 Ac), 1.59–1.50 (m, 4 H, COCH_2_C*H_2_*, HOCH_2_C*H_2_*), 1.31–1.29 (m, 10 H, 5 CH_2_), 1.21 (t, 3 H, *J*_vic_ = 7.5 Hz, SCH_2_C*H_3_*), 1.08 (br s, 21 H, 3 *^i^*Pr); ^13^C NMR (125 MHz, CD_3_OD) δ 174.3, 172.3, 171.5, 70.3, 69.5, 68.8, 68.3, 63.3, 63.0, 38.1, 35.1, 33.7, 30.5, 30.4, 30.2, 26.9, 25.9, 23.8, 20.9, 20.7, 18.51, 18.49, 14.4, 13.5; HRMS (ESI) *m/z*: [M−H]^−^ Calcd for C_33_H_59_O_11_SSi 691.3553; Found 691.3551.

*Ethyl 7,8-di-O-acetyl-3-deoxy-5-O-(10′-hydroxydecanoyl)-2-thio-α-d-manno-2-octulopyranosidono-1,10′-lactone* (**13**)

To a solution of **2** (32.9 mg, 48.8 µmol) in pyridine (1.0 mL), HF pyridine (100 µL) was added at 0 °C. After stirring for 19 h at room temperature, HF pyridine (100 µL) was added, and the mixture was stirred for a total of 42 h while the reaction was monitored by TLC (EtOAc/*n*-hexane = 1/2). Satd. NaHCO_3_ aq. was added to the reaction mixture at 0 °C. The mixture was diluted with CHCl_3_; washed with satd. NaHCO_3_ aq. and brine; dried over Na_2_SO_4_; and concentrated. The residue was purified by column chromatography on silica gel, using EtOAc in *n*-hexane (30%) as the eluent, to yield **13** (20.5 mg, 81%) as a white amorphous mass: [α]_D_ +144.5° (c 1.2, CHCl_3_); ^1^H NMR (500 MHz, CDCl_3_) δ 5.24–5.21 (m, 2 H, H-5, H-7), 4.63 (dd, 1 H, *J*_7,8a_ = 2.4 Hz, *J*_gem_ = 12.3 Hz, H-8a), 4.45–4.38 (m, 2 H, CO_2_C*H_2_*CH_2_, H-6), 4.35–4.30 (m, 1 H, H-4), 4.24–4.20 (m, 1 H, CO_2_C*H_2_*CH_2_), 4.16 (dd, 1 H, *J*_7,8b_ = 3.9 Hz, H-8b), 2.67–2.48 (m, 2 H, SCH_2_), 2.47–2.32 (m, 2 H, OCOC*H_2_*CH_2_), 2.29 (dd, 1 H, *J*_3*eq*,4_ = 4.6 Hz, *J*_gem_ = 13.9 Hz, H-3*eq*), 2.24 (d, 1 H, *J*_4,OH_ = 3.4 Hz, OH), 2.16 (dd, 1 H, *J*_3*ax*,4_ = 12.1 Hz, H-3*ax*), 2.08–2.04 (2 s, 6 H, 2 Ac), 1.77–1.57 (m, 4 H, CO_2_CH_2_C*H_2_*, OCOCH_2_C*H_2_*), 1.51–1.25 (m, 10 H, 5 CH_2_), 1.20 (t, 3 H, *J*_vic_ = 7.6 Hz, SCH_2_C*H_3_*); ^13^C NMR (125 MHz, CDCl_3_) δ 174.9, 170.5, 169.6, 168.4, 85.1, 68.3, 67.8, 66.3, 66.1, 62.1, 35.1, 34.1, 29.0, 28.5, 28.3, 28.1, 28.0, 26.8, 25.3, 22.6, 20.8, 20.7, 13.9; HRMS (ESI) *m/z*: [M+Na]^+^ Calcd for C_24_H_38_O_10_SNa 541.2078; Found 541.2077.

*Methyl 6-O-[7,8-di-O-acetyl-3-deoxy-5-O-(10′-hydroxydecanoyl)-4-O-triisopropylsilyl-α-d-manno-2-octulopyranosylono-1,10′-lactone]-2,3,4-tri-O-benzyl-α-d-glucopyranoside* (**16**) *and 7,8-di-O-acetyl-2,6-anhydro-3-deoxy-5-O-(10′-hydroxydecanoyl)-4-O-triisopropylsilyl-d-manno-2-octenosono-1,10′-lactone* (**21**)

In this step, 3 Å molecular sieves (110 mg) and NIS (19.3 mg, 85.7 µmol) were added to a solution of **2** (38.5 mg, 57.1 µmol) and **14** (26.5 mg, 57.1 µmol) in CH_2_Cl_2_ (1.1 mL) at room temperature. After stirring for 1 h at −80 °C, TfOH (1.0 µL, 11 µmol) was added to the mixture at −80 °C. The reaction mixture was stirred for 22 h at −80 °C while the reaction was monitored by TLC (acetone/toluene = 1/14, developed twice). The reaction was quenched with Et_3_N, and the mixture was filtered through a pad of Celite. The pad was then washed with CHCl_3_. The combined filtrate and washings were washed with satd. Na_2_S_2_O_3_ aq. and brine, dried over Na_2_SO_4_, and concentrated. The resulting residue was purified by gel filtration column chromatography on Sephadex LH-20, using MeOH/CHCl_3_ (1/1) as the eluent, and column chromatography on silica gel, using EtOAc in *n*-hexane (12%) as the eluent, to afford **16** (46.9 mg, 76%) as a colorless syrup and **21** (6.9 mg, 20%) as a colorless syrup. Compound **16**: [α]_D_ +36.0° (c 4.9, CHCl_3_); ^1^H NMR (500 MHz, CDCl_3_) δ 7.36–7.25 (m, 15 H, 3 Ph), 5.33 (near d, 1 H, H-5*^a^*), 5.07–5.04 (m, 1 H, H-7*^a^*), 4.99 (d, 1 H, *J*_gem_ = 11.0 Hz, PhC*H_2_*), 4.89 (d, 1 H, *J*_gem_ = 11.0 Hz, PhC*H_2_*), 4.81 (d, 1 H, PhC*H_2_*), 4.78 (d, 1 H, *J*_gem_ = 12.2 Hz, PhC*H_2_*), 4.67 (d, 1 H, PhC*H_2_*), 4.59 (d, 1 H, *J*_1,2_ = 3.5 Hz, H-1*^b^*), 4.52 (d, 1 H, PhC*H_2_*), 4.49 (dd, 1 H, *J*_7,8a_ = 2.2 Hz, *J*_gem_ = 12.2 Hz, H-8a*^a^*), 4.34–4.27 (m, 2 H, H-4*^a^*, CO_2_C*H_2_*CH_2_), 4.11–4.05 (m, 3 H, H-6*^a^*, H-8b*^a^*, CO_2_C*H_2_*CH_2_), 4.01 (t, 1 H, *J*_2,3_ = *J*_3,4_ = 9.2 Hz, H-3*^b^*), 3.83 (near t, 1 H, H-5*^b^*), 3.78 (near dd, 1 H, H-6a*^b^*), 3.46 (dd, 1 H, H-2*^b^*), 3.42 (dd, 1 H, *J*_5,6b_ = 7.5 Hz, H-6b*^b^*), 3.37 (s, 3 H, OCH_3_), 3.25 (dd, 1 H, *J*_4,5_ = 10.0 Hz, H-4*^b^*), 2.39–2.28 (m, 2 H, OCOC*H_2_*CH_2_), 2.14 (near dd, 1 H, H-3*eq^a^*), 2.07–2.02 (m, 4 H, H-3*ax^a^*, Ac), 1.87 (s, 3 H, Ac), 1.70–1.61 (m, 4 H, CO_2_CH_2_C*H_2_*, OCOCH_2_C*H_2_*), 1.54–1.29 (m, 10 H, 5 CH_2_), 1.02 (br s, 21 H, 3 *^i^*Pr); ^13^C NMR (125 MHz, CDCl_3_) δ 160.6, 158.3, 157.6, 155.3, 126.4, 125.83, 125.76, 116.3, 116.2, 115.9, 115.73, 115.67, 115.5, 115.4, 87.0, 85.4, 69.9, 67.5, 66.1, 63.5, 62.8, 61.0, 57.6, 56.7, 56.1, 55.0, 53.9, 53.5, 51.2, 50.1, 42.5, 24.1, 22.1, 16.1, 15.8, 15.6, 15.3, 14.2, 12.6, 8.6, 8.5, 5.7; HRMS (ESI) *m/z*: [M+Na]^+^ Calcd for C_59_H_84_O_16_SiNa 1099.5421; Found 1099.5421. Compound **21**: [α]_D_ −40.6° (c 0.8, CHCl_3_); ^1^H NMR (500 MHz, CDCl_3_) δ 5.90 (t, 1 H, *J*_3,4_ = 1.8 Hz, H-3), 5.49–5.48 (m, 1 H, H-5), 5.12–5.09 (m, 1 H, H-7), 4.81–4.79 (m, 1 H, H-4), 4.61 (dd, 1 H, *J*_7,8a_ = 2.5 Hz, *J*_gem_ = 12.4 Hz, H-8a), 4.37–4.33 (m, 1 H, CO_2_C*H_2_*CH_2_), 4.30 (d, 1 H, *J*_6,7_ = 9.7 Hz, H-6), 4.29–4.24 (m, 2 H, H-8b, CO_2_C*H_2_*CH_2_), 2.39–2.28 (m, 2 H, OCOC*H_2_*CH_2_), 2.09–2.07 (2 s, 6 H, 2 Ac), 1.74–1.71 (m, 2 H, CO_2_CH_2_C*H_2_*), 1.64–1.25 (m, 12 H, 6 CH_2_), 1.14–1.07 (m, 21 H, 3 *^i^*Pr); ^13^C NMR (125 MHz, CDCl_3_) δ 172.7, 170.6, 169.5, 161.6, 143.0, 112.5, 73.3, 67.8, 66.5, 65.1, 63.5, 62.0, 34.4, 29.1, 29.0, 28.5, 28.3, 27.5, 27.3, 25.3, 20.8, 20.7, 17.9, 12.2; HRMS (ESI) *m/z*: [M+Na]^+^ Calcd for C_31_H_52_O_10_SiNa 635.3222; Found 635.3222.

*Methyl 6-O-[7,8-di-O-acetyl-4-O-chloroacetyl-3-deoxy-5-O-(10′-hydroxydecanoyl)-α-d-manno-2-octulopyranosylono-1,10′-lactone]-2,3,4-tri-O-benzyl-α-d-glucopyranoside* (**17**)

Next, 3 Å molecular sieves (100 mg) and NIS (17.3 mg, 77.0 µmol) were added to a solution of **3** (30.5 mg, 51.3 µmol) and **14** (23.8 mg, 51.3 µmol) in CH_2_Cl_2_ (1.0 mL) at room temperature. After stirring for 1 h at −80 °C, TfOH (0.9 µL, 0.01 mmol) was added to the mixture at −80 °C. The reaction mixture was stirred for 96 h at −80 °C while the reaction was monitored by TLC (acetone/toluene = 1/10, developed twice). The reaction was quenched with Et_3_N, and the mixture was filtered through a pad of Celite. The pad was then washed with CHCl_3_. The combined filtrate and washings were washed with satd. Na_2_S_2_O_3_ aq. and brine, dried over Na_2_SO_4_, and concentrated. The resulting residue was purified by gel filtration column chromatography on Sephadex LH-20, using MeOH/CHCl_3_ (1/1) as the eluent, and column chromatography on silica gel, using EtOAc in *n*-hexane (20%) as the eluent, to afford **17** (8.9 mg, 17%) as a colorless syrup: [α]_D_ +60.7° (c 0.8, CHCl_3_); ^1^H NMR (500 MHz, CDCl_3_) δ 7.37–7.25 (m, 15 H, 3 Ph), 5.40–5.36 (m, 1 H, H-4*^a^*), 5.33 (m, 1 H, H-5*^a^*), 5.15–5.12 (m, 1 H, H-7*^a^*), 4.99 (d, 1 H, *J*_gem_ = 10.8 Hz, PhC*H_2_*), 4.91 (d, 1 H, *J*_gem_ = 11.2 Hz, PhC*H_2_*), 4.80 (d, 1 H, PhC*H_2_*), 4.79 (d, 1 H, *J*_gem_ = 12.2 Hz, PhC*H_2_*), 4.67 (d, 1 H, PhC*H_2_*), 4.59 (d, 1 H, *J*_1,2_ = 3.6 Hz, H-1*^b^*), 4.56–4.51 (m, 2 H, PhC*H_2_*, H-8a*^a^*), 4.31–4.27 (m, 1 H, CO_2_C*H_2_*CH_2_), 4.19 (dd, 1 H, *J*_5,6_ = 1.1 Hz, *J*_6,7_ = 9.5 Hz, H-6*^a^*), 4.15–4.11 (m, 1 H, CO_2_C*H_2_*CH_2_), 4.05 (dd, 1 H, *J*_7,8b_ = 4.7 Hz, *J*_gem_ = 12.4 Hz, H-8b*^a^*), 4.02–3.97 (m, 2 H, H-3*^b^*, ClH_2_C), 3.95 (d, 1 H, *J*_gem_ = 15.3 Hz, ClH_2_C), 3.87–3.82 (m, 2 H, H-5*^b^*, H-6a*^b^*), 3.48–3.43 (m, 2 H, H-2*^b^*, H-6b*^b^*), 3.42 (s, 3 H, OCH_3_), 3.25 (dd, 1 H, *J*_3,4_ = 9.0 Hz, *J*_4,5_ = 10.2 Hz, H-4*^b^*), 2.44–2.28 (m, 2 H, OCOC*H_2_*CH_2_), 2.22–2.16 (m, 2 H, H-3*ax^a^*, H-3*eq^a^*), 1.98–1.57 (2 s, 6 H, 2 Ac), 1.76–1.57 (m, 4 H, OCOCH_2_C*H_2_*, CO_2_CH_2_C*H_2_*), 1.48–1.25 (m, 10 H, 5 CH_2_); ^13^C NMR (125 MHz, CDCl_3_) δ 173.1, 170.4, 169.7, 166.7, 152.7, 138.6, 138.1, 138.0, 128.5, 128.44, 128.41, 128.1, 128.03, 127.99, 127.9, 127.8, 127.7, 98.5, 97.7, 94.1, 82.2, 79.8, 78.2, 75.8, 75.0, 73.3, 71.8, 69.6, 68.4, 68.0, 66.4, 63.8, 63.5, 62.0, 55.1, 33.9, 32.2, 29.7, 28.4, 27.9, 27.8, 27.7, 26.5, 25.0, 20.8, 20.7; HRMS (ESI) *m/z*: [M+Na]^+^ Calcd for C_52_H_65_ClO_17_Na 1019.3802; Found 1019.3798.

*Methyl 6-O-[7,8-di-O-acetyl-3-deoxy-5-O-(10′-hydroxydecanoyl)-4-O-(2,2,2-trichloroethoxycarbonyl)-α-d-manno-2-octulopyranosylono-1,10′-lactone]-2,3,4-tri-O-benzyl-α-d-glucopyranoside* (**18**) *and 7,8-di-O-acetyl-2,6-anhydro-3-deoxy-5-O-(10′-hydroxydecanoyl)-4-O-(2,2,2-trichloroethoxycarbonyl)-d-manno-2-octenosono-1,10′-lactone* (**23**)

Next, 3 Å molecular sieves (100 mg) and NIS (17.1 mg, 75.9 µmol) were added to a solution of **4** (35.0 mg, 50.6 µmol) and **14** (23.5 mg, 50.6 µmol) in CH_2_Cl_2_ (1.0 mL) at room temperature. After stirring for 1 h at −80 °C, TfOH (0.9 µL, 0.01 mmol) was added to the mixture at −80 °C. The reaction mixture was stirred for 68 h at −80 °C while the reaction was monitored by TLC (acetone/toluene = 1/10, developed twice). The reaction was quenched with Et_3_N, and the mixture was filtered through a pad of Celite. The pad was then washed with CHCl_3_. The combined filtrate and washings were washed with satd. Na_2_S_2_O_3_ aq. and brine, dried over Na_2_SO_4_, and concentrated. The resulting residue was purified by gel filtration column chromatography on Sephadex LH-20, using MeOH/CHCl_3_ (1/1) as the eluent, and column chromatography on silica gel, using EtOAc in *n*-hexane (20%) as the eluent, to give **18** (11.1 mg, 20%) as a colorless syrup and **23** (24.0 mg, 75%) as a white amorphous mass. Compound **18**: [α]_D_ +59.1° (c 1.2, CHCl_3_); ^1^H NMR (500 MHz, CDCl_3_) δ 7.37–7.26 (m, 15 H, 3 Ph), 5.49 (br s, 1 H, H-5*^a^*), 5.18–5.14 (m, 1 H, H-4*^a^*), 5.13–5.10 (m, 1 H, H-7*^a^*), 4.99 (d, 1 H, *J*_gem_ = 10.8 Hz, PhC*H_2_*), 4.92 (d, 1 H, *J*_gem_ = 11.1 Hz, PhC*H_2_*), 4.82–4.78 (m, 3 H, PhC*H_2_*, CCl_3_CH_2_), 4.69–4.65 (m, 2 H, PhC*H_2_*, CCl_3_CH_2_), 4.59 (d, 1 H, *J*_1,2_ = 3.6 Hz, H-1*^b^*), 4.56–4.51 (m, 2 H, PhC*H_2_*, H-8a*^a^*), 4.32–4.28 (m, 1 H, CO_2_C*H_2_*CH_2_), 4.17 (dd, 1 H, *J*_5,6_ = 1.0 Hz, *J*_6,7_ = 9.5 Hz, H-6*^a^*), 4.14–4.10 (m, 1 H, CO_2_C*H_2_*CH_2_), 4.05 (dd, 1 H, *J*_7,8b_ = 4.6 Hz, *J*_gem_ = 12.4 Hz, H-8b*^a^*), 4.00 (t, 1 H, *J*_2,3_ = *J*_3,4_ = 9.3 Hz, H-3*^b^*), 3.88–3.82 (m, 2 H, H-5*^b^*, H-6a*^b^*), 3.49–3.43 (m, 2 H, H-2*^b^*, H-6b*^b^*), 3.41 (s, 1 H, OCH_3_), 3.26 (near t, 1 H, H-4*^b^*), 2.42–2.25 (m, 3 H, OCOC*H_2_*CH_2_, H-3*eq^a^*), 2.22 (near t, 1 H, H-3*ax^a^*), 1.99–1.89 (2 s, 6 H, 2 Ac), 1.75–1.58 (m, 4 H, OCOCH_2_C*H_2_*, CO_2_CH_2_C*H_2_*), 1.49–1.25 (m, 10 H, 5 CH_2_); ^13^C NMR (125 MHz, CDCl_3_) δ 173.1, 170.4, 169.7, 166.7, 152.7, 138.6, 138.1, 138.0, 128.5, 128.44, 128.41, 128.1, 128.04, 127.99, 127.9, 127.8, 127.7, 98.5, 97.7, 94.1, 82.2, 79.8, 78.2, 75.8, 75.0, 73.3, 71.8, 69.6, 68.4, 68.0, 66.4, 63.8, 63.5, 62.0, 55.1, 33.9, 32.2, 29.7, 28.4, 27.9, 27.8, 27.7, 26.5, 25.0, 20.8, 20.7; HRMS (ESI) *m/z*: [M+Na]^+^ Calcd for C_53_H_65_Cl_3_O_18_Na 1117.3129; Found 1117.3125. Compound **23**: [α]_D_ −4.4° (c 2.5, CHCl_3_); ^1^H NMR (500 MHz, CDCl_3_) δ 5.95 (t, 1 H, *J*_3,4_ = 2.0 Hz, H-3), 5.64–5.62 (m, 1 H, H-5), 5.61–5.59 (m, 1 H, H-4), 5.25–5.22 (m, 1 H, H-7), 4.84 (d, 1 H, *J*_gem_ = 11.8 Hz, CCl_3_CH_2_), 4.70 (d, 1 H, CCl_3_CH_2_), 4.66 (dd, 1 H, *J*_7,8a_ = 2.5 Hz, *J*_gem_ = 12.4 Hz, H-8a), 4.47–4.43 (m, 1 H, CO_2_C*H_2_*CH_2_), 4.39 (d, 1 H, *J*_6,7_ = 9.6 Hz, H-6), 4.26 (dd, 1 H, *J*_7,8b_ = 3.9 Hz, H-8b), 4.25–4.20 (m, 1 H, CO_2_C*H_2_*CH_2_), 2.40–2.30 (m, 2 H, OCOC*H_2_*CH_2_), 2.10–2.06 (2 s, 6 H, 2 Ac), 1.79–1.72 (m, 2 H, CO_2_CH_2_C*H_2_*), 1.65–1.56 (m, 2 H, OCOCH_2_C*H_2_*), 1.46–1.26 (m, 10 H, 5 CH_2_); ^13^C NMR (125 MHz, CDCl_3_) δ 173.1, 170.5, 169.4, 160.7, 153.0, 145.3, 105.9, 94.0, 72.9, 69.9, 67.4, 66.9, 61.7, 60.1, 34.0, 29.1, 28.5, 28.2, 27.6, 27.2, 25.5, 20.8, 20.7; HRMS (ESI) *m/z*: [M+Na]^+^ Calcd for C_25_H_33_Cl_3_O_12_Na 653.0930; Found 653.0935.

*Methyl 6-O-[7,8-di-O-acetyl-4-O-benzyloxycarbonyl-3-deoxy-5-O-(10′-hydroxydecanoyl)-α-d-manno-2-octulopyranosylono-1,10′-lactone]-2,3,4-tri-O-benzyl-α-d-glucopyranoside* (**19**) *and 7,8-di-O-acetyl-2,6-anhydro-4-O-benzyloxycarbonyl-3-deoxy-5-O-(10′-hydroxydecanoyl)-d-manno-2-octenosono-1,10′-lactone* (**24**)

Next, 3 Å molecular sieves (100 mg) and NIS (16.7 mg, 74.3 µmol) were added to a solution of **5** (32.3 mg, 49.5 µmol) and **14** (23.0 mg, 49.5 µmol) in CH_2_Cl_2_ (1.0 mL) at room temperature. After stirring for 1 h at −80 °C, TfOH (0.9 µL, 0.01 mmol) was added to the mixture at −80 °C. The reaction mixture was stirred for 42 h at −80 °C while the reaction was monitored by TLC (acetone/toluene = 1/10, developed twice). The reaction was quenched with Et_3_N, and the mixture was filtered through a pad of Celite. The pad was washed with CHCl_3_. The combined filtrate and washings were washed with satd. Na_2_S_2_O_3_ aq. and brine, dried over Na_2_SO_4_, and concentrated. The resulting residue was purified by gel filtration column chromatography on Sephadex LH-20, using MeOH/CHCl_3_ (1/1) as the eluent, and column chromatography on silica gel, using EtOAc in *n*-hexane (22%) and EtOAc in *n*-hexane (35%) as the eluent, to afford **19** (14.8 mg, 28%) and **24** (approximately 20.4 mg, approximately 70%) as colorless syrups. Compound **19**: [α]_D_ +61.0° (c 1.5, CHCl_3_); ^1^H NMR (500 MHz, CDCl_3_) δ 7.38–7.25 (m, 20 H, 4 Ph), 5.14 (near d, 1 H, H-5*^a^*), 5.19–5.09 (m, 4 H, H-4*^a^*, H-7*^a^*, PhC*H_2_*), 4.98 (d, 1 H, *J*_gem_ = 10.8 Hz, PhC*H_2_*), 4.91 (d, 1 H, *J*_gem_ = 11.1 Hz, PhC*H_2_*), 4.81 (d, 1 H, PhC*H_2_*), 4.79 (d, 1 H, *J*_gem_ = 12.1 Hz, PhC*H_2_*), 4.67 (d, 1 H, PhC*H_2_*), 4.59 (d, 1 H, *J*_1,2_ = 3.6 Hz, H-1*^b^*), 4.54 (d, 1 H, PhC*H_2_*), 4.53 (dd, 1 H, *J*_7,8a_ = 2.3 Hz, *J*_gem_ = 12.3 Hz, H-8a*^a^*), 4.30–4.26 (m, 1 H, CO_2_C*H_2_*CH_2_), 4.17 (dd, 1 H, *J*_5,6_ = 1.1 Hz, *J*_6,7_ = 9.5 Hz, H-6*^a^*), 4.13–4.09 (m, 1 H, CO_2_C*H_2_*CH_2_), 4.05 (dd, 1 H, *J*_7,8b_ = 4.5 Hz, H-8b*^a^*), 4.00 (t, 1 H, *J*_2,3_ = *J*_3,4_ = 9.2 Hz, H-3*^b^*), 3.86–3.82 (m, 2 H, H-5*^b^*, H-6a*^b^*), 3.49–3.43 (m, 2 H, H-2*^b^*, H-6b*^b^*), 3.41 (s, 3 H, OCH_3_), 3.26 (dd, 1 H, *J*_4,5_ = 10.2 Hz, H-4*^b^*), 2.41–2.29 (m, 2 H, OCOC*H_2_*CH_2_), 2.22 (dd, 1 H, *J*_3*eq*,4_ = 5.1 Hz, *J*_gem_ = 12.7 Hz, H-3*eq^a^*), 2.16 (t, 1 H, H-3*ax^a^*), 2.00–1.88 (2 s, 6 H, 2 Ac), 1.75–1.61 (m, 4 H, CO_2_CH_2_C*H_2_*, OCOCH_2_C*H_2_*), 1.48–1.20 (m, 10 H, 5 CH_2_); ^13^C NMR (125 MHz, CDCl_3_) δ 173.1, 170.4, 169.7, 166.8, 153.9, 138.6, 138.1, 138.0, 135.0, 128.6, 128.53, 128.50, 128.43, 128.39, 128.3, 128.1, 128.03, 127.98, 127.9, 127.74, 127.68, 98.6, 97.7, 82.2, 79.8, 78.3, 75.8, 75.0, 73.3, 70.6, 69.9, 69.6, 68.5, 68.0, 66.3, 64.2, 63.5, 62.1, 55.2, 34.0, 32.3, 28.4, 27.9, 27.8, 27.6, 26.5, 25.0, 20.8, 20.7; HRMS (ESI) *m/z*: [M+Na]^+^ Calcd for C_58_H_70_O_18_Na 1077.4454; Found 1077.4453. Compound **24**: [α]_D_ −7.0° (c 1.8, CHCl_3_); ^1^H NMR (500 MHz, CDCl_3_) δ 7.40–7.33 (m, 5 H, Ph), 5.92 (t, 1 H, *J*_3,4_ = 1.9 Hz, H-3), 5.62 (near d, 1 H, H-5), 5.57–5.55 (m, 1 H, H-4), 5.23–5.21 (m, 2 H, H-7, PhC*H_2_*), 5.16 (d, 1 H, *J*_gem_ = 12.1 Hz, PhC*H_2_*), 4.65 (dd, 1 H, *J*_7,8a_ = 2.4 Hz, *J*_gem_ = 12.4 Hz, H-8a), 4.44–4.40 (m, 1 H, CO_2_C*H_2_*CH_2_), 4.38 (d, 1 H, *J*_6,7_ = 9.6 Hz, H-6), 4.27–4.20 (m, 2 H, H-8b, CO_2_C*H_2_*CH_2_), 2.40–2.29 (m, 2 H, OCOC*H_2_*CH_2_), 2.09–2.06 (2 s, 6 H, 2 Ac), 1.77–1.69 (m, 2 H, CO_2_CH_2_C*H_2_*), 1.62–1.51 (m, 2 H, OCOCH_2_C*H_2_*), 1.46–1.25 (m, 10 H, 5 CH_2_); ^13^C NMR (125 MHz, CDCl_3_) δ 173.0, 170.5, 169.4, 160.9, 154.2, 144.9, 134.9, 128.6, 128.3, 106.7, 73.0, 70.2, 69.0, 67.5, 66.8, 61.8, 60.4, 34.1, 29.1, 28.4, 28.3, 27.6, 27.2, 25.5, 20.8, 20.7; HRMS (ESI) *m/z*: [M+Na]^+^ Calcd for C_30_H_38_O_12_Na 613.2255; Found 613.2256.

*Methyl 6-O-[7,8-di-O-acetyl-3-deoxy-5-O-(10′-hydroxydecanoyl)-α-d-manno-2-octulopyranosylono-1,10′-lactone]-2,3,4-tri-O-benzyl-α-d-glucopyranoside* (**25**)

To a solution of **16** (44.1 mg, 41.0 µmol) in pyridine (0.8 mL), HF pyridine (82.0 µL) was added at 0 °C. After stirring for 24 h at room temperature, HF pyridine (82.0 µL) was added, and the mixture was stirred for a total of 42 h while the reaction was monitored by TLC (EtOAc/*n*-hexane = 2/3). Satd. NaHCO_3_ aq. was added to the reaction mixture at 0 °C. The mixture was diluted with CHCl_3_, washed with satd. NaHCO_3_ aq. and brine, dried over Na_2_SO_4_, and concentrated. The residue was purified by column chromatography on silica gel, using EtOAc in *n*-hexane (40%) and acetone in toluene (9%) as eluents, to afford **25** (36.6 mg, 97%) as a colorless syrup: [α]_D_ +43.4° (c 1.8, CHCl_3_); ^1^H NMR (500 MHz, CDCl_3_) δ 7.37–7.26 (m, 15 H, 3 Ph), 5.20 (br s, 1 H, H-5*^a^*), 5.16–5.13 (m, 1 H, H-7*^a^*), 4.99 (d, 1 H, *J*_gem_ = 10.8 Hz, PhC*H_2_*), 4.91 (d, 1 H, *J*_gem_ = 11.1 Hz, PhC*H_2_*), 4.80 (d, 1 H, PhC*H_2_*), 4.79 (d, 1 H, *J*_gem_ = 12.2 Hz, PhC*H_2_*), 4.67 (d, 1 H, PhC*H_2_*), 4.57 (d, 1 H, *J*_1,2_ = 3.6 Hz, H-1*^b^*), 4.55–4.52 (m, 2 H, H-8a*^a^*, PhC*H_2_*), 4.31–4.27 (m, 1 H, CO_2_C*H_2_*CH_2_), 4.24–4.22 (m, 1 H, H-4*^a^*), 4.12–4.07 (m, 3 H, H-6*^a^*, H-8b*^a^*, CO_2_C*H_2_*CH_2_), 4.00 (t, 1 H, *J*_2,3_ = *J*_3,4_ = 9.2 Hz, H-3*^b^*), 3.83–3.80 (m, 2 H, H-5*^b^*, H-6a*^b^*), 3.49–3.41 (m, 2 H, H-2*^b^*, H-6b*^b^*), 3.38 (s, 3 H, OCH_3_), 3.29 (near t, 1 H, H-4*^b^*), 2.47–2.32 (m, 2 H, OCOC*H_2_*CH_2_), 2.19 (dd, 1 H, *J*_3*eq*,4_ = 4.7 Hz, *J*_gem_ = 12.8 Hz, H-3*eq^a^*), 2.12 (d, 1 H, *J*_4,OH_ = 3.2 Hz, OH*^b^*), 2.01 (s, 3 H, Ac), 1.94–1.89 (m, 4 H, H-3*ax^a^*, Ac), 1.71–1.63 (m, 4 H, CO_2_CH_2_C*H_2_*, OCOCH_2_C*H_2_*), 1.47–1.25 (m, 10 H, 5 CH_2_); ^13^C NMR (125 MHz, CDCl_3_) δ 174.9, 170.5, 169.7, 167.2, 138.6, 138.1, 138.0, 128.5, 128.42, 128.39, 128.1, 128.0, 127.9, 127.8, 127.75, 127.67, 98.9, 97.7, 82.2, 79.8, 78.1, 75.8, 74.9, 73.3, 69.6, 68.5, 68.3, 67.7, 66.2, 65.4, 62.3, 55.0, 34.9, 34.2, 28.4, 27.9, 27.8, 27.6, 26.5, 25.1, 20.8, 20.7; HRMS (ESI) *m/z*: [M+Na]^+^ Calcd for C_50_H_64_O_16_Na 943.4087; Found 943.4082.

*Methyl 6-O-{7,8-di-O-acetyl-4-O-[7,8-di-O-acetyl-3-deoxy-5-O-(10′-hydroxydecanoyl)-4-O-triisopropylsilyl-α-d-manno-2-octulopyranosylono-1,10′-lactone]-3-deoxy-5-O-(10′’-hydroxydecanoyl)-α-d-manno-2-octulopyranosylono-1,10′’-lactone}-2,3,4-tri-O-benzyl-α-d-glucopyranoside* (**26**)

3 Å Molecular sieves (80 mg) and NIS (28.6 mg, 127 µmol) were added to a solution of **2** (56.9 mg, 84.4 µmol) and **25** (38.8 mg, 42.2 µmol) in CH_2_Cl_2_ (0.8 mL) at room temperature. After stirring for 1 h at −80 °C, TfOH in MeCN (7.4 µL, 8.44 µmol, 10-fold dilution) was added to the mixture at −80 °C. The reaction mixture was stirred for 22 h at −80 °C while the reaction was monitored by TLC (acetone/toluene = 1/14, developed twice). The reaction was quenched with Et_3_N, and the mixture was filtered through a pad of Celite. The pad was washed with CHCl_3_, and the combined filtrate and washings were washed with satd. Na_2_S_2_O_3_ aq. and brine, dried over Na_2_SO_4_, and concentrated. The resulting residue was purified by column chromatography on silica gel, using EtOAc in *n*-hexane (25%) as the eluent, to afford **26** (45.4 mg, 70%) as a colorless syrup: [α]_D_ +77.8° (c 2.7, CHCl_3_); ^1^H NMR (500 MHz, CDCl_3_) δ 7.38–7.24 (m, 15 H, 3 Ph), 5.32 (br s, 1 H, H-5*^a^*), 5.30 (br s, 1 H, H-5*^b^*), 5.05–4.97 (m, 3 H, H-7*^a^*, H-7*^b^*, PhC*H_2_*), 4.94 (d, 1 H, *J*_gem_ = 11.2 Hz, PhC*H_2_*), 4.85 (dd, 1 H, *J*_7,8a_ = 2.5 Hz, *J*_gem_ = 12.4 Hz, H-8a*^b^*), 4.80–4.73 (m, 3 H, H-4*^b^*, PhC*H_2_*), 4.67 (d, 1 H, *J*_gem_ = 12.1 Hz, PhC*H_2_*), 4.60 (d, 1 H, *J*_1,2_ = 3.5 Hz, H-1*^c^*), 4.53 (d, 1 H, PhC*H_2_*), 4.47 (dd, 1 H, *J*_7,8a_ = 2.3 Hz, *J*_gem_ = 12.3 Hz, H-8a*^a^*), 4.38–4.30 (m, 2 H, CO_2_C*H_2_*CH_2_), 4.19–4.15 (m, 2 H, H-4*^a^*, H-6*^b^*), 4.12–3.98 (m, 6 H, CO_2_C*H_2_*CH_2_, H-6*^a^*, H-8b*^a^*, H8b*^b^*, H-3*^c^*), 3.78–3.72 (m, 2 H, H-5*^c^*, H-6a*^c^*), 3.47–3.43 (m, 2 H, H-2*^c^*, H-6b*^c^*), 3.41 (s, 3 H, OCH_3_), 3.22 (near t, 1 H, H-4*^c^*), 2.38–2.22 (m, 4 H, OCOC*H_2_*CH_2_), 2.19–2.13 (m, 2 H, H-3*ax^a^*, H-3*eq^b^*), 2.10 (s, 3 H, Ac), 2.02–1.97 (m, 8 H, H-3*eq^a^*, H-3*ax^b^*, 2 Ac), 1.82 (s, 3 H, Ac), 1.71–1.57 (m, 8 H, CO_2_CH_2_C*H_2_*, OCOCH_2_C*H_2_*), 1.47–1.25 (m, 20 H, 10 CH_2_), 1.03 (br s, 21 H, 3 *^i^*Pr); ^13^C NMR (125 MHz, CDCl_3_) δ 172.6, 172.5, 171.0, 170.6, 169.9, 169.8, 167.6, 167.0, 138.7, 138.2, 128.43, 128.37, 128.3, 128.1, 127.94, 127.85, 127.8, 127.6, 98.9, 98.0, 97.5, 82.2, 80.0, 78.1, 75.7, 74.8, 73.2, 69.8, 69.5, 68.7, 68.5, 68.3, 67.2, 66.3, 66.2, 66.1, 65.5, 63.3, 62.3, 61.0, 55.1, 36.0, 34.3, 34.3, 34.2, 28.5, 28.4, 28.1, 27.9, 27.8, 27.7, 27.6, 27.4, 26.8, 26.3, 24.9, 20.81, 20.77, 20.76, 20.6, 18.0, 17.9, 12.2; HRMS (ESI) *m/z*: [M+Na]^+^ Calcd for C_81_H_116_O_26_SiNa 1555.7416; Found 1555.7416.

## 4. Conclusions

The attachment of an electron-donating TIPS group at the 4-OH position boosted the reactivity of the macrobicylic Kdo donor in comparison with the prototype 4-*O*-Ac Kdo donor **1**. Furthermore, the chemoselectivity of the removal of the TIPS group allowed the conversion of the Kdo donor into a 4-OH Kdo acceptor after the glycosidation reaction. The 4-*O*-TIPS Kdo donor **2** expands the scope of macrobicyclic Kdo donors in the chemical synthesis of α-Kdo-containing glycans.

## Figures and Tables

**Table 1:** α−glycosylation parameters using macrobicyclic Kdo donors.

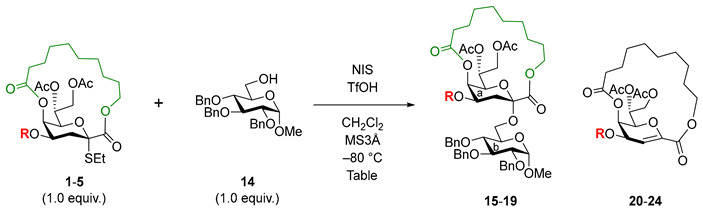						
Entry	Donor	R	Time	Yield		Recovered 14
				α-Glycoside	2,3-ene	
1	**1**	Ac	35 h	84% (**15**)	15% (**20**)	16%
2	**2**	TIPS	22 h	76% (**16**)	20% (**21**)	21%
3	**3**	CAc	96 h	17% (**17**)	<18% (**22**) ^a^	<82% ^a^
4	**4**	Troc	68 h	20% (**18**)	75% (**23**)	77%
5	**5**	Cbz	42 h	28% (**19**)	<70% (**24**) ^a^	<74% ^a^

^a^ obtained as inseparable mixture.

## Data Availability

Not applicable.

## Supplementary Materials

The following supporting information can be downloaded at: https://www.mdpi.com/article/10.3390/molecules28010102/s1, copies of ^1^H and ^13^C NMR spectra of the new compounds.

## References

[1] Di Lorenzo F., Duda K.A., Lanzetta D.R., Silipo A., De Castro C., Molinaro A. (2022). A journey from structure to function of bacterial lipopolysaccharides. Chem. Rev..

[2] Whitfield C., Wear S.S., Sande C. (2020). Assembly of bacterial capsular polysaccharides and exopolysaccharides. Annu. Rev. Microbiol..

[3] Cipolla L., Gabrielli L., Bini D., Russo L., Shaikh N. (2010). Kdo: A critical monosaccharide for bacteria viability. Nat. Prod. Rep..

[4] Pradhan T.K., Mong K.K.T. (2015). Glycosylation chemistry of 3-deoxy-d-manno-oct-2-ulosonic acid (Kdo) donors. Isr. J. Chem..

[5] Kosma P. (2016). Progress in Kdo-glycoside chemistry. Tetrahedron Lett..

[6] Imoto M., Kusunose N., Matsuura Y., Kusumoto S., Shiba T. (1987). Preparation of novel pyranosyl fluorides of 3-deoxy-D-manno-2-octulosonic acid (KDO) feasible for synthesis of KDO α-glycosides. Tetrahedron Lett..

[7] Yoshizaki H., Fukuda N., Sato K., Oikawa M., Fukase K., Suda Y., Kusu-moto S. (2001). First total synthesis of the Re-type lipopoly-saccharide. Angew. Chem. Int. Ed..

[8] Ichiyanagi T., Fukunaga M., Tagashira R., Hayashi S., Nanjo M., Yamasaki R. (2011). A new Kdo derivative for the synthesis of an inner-core disaccharide of lipopolysaccharides and lipooligosaccharides. Tetrahedron.

[9] Shimoyama A., Saeki A., Tanimura N., Tsutsui H., Miyake K., Suda Y., Fujimoto Y., Fukase K. (2011). Chemical synthesis of *Helicobacter pylori* lipopolysaccharide partial structures and their selective proinflammatory responses. Chem. Eur. J..

[10] Shimoyama A., Fujimoto Y., Fukase K. (2011). Stereoselective glycosylation of 3-deoxy-d-manno-2-octulosonic acid with batch and microfluidic methods. Synlett.

[11] Huang J.-S., Huang W., Meng X., Wang X., Gao P.-C., Yang J.-S. (2015). Stereoselective synthesis of α-3-deoxy-d-*manno*-oct-2-ulosonic acid (α-Kdo) glycosides using 5,7-*O*-di-*tert*-butylsilylene-protected Kdo ethyl thioglycoside donors. Angew. Chem. Int. Ed..

[12] Ikeda K., Akamatsu S., Achiwa K. (1990). A new synthesis of α-glycosidically-linked disaccharides using 2 α-chloro-3 β-phenylthio KDO derivatives. Chem. Pharm. Bull..

[13] Pokorny B., Kosma P. (2015). Synthesis of Chlamydia lipopolysaccharide haptens through the use of α-specific 3-iodo-Kdo fluoride glycosyl donors. Chem. Eur. J..

[14] Pokorny B., Kosma P. (2015). First and stereoselective synthesis of an α-(2→5)-linked disaccharide of 3-deoxy-d-*manno*-oct-2-ulosonic acid (Kdo). Org. Lett..

[15] Huang W., Zhou Y.-Y., Pan X.-L., Zhou X.-Y., Lei J.-C., Liu D.-M., Chu Y., Yang J.-S. (2018). Stereodirecting effect of C5-carboxylate substituents on the glycosylation stereochemistry of 3-deoxy-d-*manno*-oct-2-ulosonic acid (Kdo) thioglycoside donors: Stereoselective synthesis of α- and β-Kdo glycosides. J. Am. Chem. Soc..

[16] Ikeda K., Akamatsu S., Achiwa K. (1989). A new and stereospecific approach to Kdo-containing disaccharides using phenylselenyl triflate. Carbohydr. Res..

[17] van der Klein P.A.M., Boons G.J.P.H., Veeneman G.H., van der Marel G.A., van Boom J.H. (1990). Iodonium ion promoted cyclization: A convenient approach to glycosyl donors of 3-deoxy-d-*manno*-2-octulosonic acid (KDO). Synlett.

[18] Tanaka H., Takahashi D., Takahashi T. (2006). Stereoselective synthesis of oligo-α(2,8)-3-deoxy-d-*manno*-2-octulosonic acid derivatives. Angew. Chem. Int. Ed..

[19] Mannerstedt K., Ekelöf K., Oscarson S. (2007). Evaluation of thioglycosides of Kdo as glycosyl donors. Carbohydr. Res..

[20] Yoshida F., Yoshinaka H., Tanaka H., Hanashima S., Yamaguchi Y., Ishihara M., Saburomaru M., Kato Y., Saito R., Ando H. (2019). Synthesis of the core oligosaccharides of lipooligosaccharides from *Campylobacter jejuni*: A putative cause of Guillain-Barré syndrome. Chem. Eur. J..

[21] Lou Q., Hua Q., Zhang L., Yang Y. (2020). Dimethylformamide-modulated Kdo glycosidation for stereoselective synthesis of α-Kdo glycosides. Org. Lett..

[22] Hamajima S., Komura N., Tanaka H.-N., Imamura A., Ishida H., Noguchi H., Ichiyanagi T., Ando H. (2022). Full stereocontrol in α-glycosidation of 3-deoxy-d-manno-2-octulosonic acid (Kdo) using macrobicyclic glycosyl donors. Org. Lett..

[23] Zhang Z., Ollmann I.R., Ye X.-S., Wischnat R., Baasov T., Wong C.-H. (1999). Programmable one-pot oligosaccharide synthesis. J. Am. Chem. Soc..

[24] Chang C.-W., Wu C.-H., Lin M.-H., Liao P.-H., Chang C.-C., Chuang H.-H., Lin S.-C., Lam S., Verma V.P., Hsu C.-P. (2019). Establishment of guideline for the control of glycosylation reactions and intermediates by quantitative assessment of reactivity. Angew. Chem. Int. Ed..

[25] Ishiwata A., Ito Y. (2003). Preparation of sialyl donors carrying functionalized ester substituents: Effect on the selectivity of glycosylation. Synlett.

[26] Kurihara T., Nakajima Y., Mitsunobu O. (1976). Synthesis of lactones and cycloalkanes. Cyclization of ω-hydroxy acids and ethyl α-cyano-ω-hydroxycarboxylates. Tetrahedron Lett..

