# Supplementary material for: Investigation of the Protection of the C4 Hydroxyl Group in Macrobicyclic Kdo Donors
Source: Molecules. 2022 Dec 23;28(1):102. doi: 10.3390/molecules28010102 (PMC9822203; doi:10.3390/molecules28010102)
Supplement: Supplementary file 1 [file molecules-28-00102-s001.zip › molecules-2102724-supplementary.pdf]

This PDF file includes:

Copies of ^{1}H and ^{13}C NMR spectra

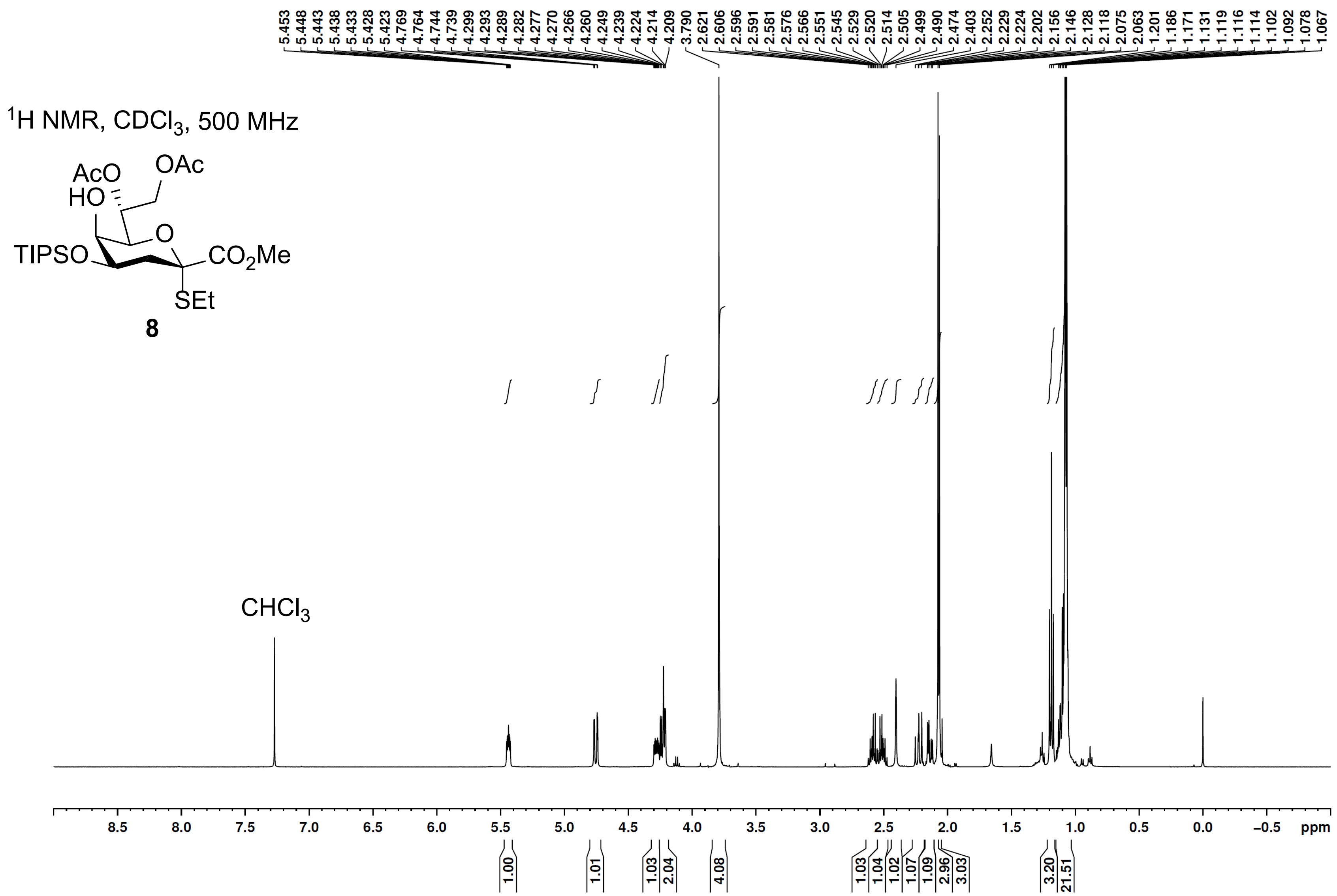

^{1}H NMR, $CDCl_3$, 500 MHz
AcO
OAc
HO
O
TIPSO
CO_2Me
SEt
8
$CHCl_3$
5.453 5.448 5.443 5.438 5.433 5.428 5.423 4.769 4.764 4.744 4.739 4.299 4.293 4.289 4.282 4.277 4.270 4.266 4.260 4.249 4.239 4.224 4.214 4.209 3.790 2.621 2.606 2.596 2.591 2.581 2.576 2.566 2.551 2.545 2.529 2.520 2.514 2.505 2.499 2.490 2.474 2.403 2.252 2.229 2.224 2.202 2.156 2.146 2.128 2.118 2.075 2.063 1.201 1.186 1.171 1.131 1.119 1.116 1.114 1.102 1.092 1.078 1.067
8.5 8.0 7.5 7.0 6.5 6.0 5.5 5.0 4.5 4.0 3.5 3.0 2.5 2.0 1.5 1.0 0.5 0.0 –0.5 ppm
1.00 1.01 1.03 2.04 4.08 1.03 1.04 1.02 1.07 1.09 2.96 3.03 3.20 21.51

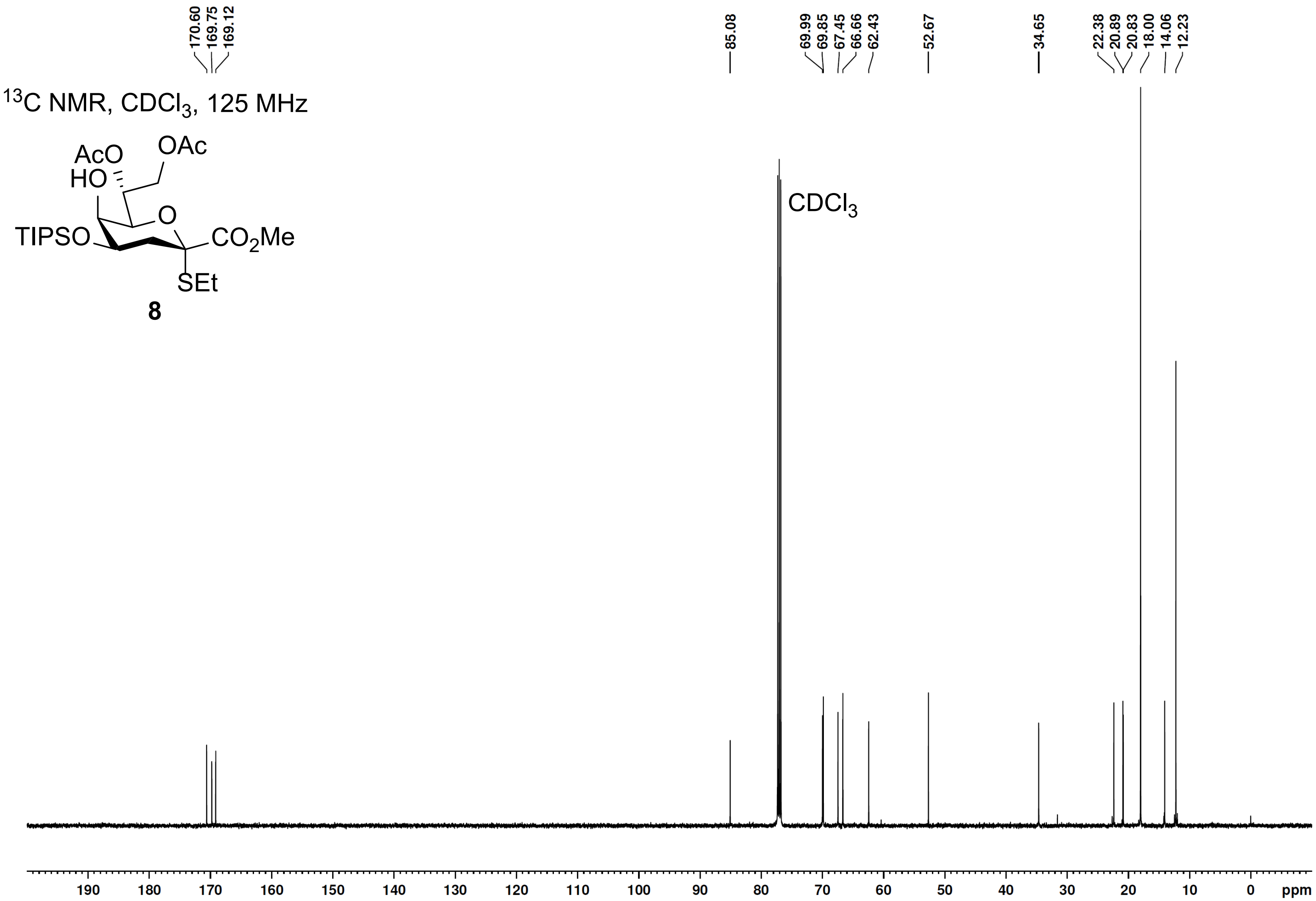

13C NMR, CDCl3, 125 MHz
AcO
OAc
HO
O
TIPSO
CO2Me
SEt
8
CDCl3
170.60
169.75
169.12
85.08
69.99
69.85
67.45
66.66
62.43
52.67
34.65
22.38
20.89
20.83
18.00
14.06
12.23
190 180 170 160 150 140 130 120 110 100 90 80 70 60 50 40 30 20 10 0 ppm

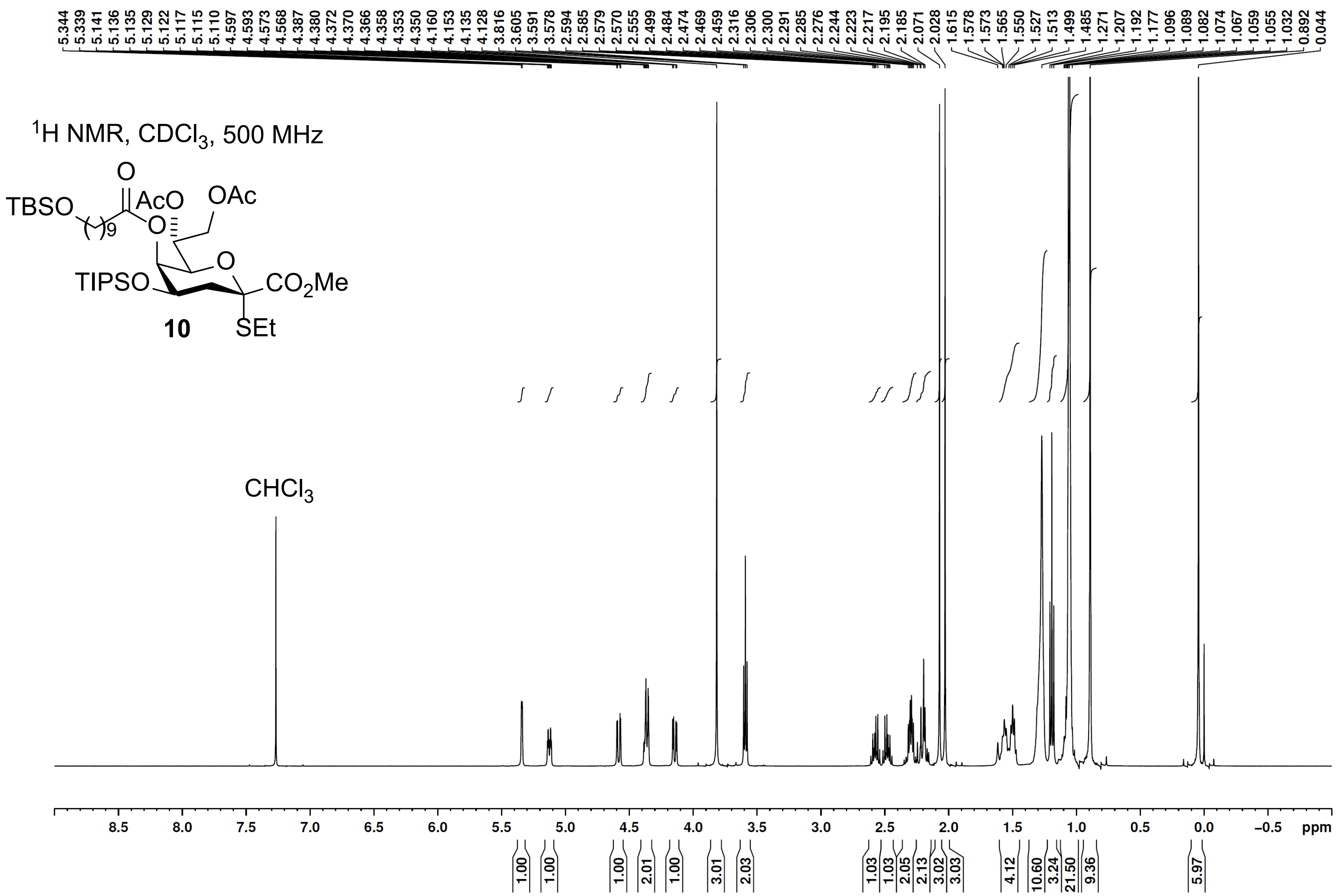
^1H NMR, $CDCl_3$, 500 MHz
TBSO
AcO
OAc
TIPSO
CO_2Me
SEt
10
$CHCl_3$
ppm

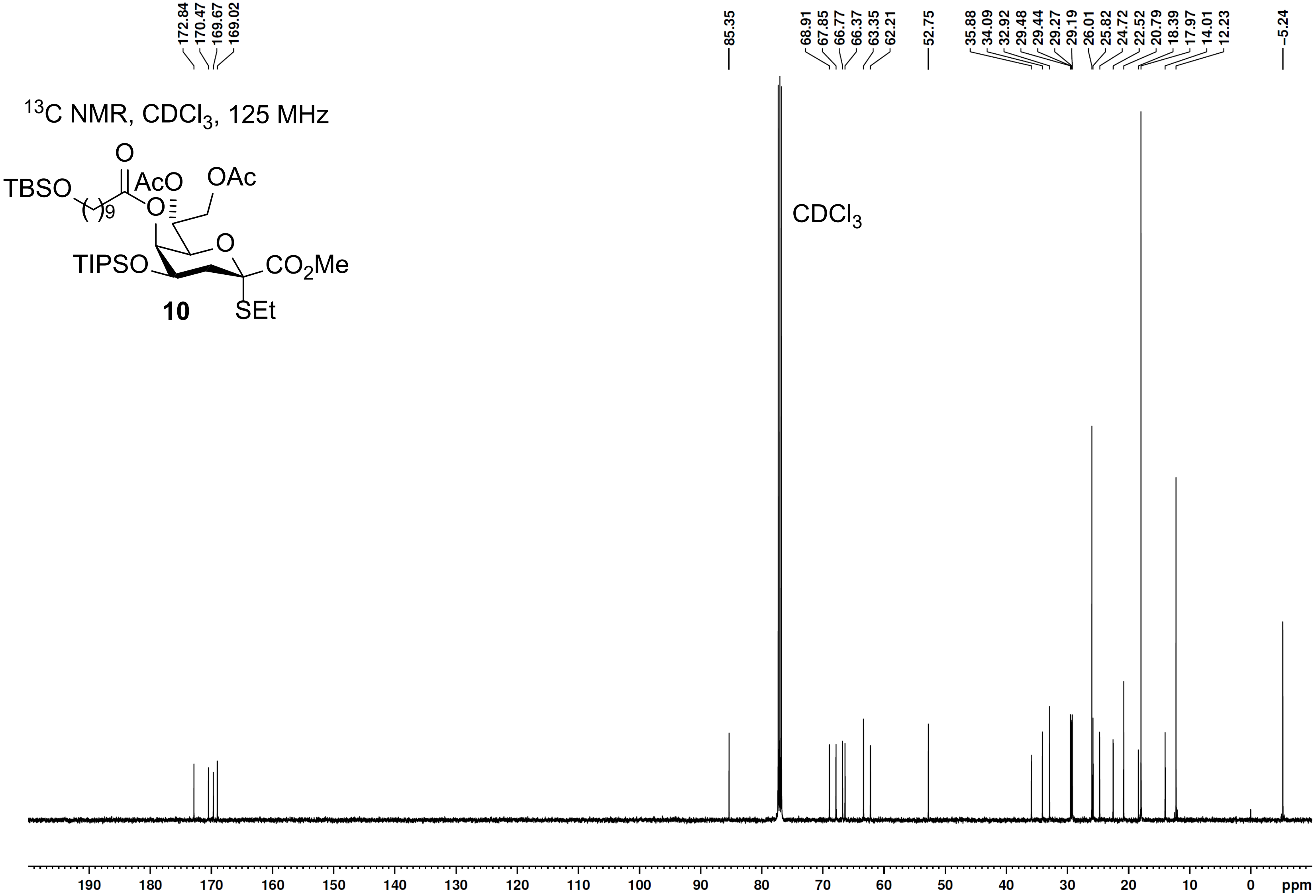

^{13}C NMR, CDCl$_3$, 125 MHz
TBSO
9
O
AcO
OAc
O
TIPSO
CO$_2$Me
SEt
10
CDCl$_3$
172.84
170.47
169.67
169.02
85.35
68.91
67.85
66.77
66.37
63.35
62.21
52.75
35.88
34.09
32.92
29.48
29.44
29.27
29.19
26.01
25.82
24.72
22.52
20.79
18.39
17.97
14.01
12.23
-5.24
190 180 170 160 150 140 130 120 110 100 90 80 70 60 50 40 30 20 10 0 ppm

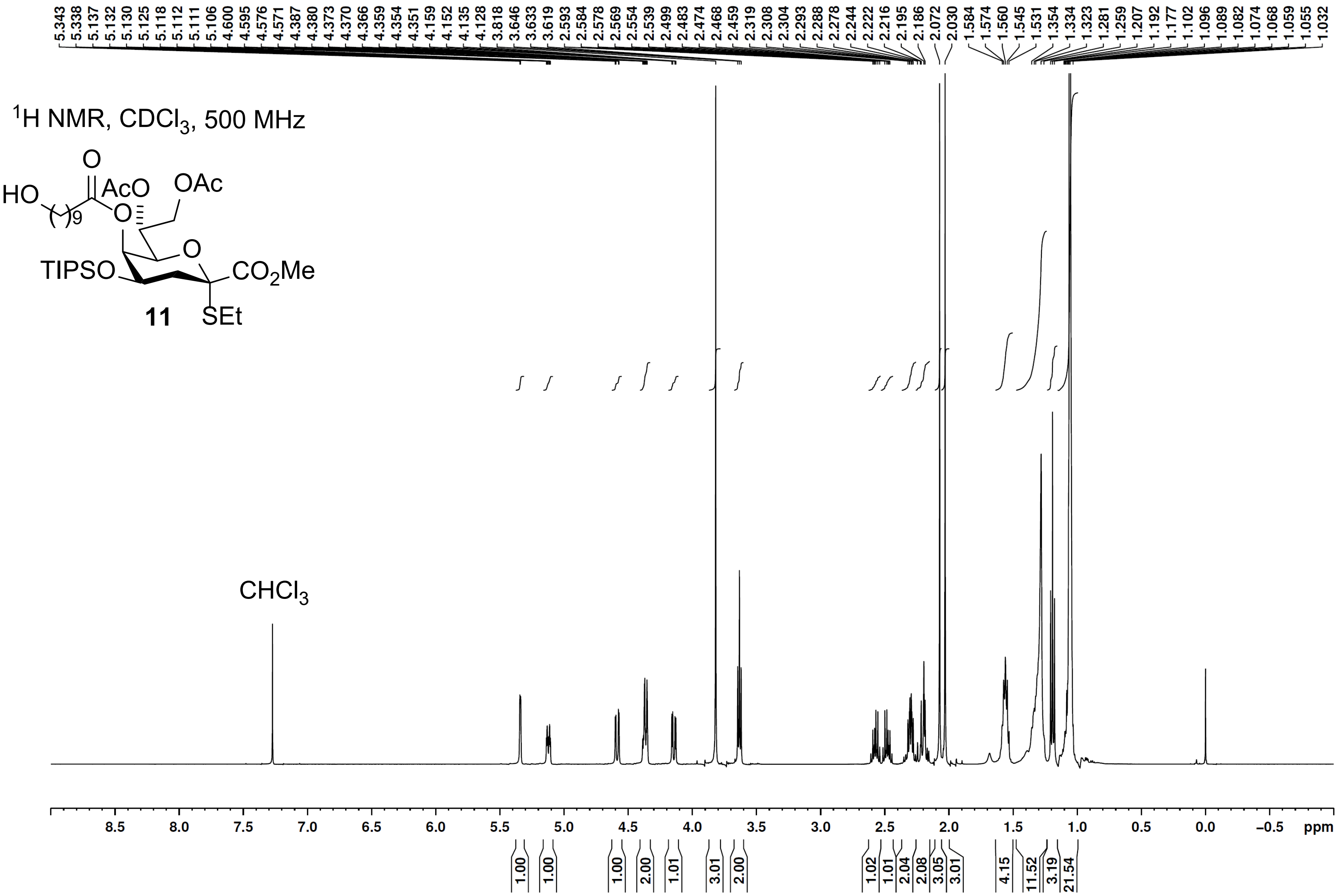
5.343 5.338 5.137 5.132 5.130 5.125 5.118 5.112 5.111 5.106 4.600 4.595 4.576 4.571 4.387 4.380 4.373 4.370 4.366 4.359 4.354 4.351 4.159 4.152 4.135 4.128 3.818 3.646 3.633 3.619 2.593 2.584 2.578 2.569 2.554 2.539 2.499 2.483 2.474 2.468 2.459 2.319 2.308 2.304 2.293 2.288 2.278 2.244 2.222 2.216 2.195 2.186 2.072 2.030 1.584 1.574 1.560 1.545 1.531 1.354 1.334 1.323 1.281 1.259 1.207 1.192 1.177 1.102 1.096 1.089 1.082 1.074 1.068 1.059 1.055 1.032
1H NMR, CDCl3, 500 MHz
O
HO
AcO
OAc
O
9
O
TIPSO
CO2Me
11
SEt
CHCl3
8.5 8.0 7.5 7.0 6.5 6.0 5.5 5.0 4.5 4.0 3.5 3.0 2.5 2.0 1.5 1.0 0.5 0.0 –0.5 ppm
1.00 1.00 1.00 2.00 1.01 3.01 2.00 1.02 1.01 2.04 2.08 3.05 3.01 4.15 11.52 3.19 21.54

^{13}C NMR, $CDCl_3$, 125 MHz

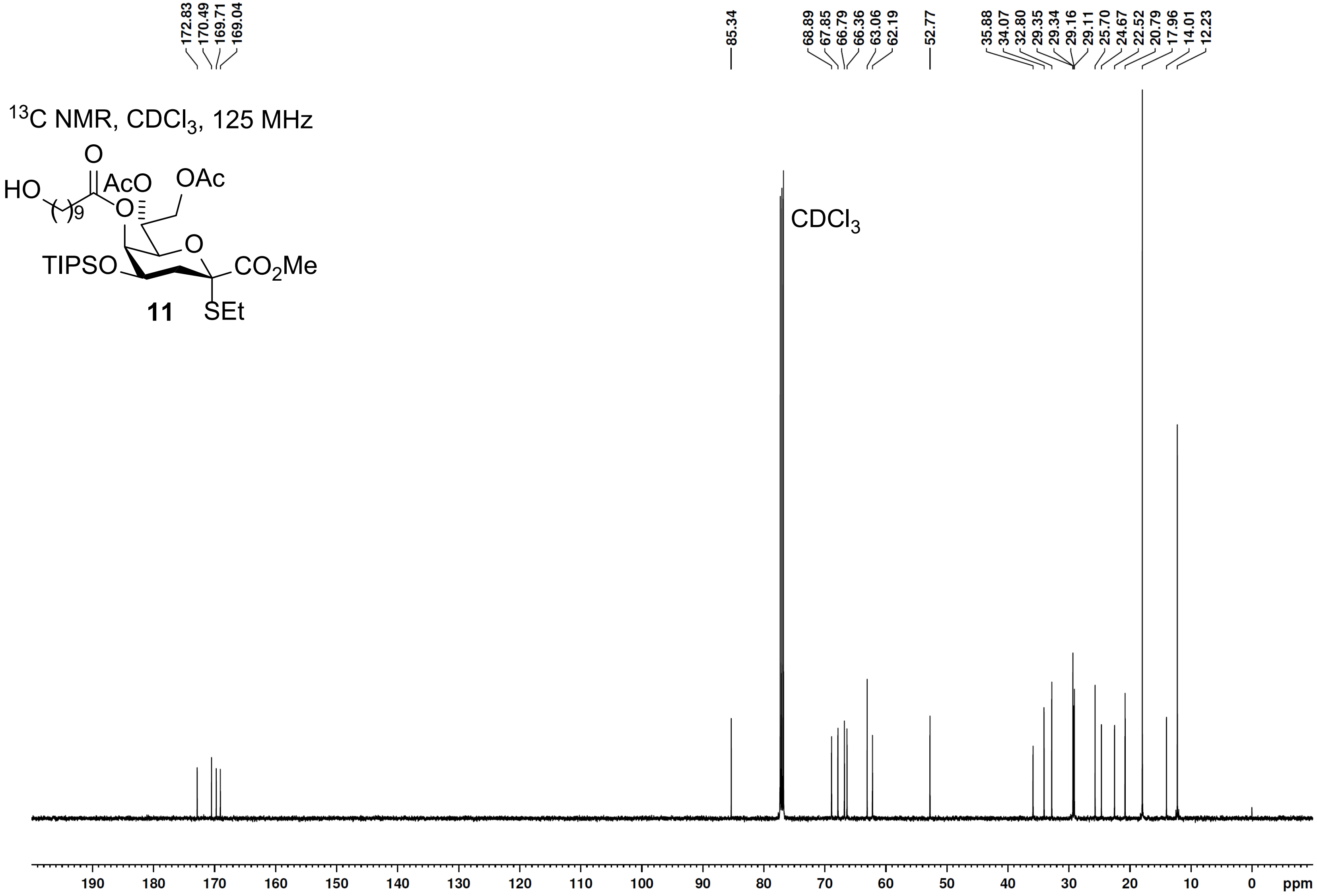

11

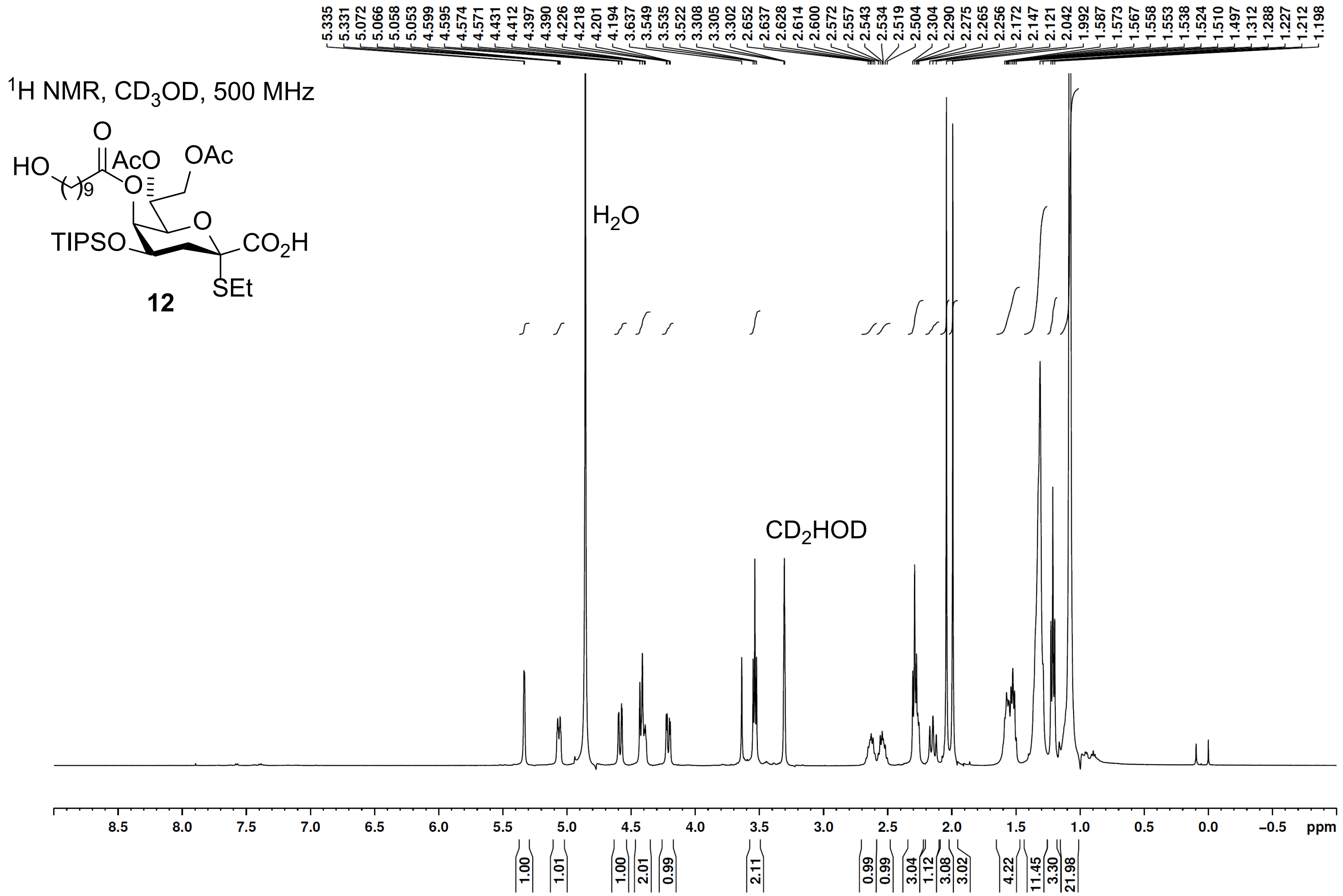

1H NMR, CD3OD, 500 MHz
HO
O
AcO
OAc
9
O
O
TIPSO
CO2H
SEt
12
H2O
CD2HOD
5.335 5.331 5.072 5.066 5.058 5.053 4.599 4.595 4.574 4.571 4.431 4.412 4.397 4.390 4.226 4.218 4.201 4.194 3.637 3.549 3.535 3.522 3.308 3.305 3.302 2.652 2.637 2.628 2.614 2.600 2.572 2.557 2.543 2.534 2.519 2.504 2.304 2.290 2.275 2.265 2.256 2.172 2.147 2.121 2.042 1.992 1.587 1.573 1.567 1.558 1.553 1.538 1.524 1.510 1.497 1.312 1.288 1.227 1.212 1.198
8.5 8.0 7.5 7.0 6.5 6.0 5.5 5.0 4.5 4.0 3.5 3.0 2.5 2.0 1.5 1.0 0.5 0.0 –0.5 ppm
1.00 1.01 1.00 2.01 0.99 2.11 0.99 0.99 3.04 1.12 3.08 3.02 4.22 11.45 3.30 21.98

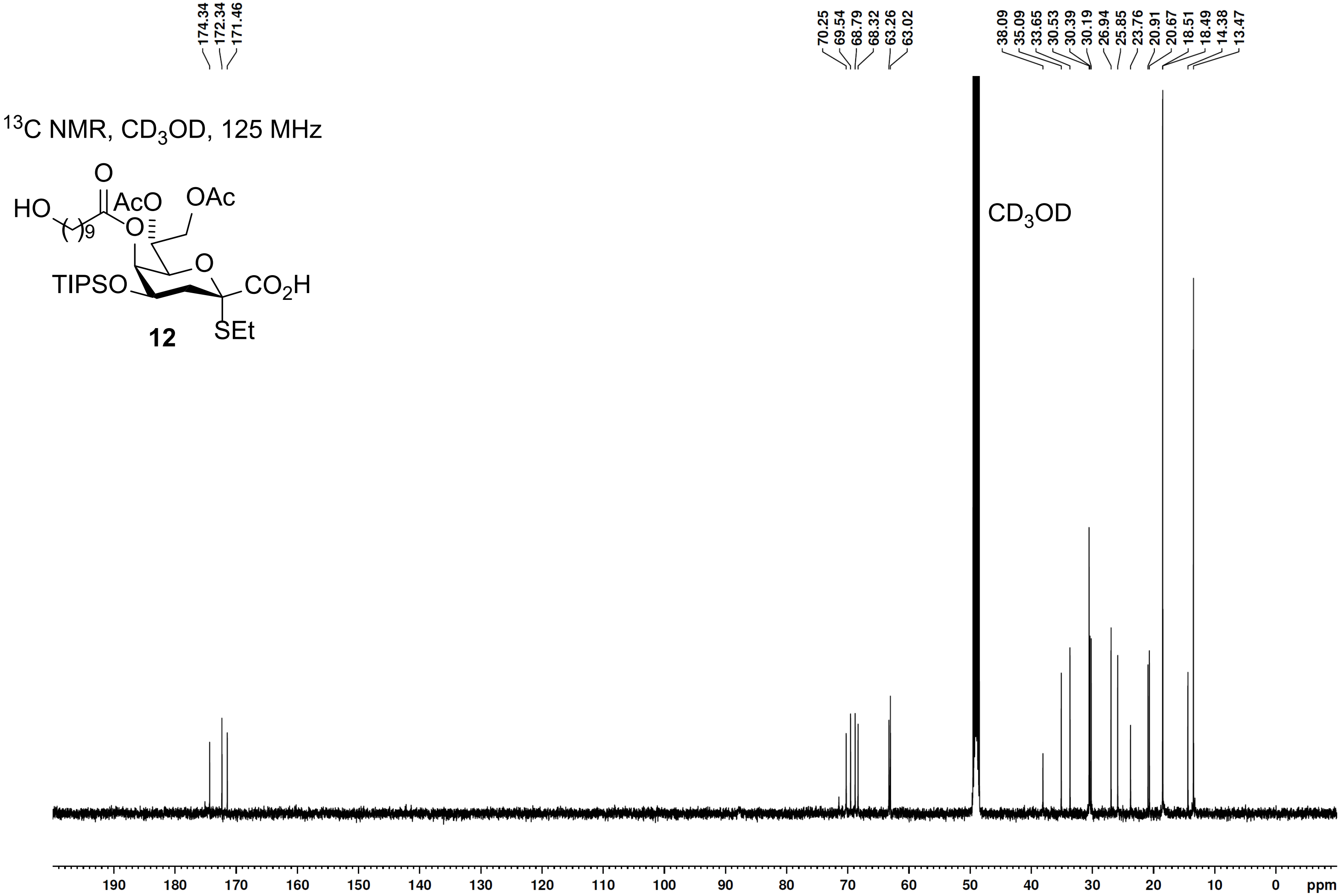

^{13}C NMR, CD_3OD, 125 MHz
12
CD3OD
174.34
172.34
171.46
70.25
69.54
68.79
68.32
63.26
63.02
38.09
35.09
33.65
30.53
30.39
30.19
26.94
25.85
23.76
20.91
20.67
18.51
18.49
14.38
13.47
190 180 170 160 150 140 130 120 110 100 90 80 70 60 50 40 30 20 10 0 ppm

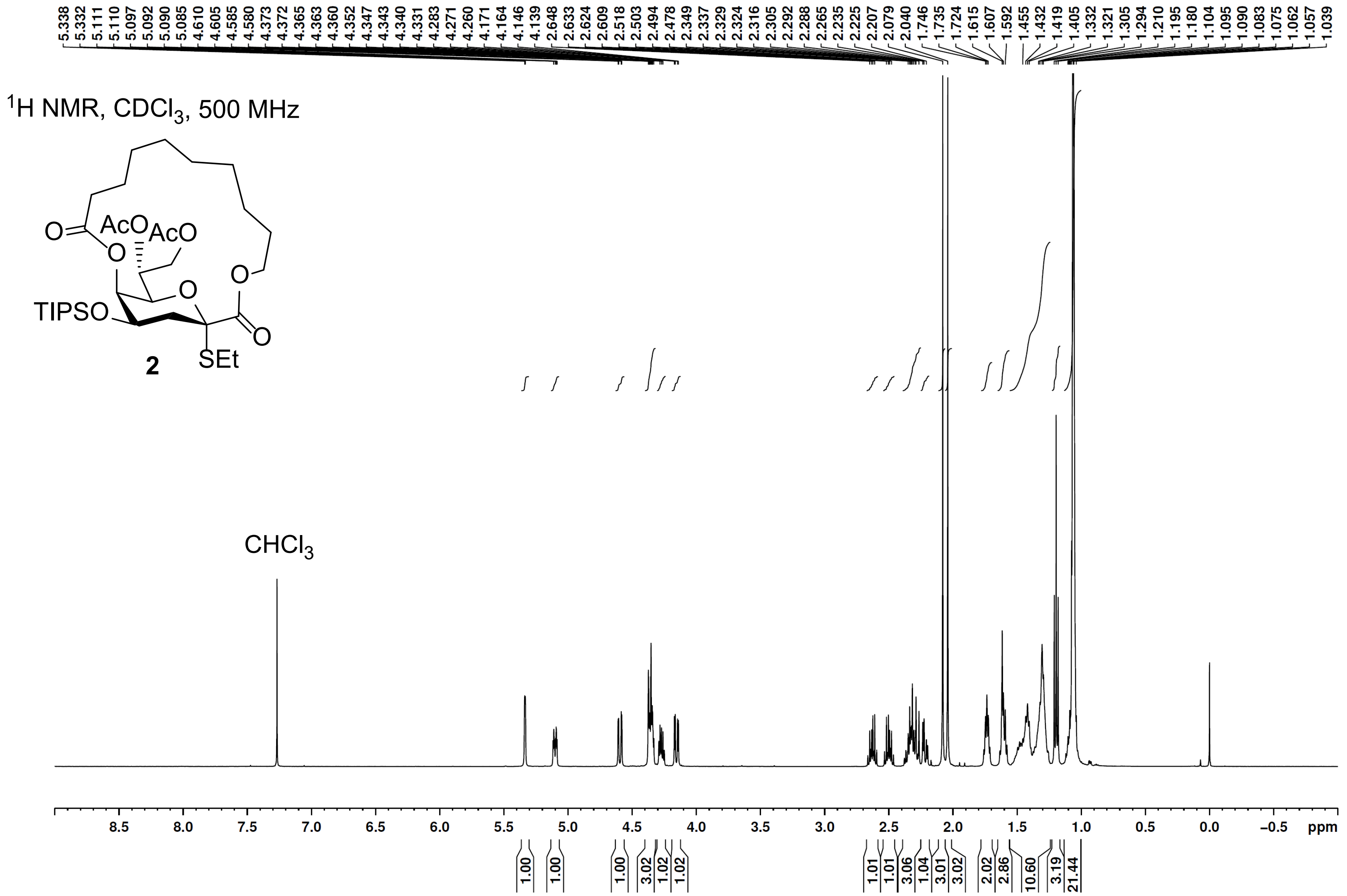

5.338 5.332 5.111 5.110 5.097 5.092 5.090 5.085 4.610 4.605 4.585 4.580 4.373 4.372 4.365 4.363 4.360 4.352 4.347 4.343 4.340 4.331 4.283 4.271 4.260 4.171 4.164 4.146 4.139 2.648 2.633 2.624 2.609 2.518 2.503 2.494 2.478 2.349 2.337 2.329 2.324 2.316 2.305 2.292 2.288 2.265 2.235 2.225 2.207 2.079 2.040 1.746 1.735 1.724 1.615 1.607 1.592 1.455 1.432 1.419 1.405 1.332 1.321 1.305 1.294 1.210 1.195 1.180 1.104 1.095 1.090 1.083 1.075 1.062 1.057 1.039
^{1}H NMR, $CDCl_3$, 500 MHz
AcO
AcO
O
O
O
O
TIPSO
O
SEt
2
$CHCl_3$
8.5 8.0 7.5 7.0 6.5 6.0 5.5 5.0 4.5 4.0 3.5 3.0 2.5 2.0 1.5 1.0 0.5 0.0 –0.5 ppm
1.00 1.00 1.00 3.02 1.02 1.02 1.01 1.01 3.06 1.04 3.01 3.02 2.02 2.86 10.60 3.19 21.44

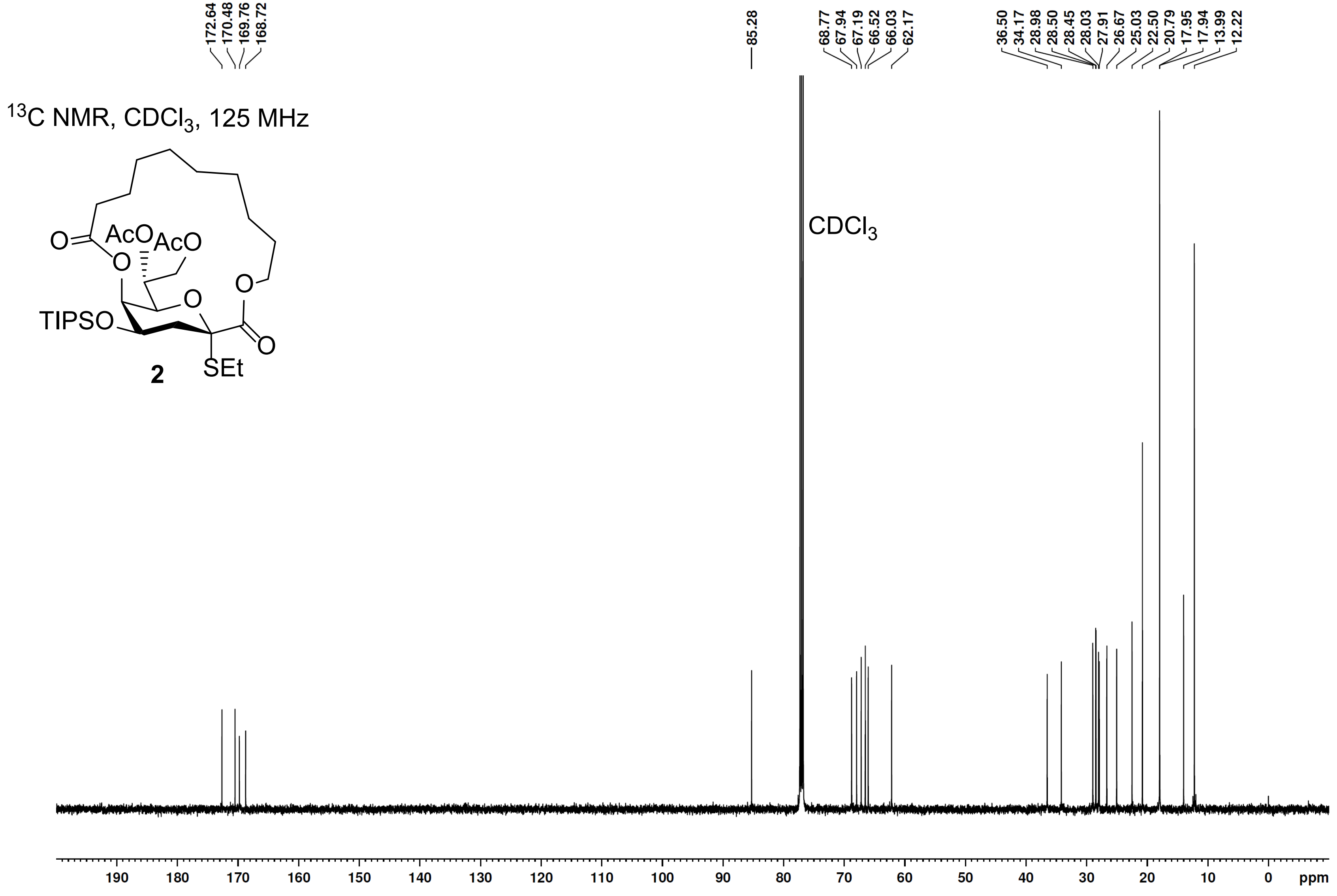

172.64
170.48
169.76
168.72
85.28
68.77
67.94
67.19
66.52
66.03
62.17
36.50
34.17
28.98
28.50
28.45
28.03
27.91
26.67
25.03
22.50
20.79
17.95
17.94
13.99
12.22
13C NMR, CDCl3, 125 MHz
AcO
AcO
O
O
O
O
O
TIPSO
SEt
2
CDCl3
190
180
170
160
150
140
130
120
110
100
90
80
70
60
50
40
30
20
10
0
ppm

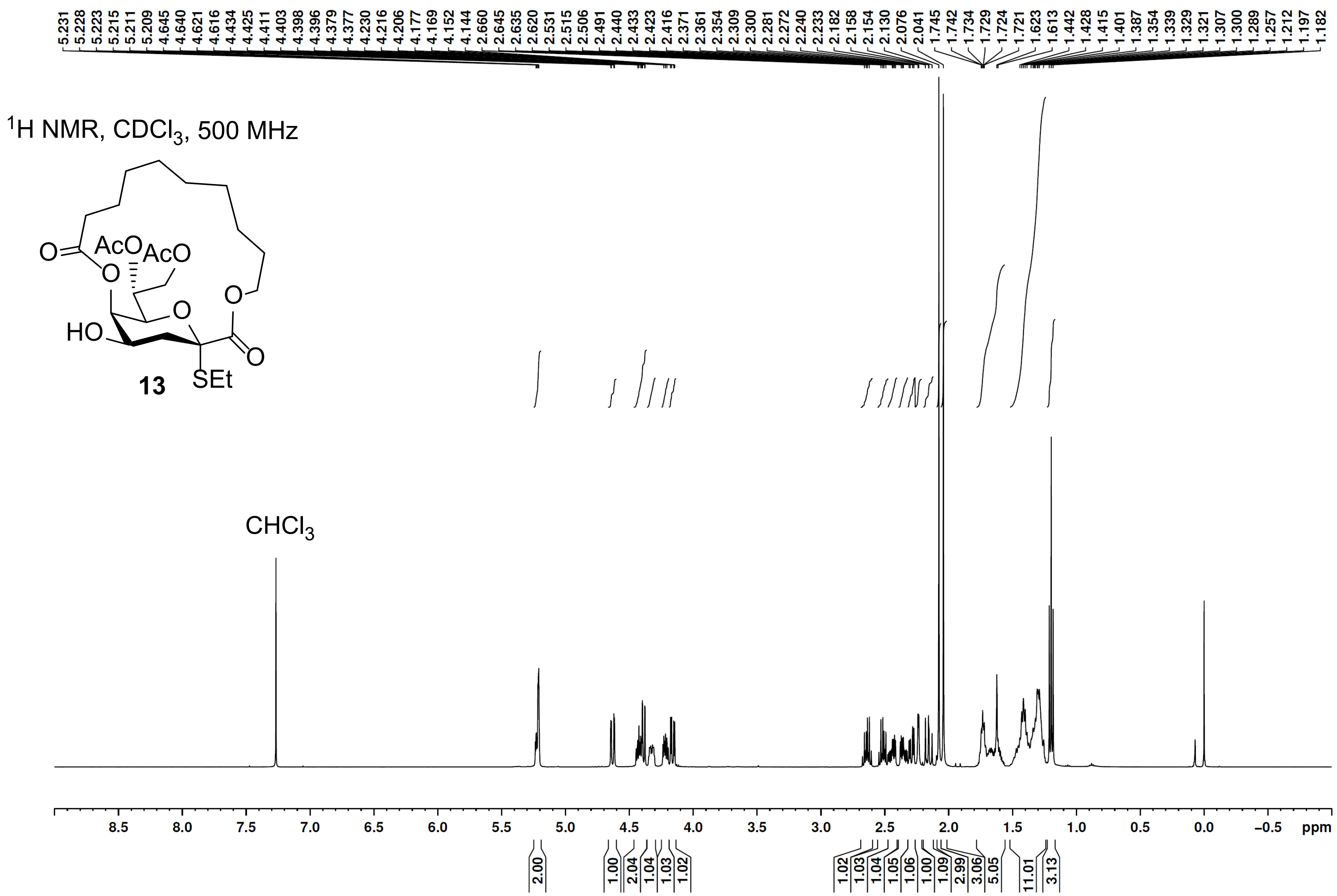

5.231 5.228 5.223 5.215 5.211 5.209 4.645 4.640 4.621 4.616 4.434 4.425 4.411 4.403 4.398 4.396 4.379 4.377 4.230 4.216 4.206 4.177 4.169 4.152 4.144 2.660 2.645 2.635 2.620 2.531 2.515 2.506 2.491 2.440 2.433 2.423 2.416 2.371 2.361 2.354 2.309 2.300 2.281 2.272 2.240 2.233 2.182 2.158 2.154 2.130 2.076 2.041 1.745 1.742 1.734 1.729 1.724 1.721 1.623 1.613 1.442 1.428 1.415 1.401 1.387 1.354 1.339 1.329 1.321 1.307 1.300 1.289 1.257 1.212 1.197 1.182
1H NMR, CDCl3, 500 MHz
AcO
AcO
O
O
O
O
HO
O
SEt
13
CHCl3
8.5 8.0 7.5 7.0 6.5 6.0 5.5 5.0 4.5 4.0 3.5 3.0 2.5 2.0 1.5 1.0 0.5 0.0 –0.5 ppm
2.00 1.00 2.04 1.04 1.03 1.02 1.02 1.03 1.04 1.05 1.06 1.00 1.09 2.99 3.06 5.05 11.01 3.13

^{13}C NMR, $CDCl_3$, 125 MHz

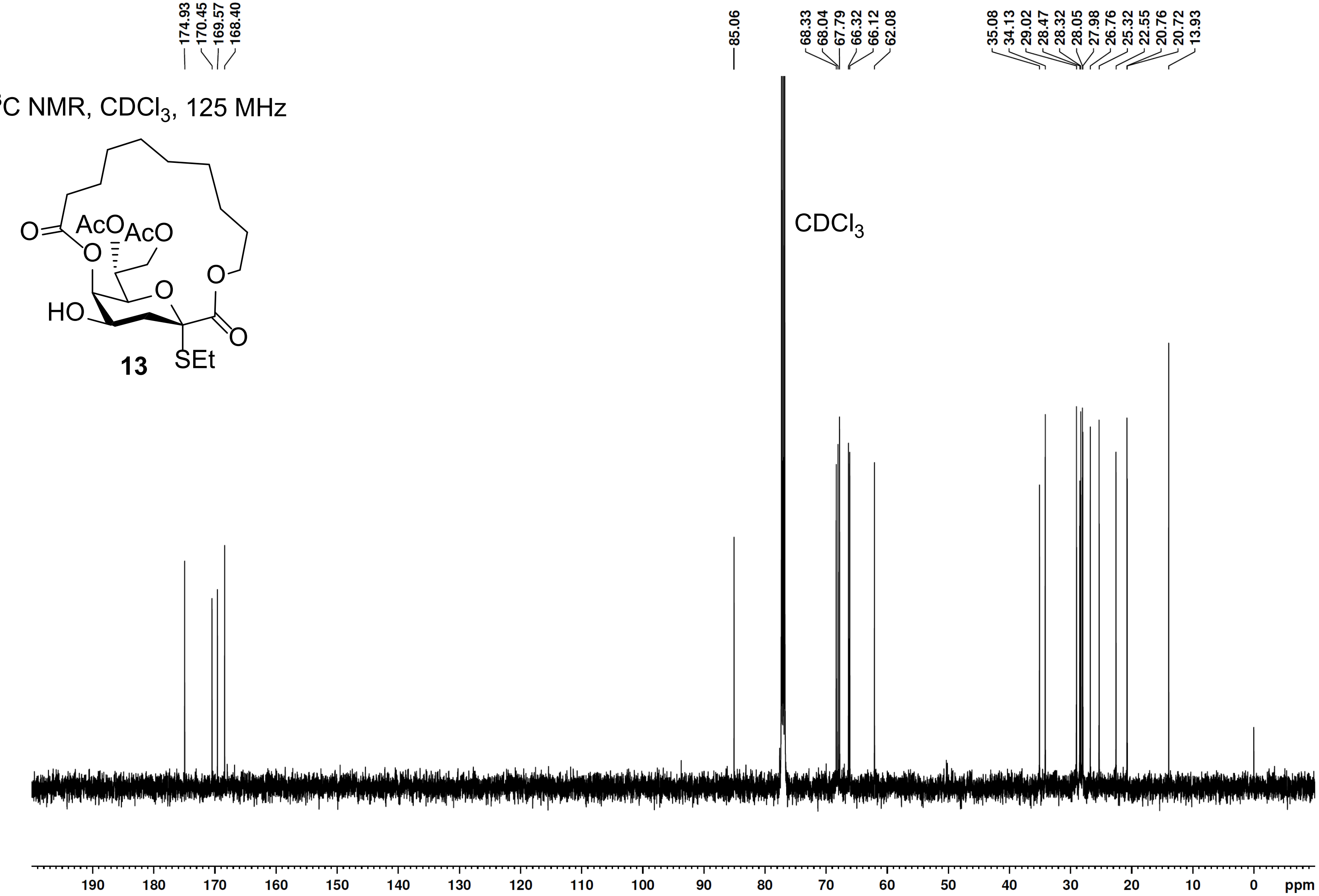

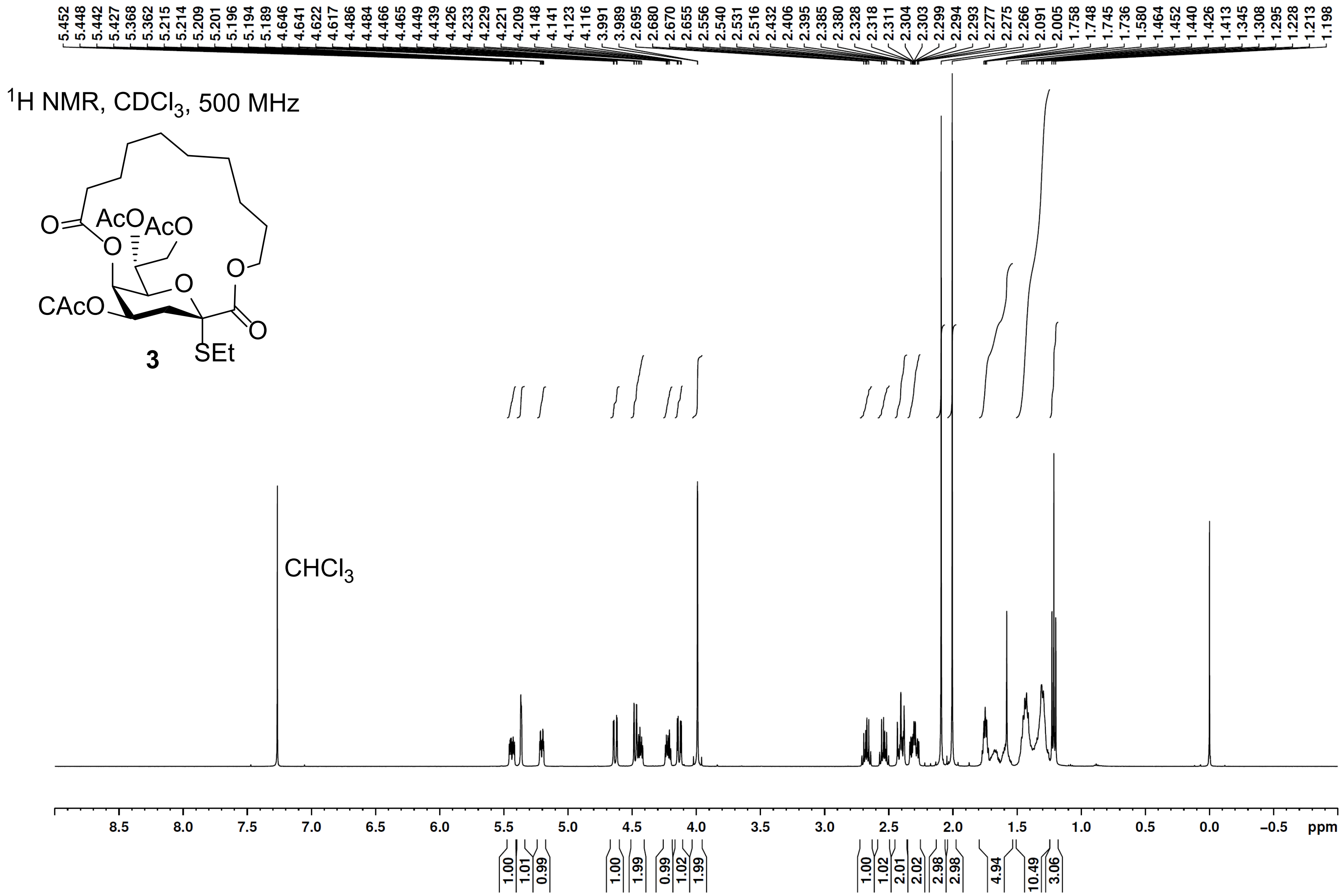

1H NMR, CDCl3, 500 MHz
O
AcO
AcO
O
O
CAcO
O
O
SEt
O
3
CHCl3
5.452 5.448 5.442 5.427 5.368 5.362 5.215 5.214 5.209 5.201 5.196 5.194 5.189 4.646 4.641 4.622 4.617 4.486 4.484 4.466 4.465 4.449 4.439 4.426 4.233 4.229 4.221 4.209 4.148 4.141 4.123 4.116 3.991 3.989 2.695 2.680 2.670 2.655 2.556 2.540 2.531 2.516 2.432 2.406 2.395 2.385 2.380 2.328 2.318 2.311 2.304 2.303 2.299 2.294 2.293 2.277 2.275 2.266 2.091 2.005 1.758 1.748 1.745 1.736 1.580 1.464 1.452 1.440 1.426 1.413 1.345 1.308 1.295 1.228 1.213 1.198
8.5 8.0 7.5 7.0 6.5 6.0 5.5 5.0 4.5 4.0 3.5 3.0 2.5 2.0 1.5 1.0 0.5 0.0 –0.5 ppm
1.00 1.01 0.99 1.00 1.99 0.99 1.02 1.99 1.00 1.02 2.01 2.02 2.98 2.98 4.94 10.49 3.06

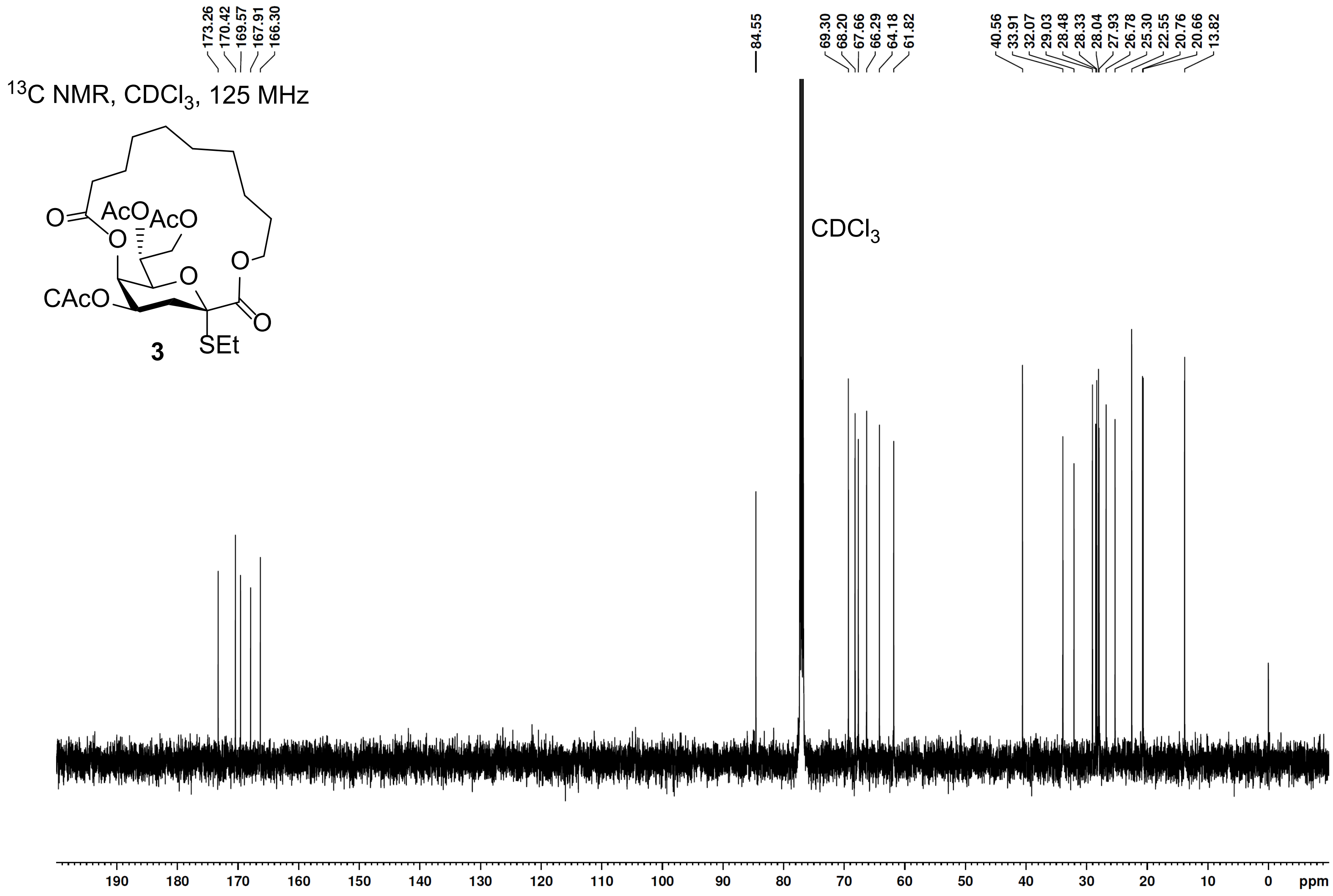
^{13}C NMR, $CDCl_3$, 125 MHz
173.26
170.42
169.57
167.91
166.30
84.55
69.30
68.20
67.66
66.29
64.18
61.82
40.56
33.91
32.07
29.03
28.48
28.33
28.04
27.93
26.78
25.30
22.55
20.76
20.66
13.82
AcO
AcO
CAcO
O
SEt
3
$CDCl_3$
190
180
170
160
150
140
130
120
110
100
90
80
70
60
50
40
30
20
10
0
ppm

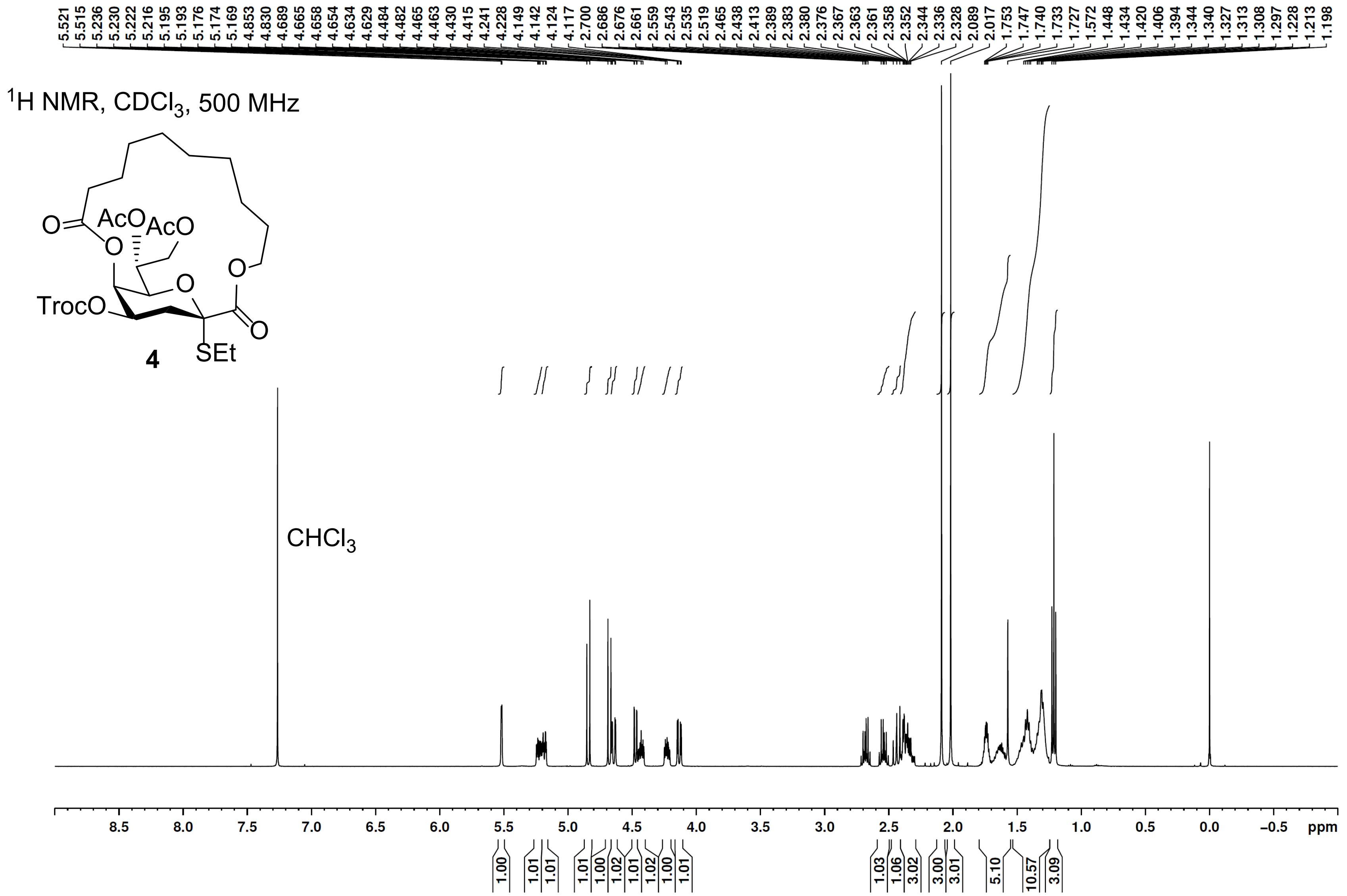

5.521 5.515 5.236 5.230 5.222 5.216 5.195 5.193 5.176 5.174 5.169 4.853 4.830 4.689 4.665 4.658 4.654 4.634 4.629 4.484 4.482 4.465 4.463 4.430 4.415 4.241 4.228 4.149 4.142 4.124 4.117 2.700 2.686 2.676 2.661 2.559 2.543 2.535 2.519 2.465 2.438 2.413 2.389 2.383 2.380 2.376 2.367 2.363 2.361 2.358 2.352 2.344 2.336 2.328 2.089 2.017 1.753 1.747 1.740 1.733 1.727 1.572 1.448 1.434 1.420 1.406 1.394 1.344 1.340 1.327 1.313 1.308 1.297 1.228 1.213 1.198
^{1}H NMR, $CDCl_3$, 500 MHz
O
AcO
AcO
O
O
O
TrocO
O
SEt
4
$CHCl_3$
8.5 8.0 7.5 7.0 6.5 6.0 5.5 5.0 4.5 4.0 3.5 3.0 2.5 2.0 1.5 1.0 0.5 0.0 –0.5 ppm
1.00 1.01 1.01 1.01 1.00 1.02 1.01 1.02 1.00 1.01 1.03 1.06 3.02 3.00 3.01 5.10 10.57 3.09

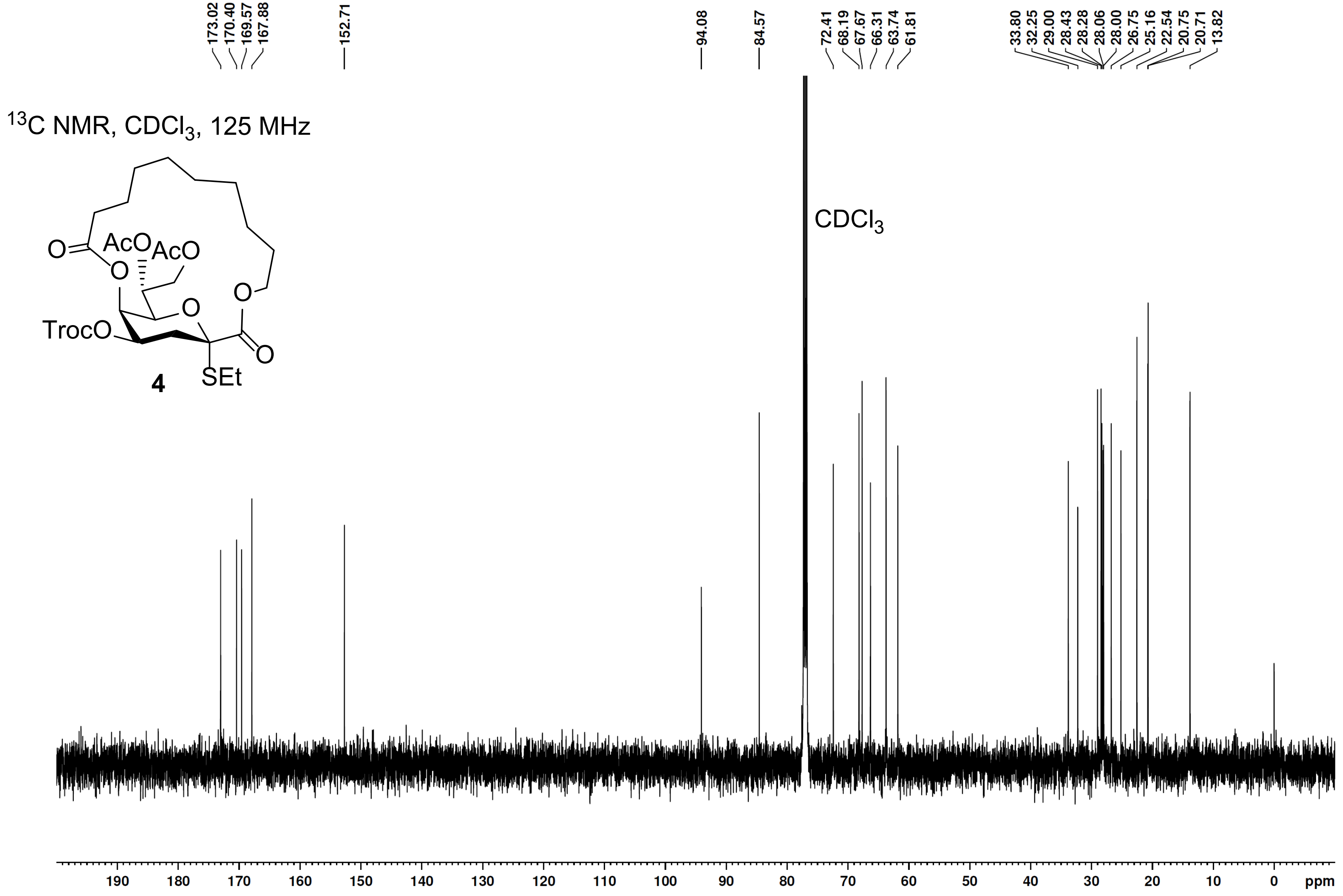

173.02
170.40
169.57
167.88
152.71
94.08
84.57
72.41
68.19
67.67
66.31
63.74
61.81
33.80
32.25
29.00
28.43
28.28
28.06
28.00
26.75
25.16
22.54
20.75
20.71
13.82
^{13}C NMR, $CDCl_3$, 125 MHz
O
AcO
AcO
O
O
O
TrocO
O
SEt
4
$CDCl_3$
190 180 170 160 150 140 130 120 110 100 90 80 70 60 50 40 30 20 10 0 ppm

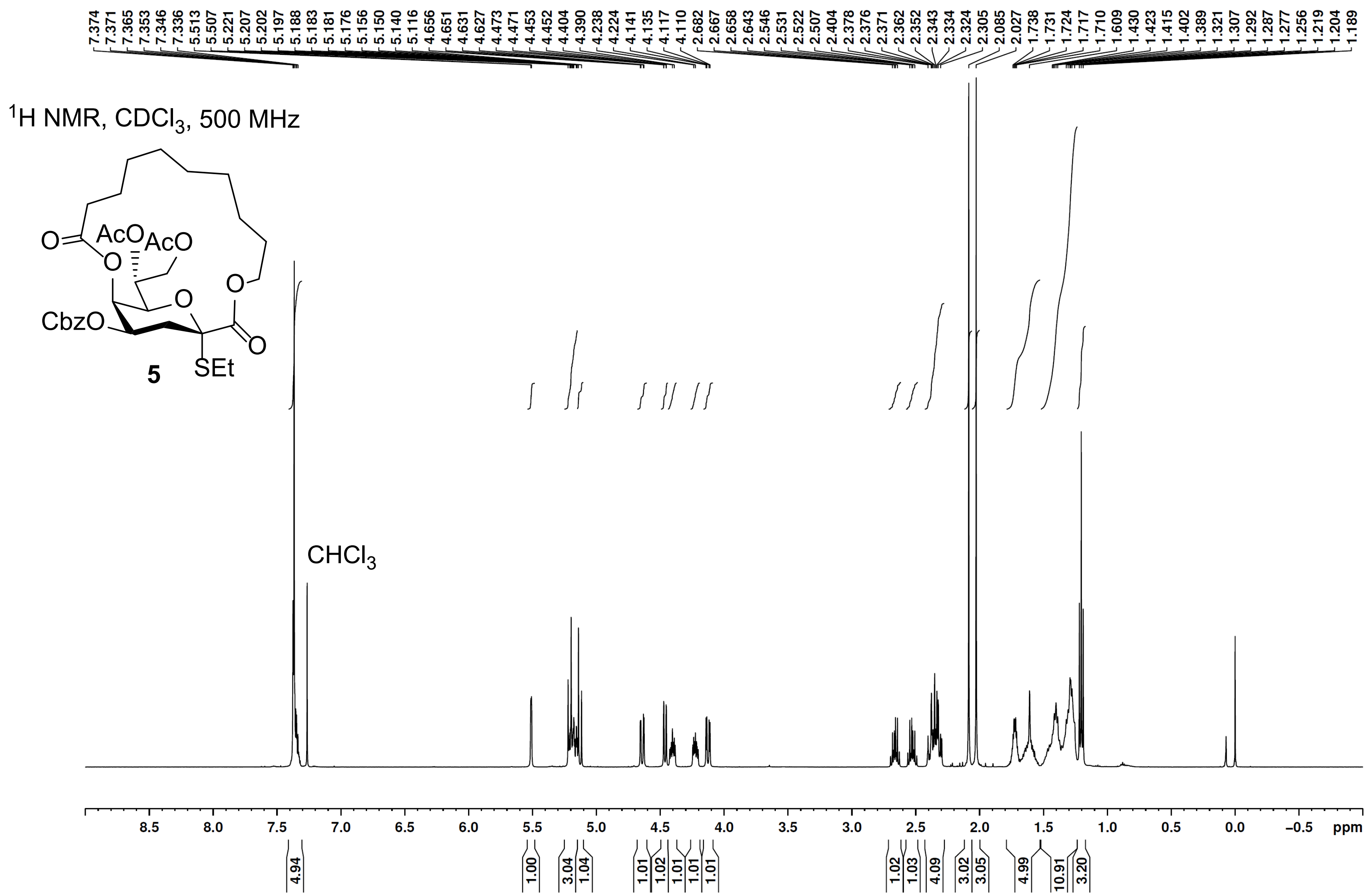

^{1}H NMR, $CDCl_3$, 500 MHz
5
CHCl$_3$
ppm

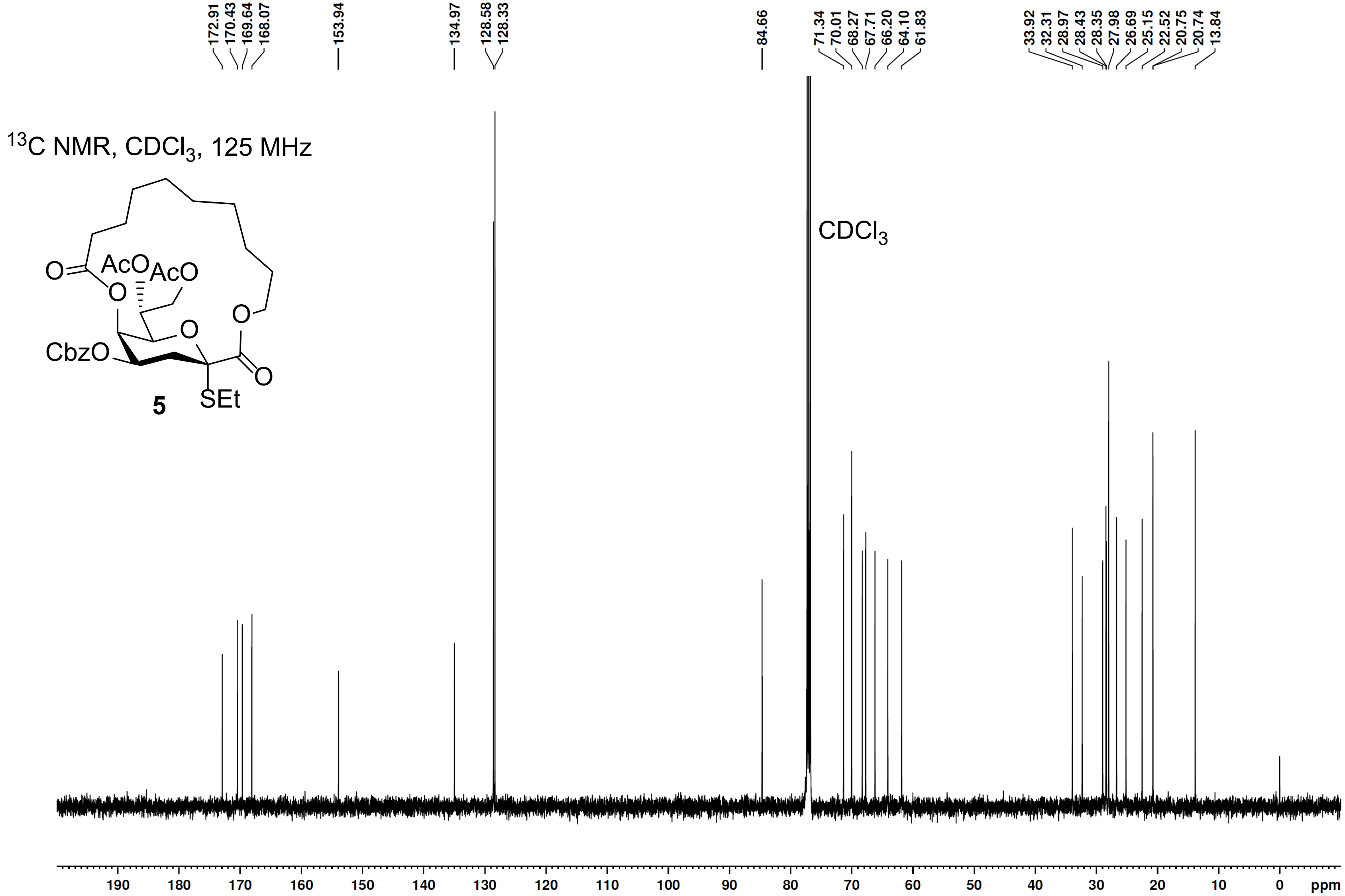
13C NMR, CDCl3, 125 MHz
172.91
170.43
169.64
168.07
153.94
134.97
128.58
128.33
84.66
71.34
70.01
68.27
67.71
66.20
64.10
61.83
33.92
32.31
28.97
28.43
28.35
27.98
26.69
25.15
22.52
20.75
20.74
13.84
CDCl3
AcO
AcO
CbzO
SEt
5
190 180 170 160 150 140 130 120 110 100 90 80 70 60 50 40 30 20 10 0 ppm

^1H NMR, $CDCl_3$, 500 MHz

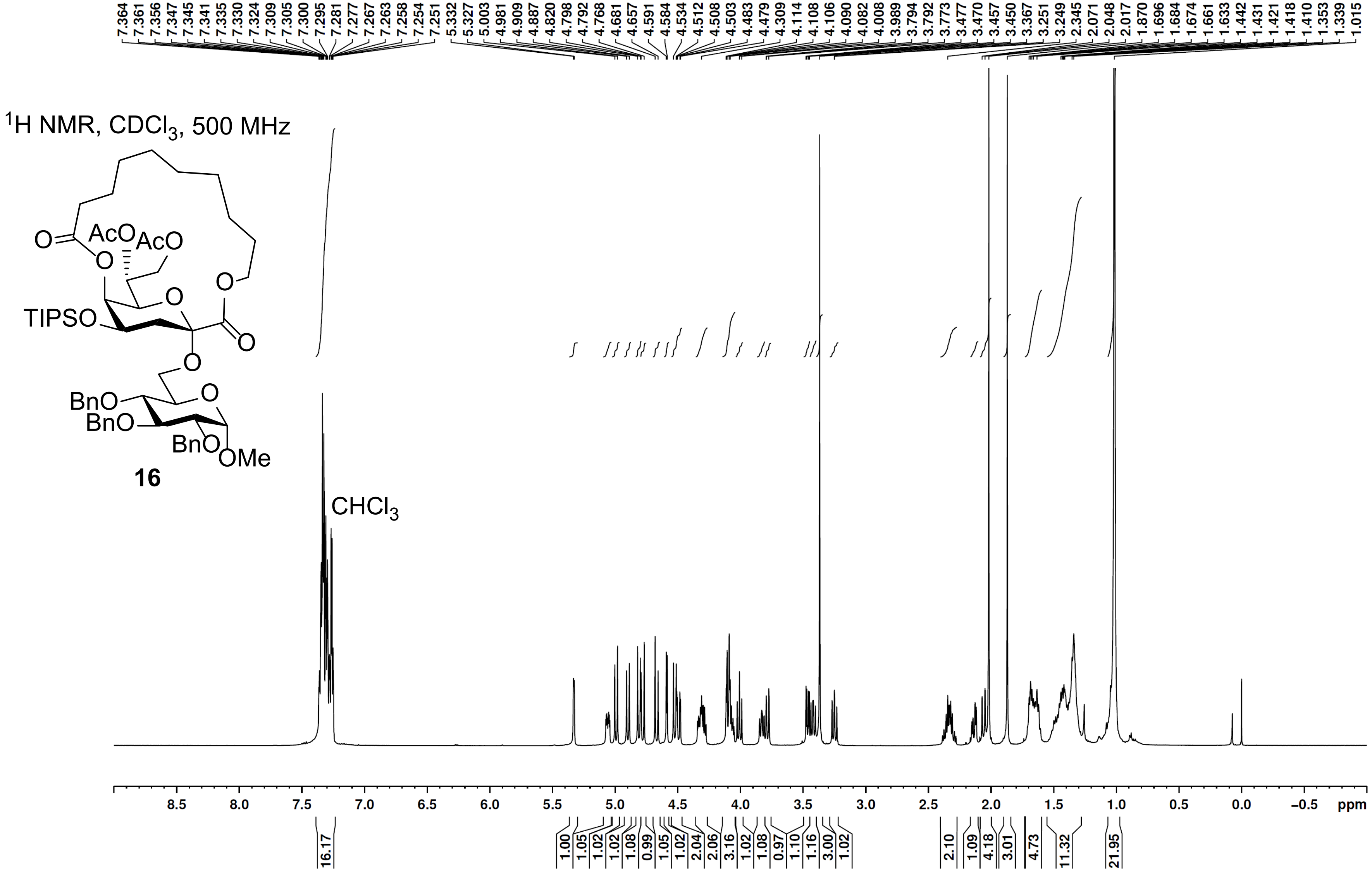

16

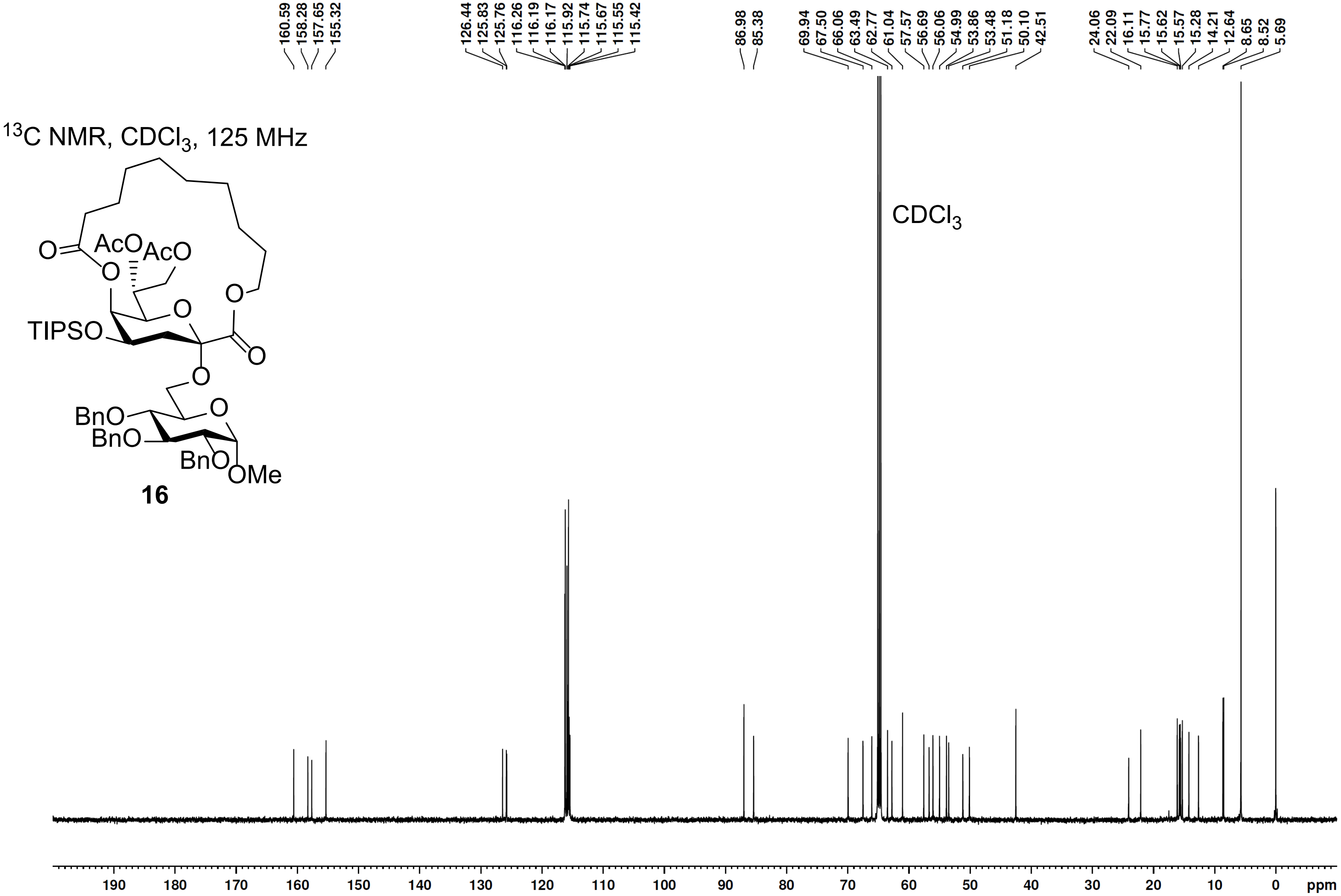

160.59
158.28
157.65
155.32
126.44
125.83
125.76
116.26
116.19
116.17
115.92
115.74
115.67
115.55
115.42
86.98
85.38
69.94
67.50
66.06
63.49
62.77
61.04
57.57
56.69
56.06
54.99
53.86
53.48
51.18
50.10
42.51
24.06
22.09
16.11
15.77
15.62
15.57
15.28
14.21
12.64
8.65
8.52
5.69
13C NMR, CDCl3, 125 MHz
AcO
AcO
O
O
O
O
O
TIPSO
O
BnO
BnO
BnO
O
OMe
16
CDCl3
190
180
170
160
150
140
130
120
110
100
90
80
70
60
50
40
30
20
10
0
ppm

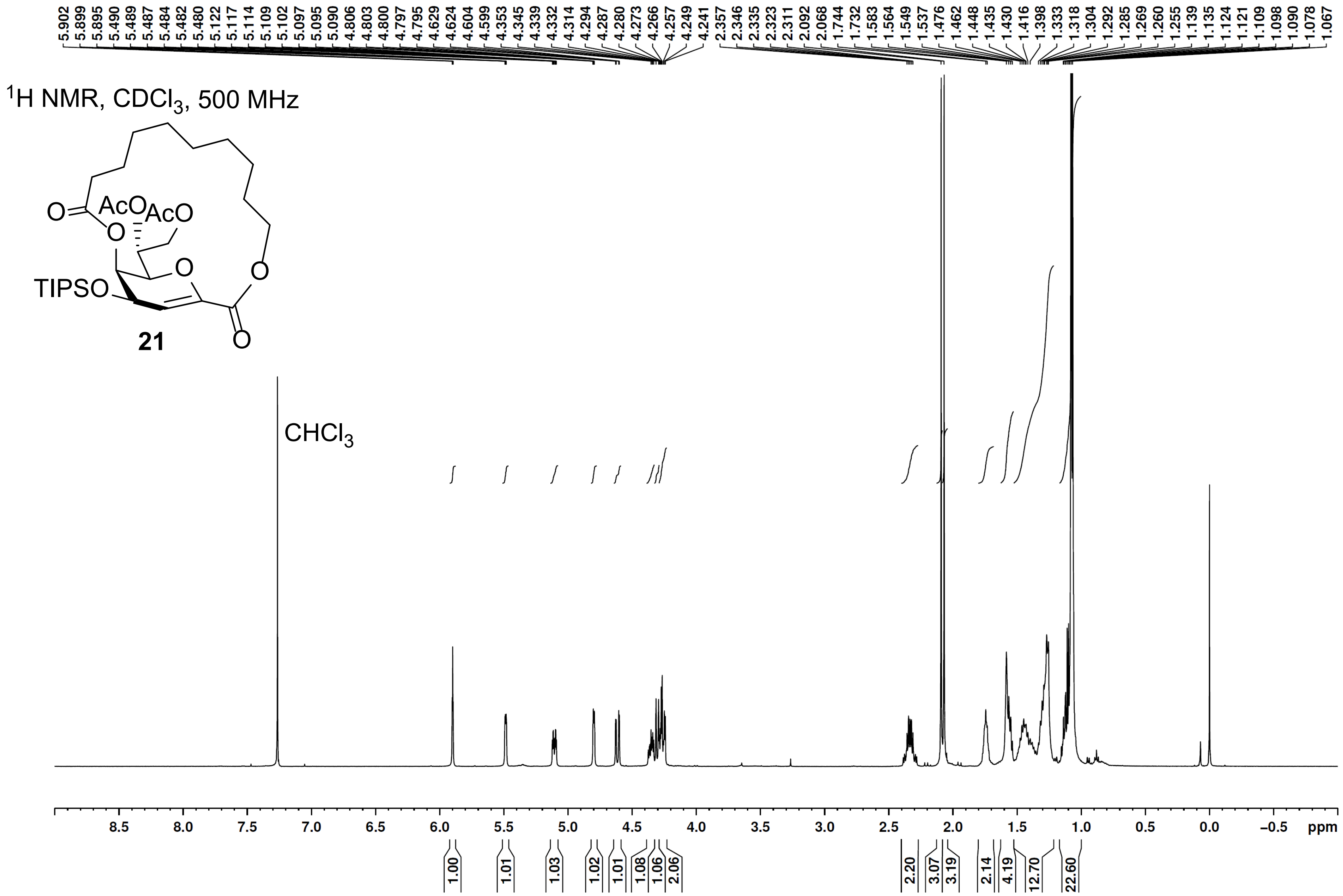
1H NMR, CDCl3, 500 MHz
O
AcO
AcO
O
O
TIPSO
O
O
21
CHCl3
5.902 5.899 5.895 5.490 5.489 5.487 5.484 5.482 5.480 5.122 5.117 5.114 5.109 5.102 5.097 5.095 5.090 4.806 4.803 4.800 4.797 4.795 4.629 4.624 4.604 4.599 4.353 4.345 4.339 4.332 4.314 4.294 4.287 4.280 4.273 4.266 4.257 4.249 4.241
2.357 2.346 2.335 2.323 2.311 2.092 2.068 1.744 1.732 1.583 1.564 1.549 1.537 1.476 1.462 1.448 1.435 1.430 1.416 1.398 1.333 1.318 1.304 1.292 1.285 1.269 1.260 1.255 1.139 1.135 1.124 1.121 1.109 1.098 1.090 1.078 1.067
8.5 8.0 7.5 7.0 6.5 6.0 5.5 5.0 4.5 4.0 3.5 3.0 2.5 2.0 1.5 1.0 0.5 0.0 –0.5 ppm
1.00 1.01 1.03 1.02 1.01 1.08 1.06 2.06 2.20 3.07 3.19 2.14 4.19 12.70 22.60

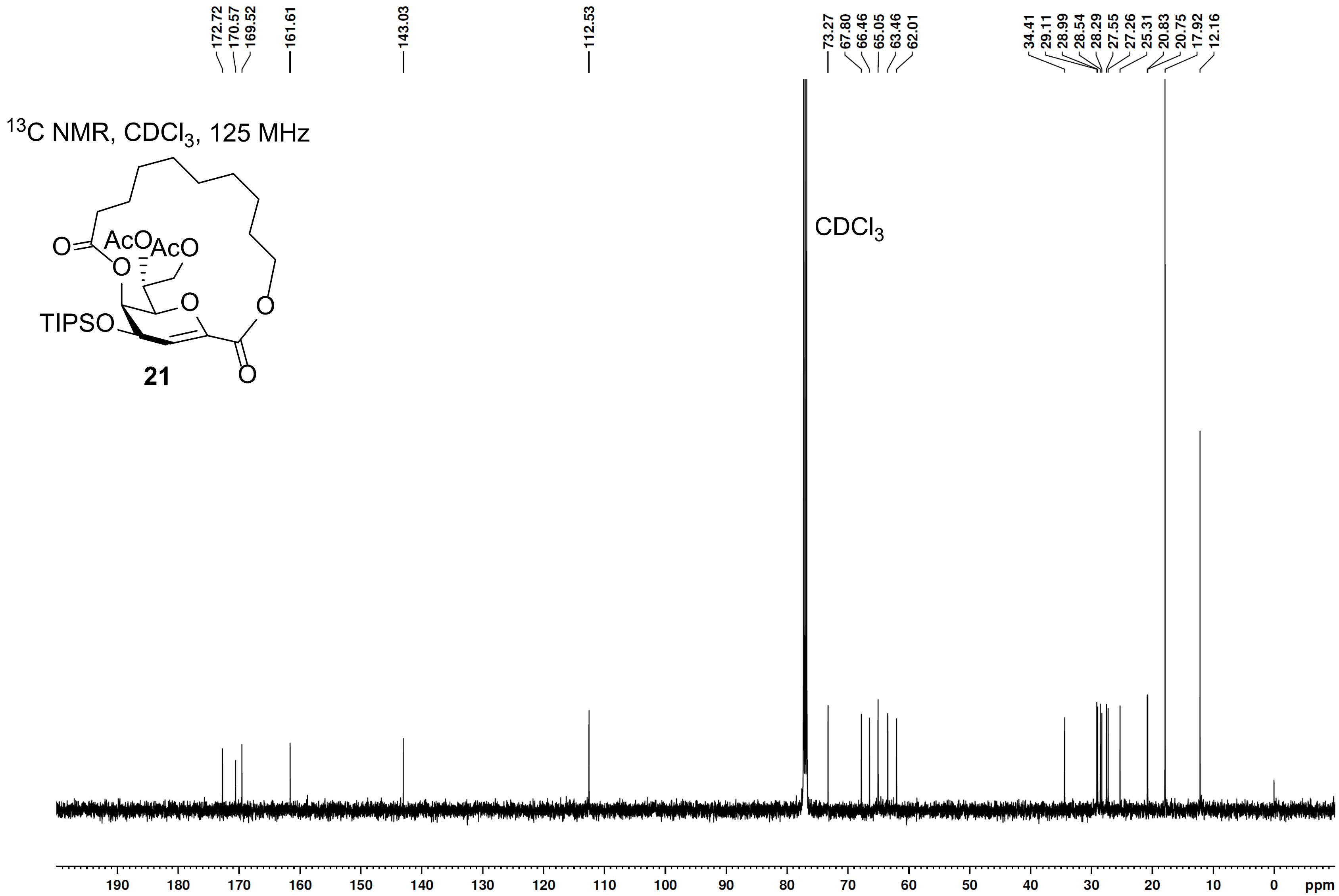

^{13}C NMR, $CDCl_3$, 125 MHz
21
172.72
170.57
169.52
161.61
143.03
112.53
73.27
67.80
66.46
65.05
63.46
62.01
34.41
29.11
28.99
28.54
28.29
27.55
27.26
25.31
20.83
20.75
17.92
12.16
$CDCl_3$
190 180 170 160 150 140 130 120 110 100 90 80 70 60 50 40 30 20 10 0 ppm

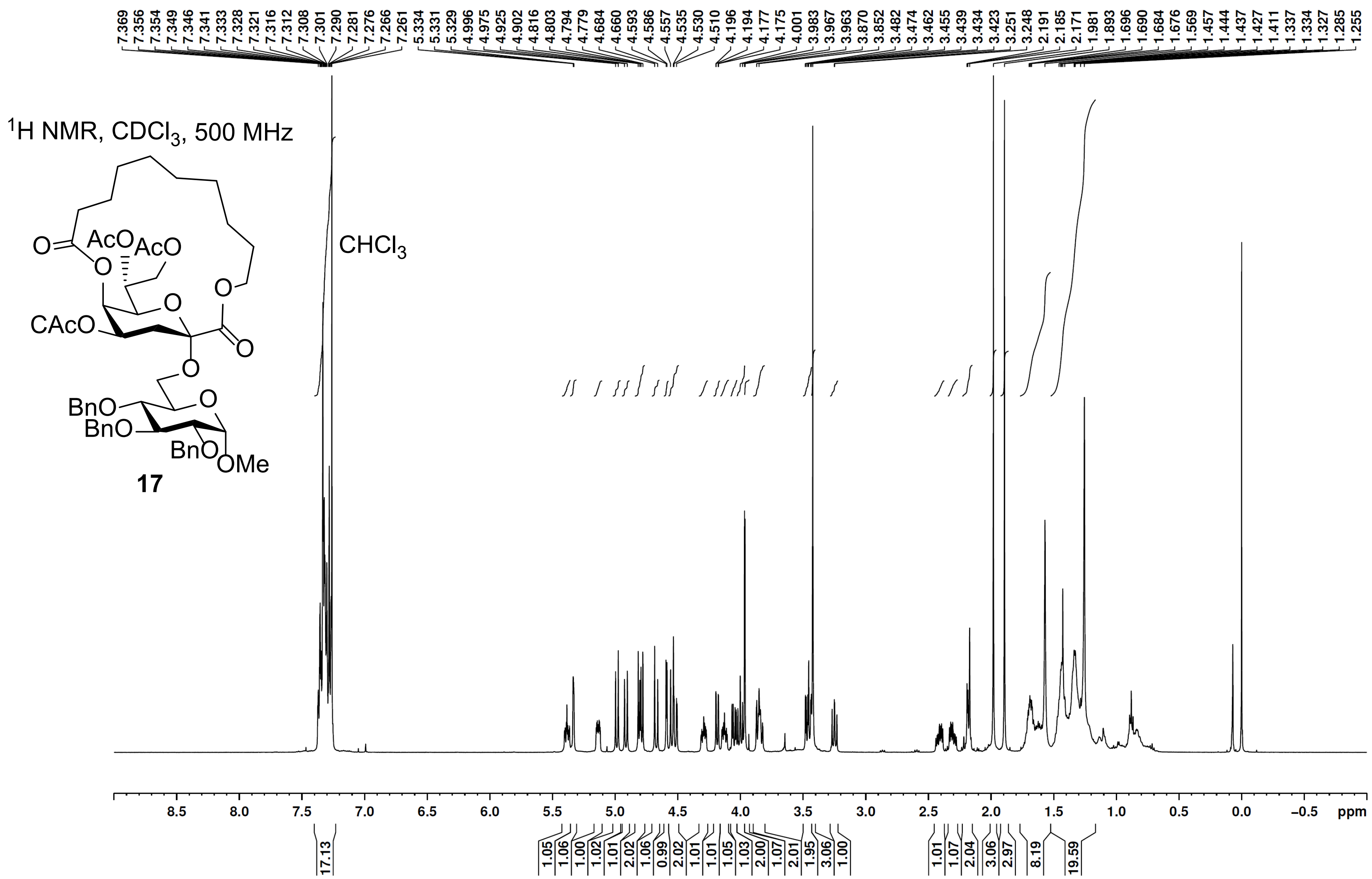

1H NMR, CDCl3, 500 MHz
CHCl3
17
ppm

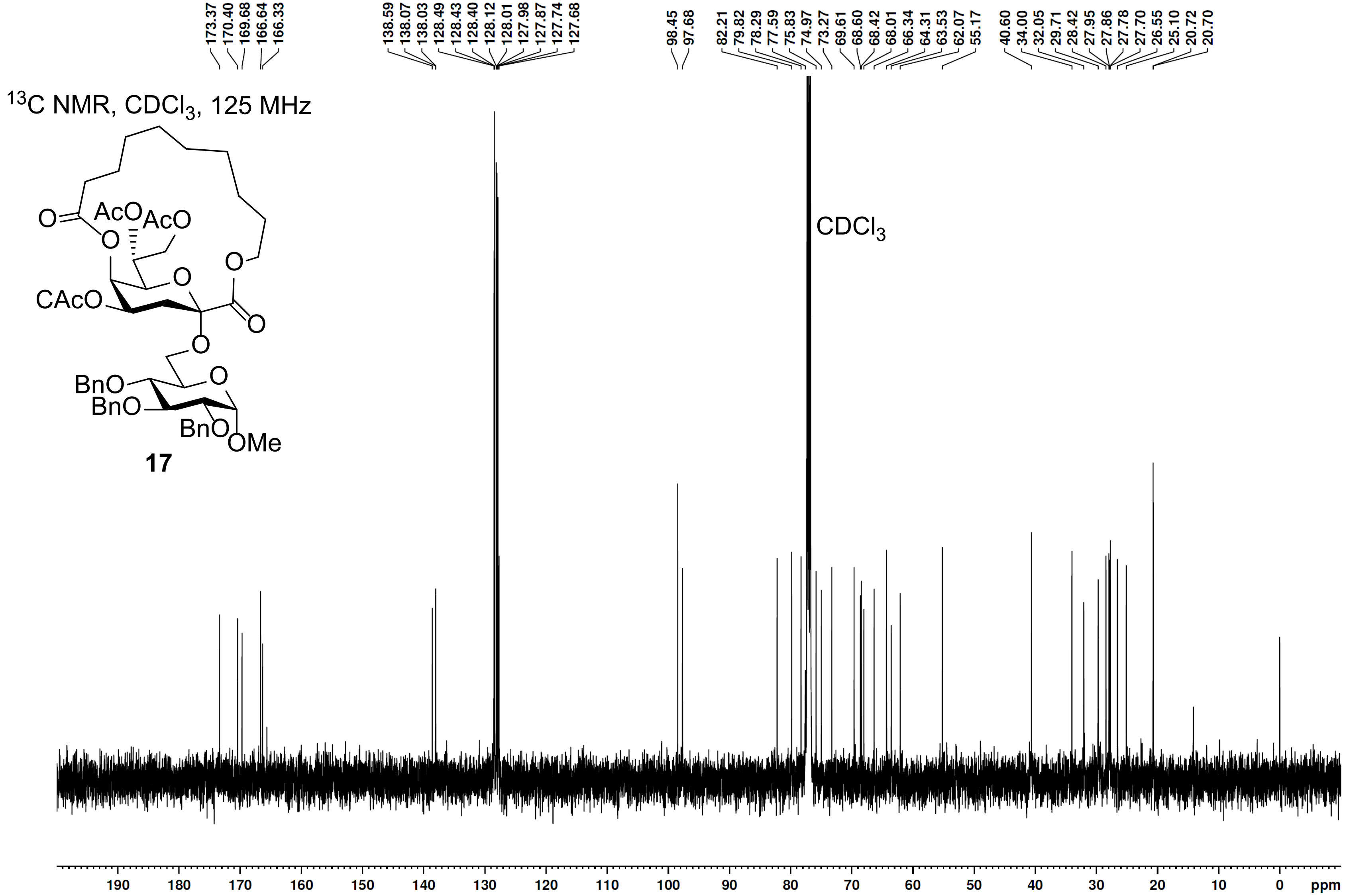

173.37
170.40
169.68
166.64
166.33
138.59
138.07
138.03
128.49
128.43
128.40
128.12
128.01
127.98
127.87
127.74
127.68
98.45
97.68
82.21
79.82
78.29
77.59
75.83
74.97
73.27
69.61
68.60
68.42
68.01
66.34
64.31
63.53
62.07
55.17
40.60
34.00
32.05
29.71
28.42
27.95
27.86
27.78
27.70
26.55
25.10
20.72
20.70
13C NMR, CDCl3, 125 MHz
O
AcO
AcO
O
O
O
CAcO
O
O
O
BnO
O
BnO
BnO
OMe
17
CDCl3
190
180
170
160
150
140
130
120
110
100
90
80
70
60
50
40
30
20
10
0
ppm

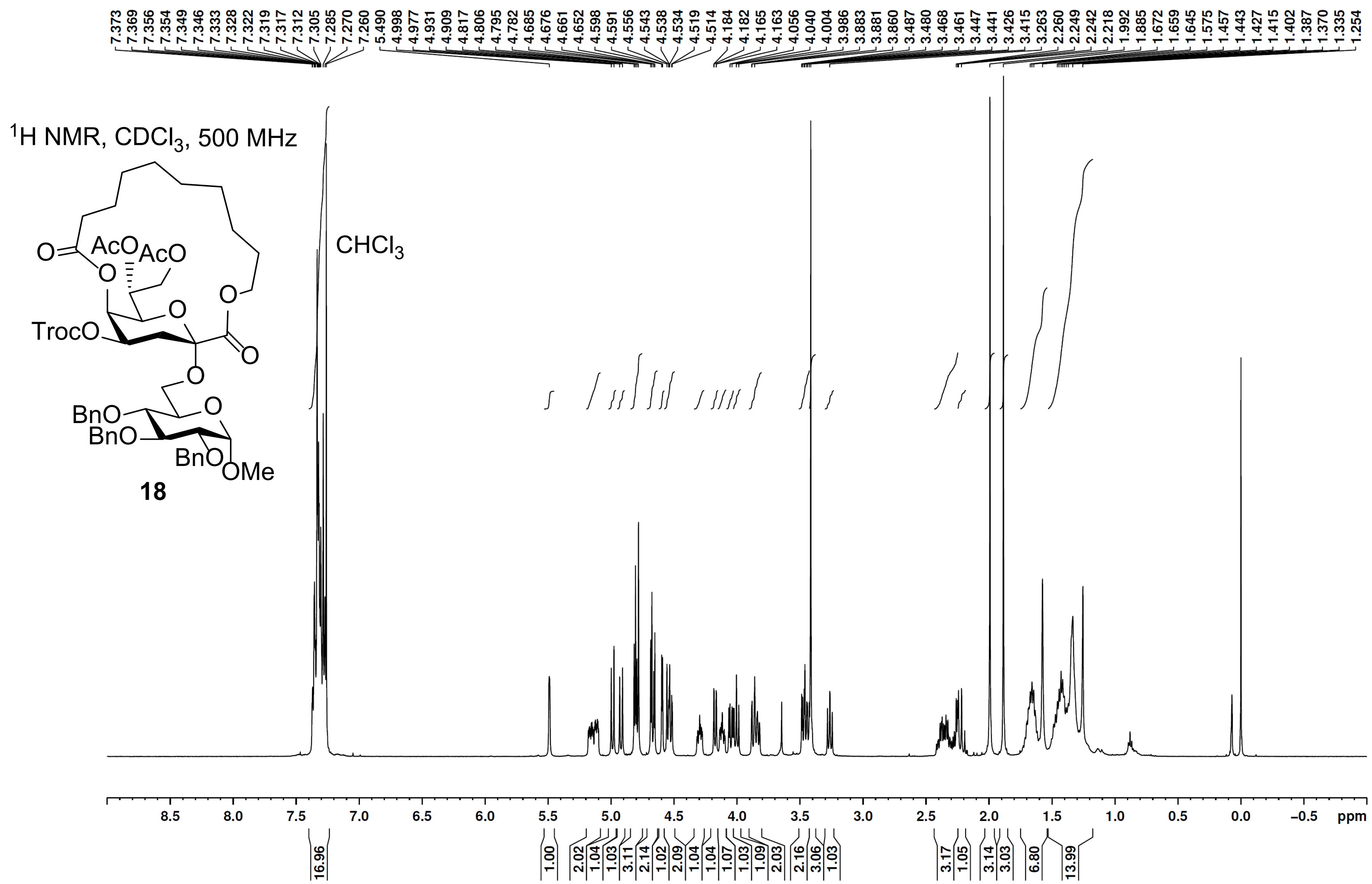

1H NMR, CDCl3, 500 MHz
CHCl3
AcO AcO
TrocO
BnO BnO BnO OMe
18
7.373 7.369 7.356 7.354 7.349 7.346 7.333 7.328 7.322 7.319 7.317 7.312 7.305 7.285 7.270 7.260 5.490 4.998 4.977 4.931 4.909 4.817 4.806 4.795 4.782 4.685 4.676 4.661 4.652 4.598 4.591 4.556 4.543 4.538 4.534 4.519 4.514 4.184 4.182 4.165 4.163 4.056 4.040 4.004 3.986 3.883 3.881 3.860 3.487 3.480 3.468 3.461 3.447 3.441 3.426 3.415 3.263 2.260 2.249 2.242 2.218 1.992 1.885 1.672 1.659 1.645 1.575 1.457 1.443 1.427 1.415 1.402 1.387 1.370 1.335 1.254
8.5 8.0 7.5 7.0 6.5 6.0 5.5 5.0 4.5 4.0 3.5 3.0 2.5 2.0 1.5 1.0 0.5 0.0 –0.5 ppm
16.96 1.00 2.02 1.04 1.03 3.11 2.14 1.02 2.09 1.04 1.04 1.07 1.03 1.09 2.03 2.16 3.06 1.03 3.17 1.05 3.14 3.03 6.80 13.99

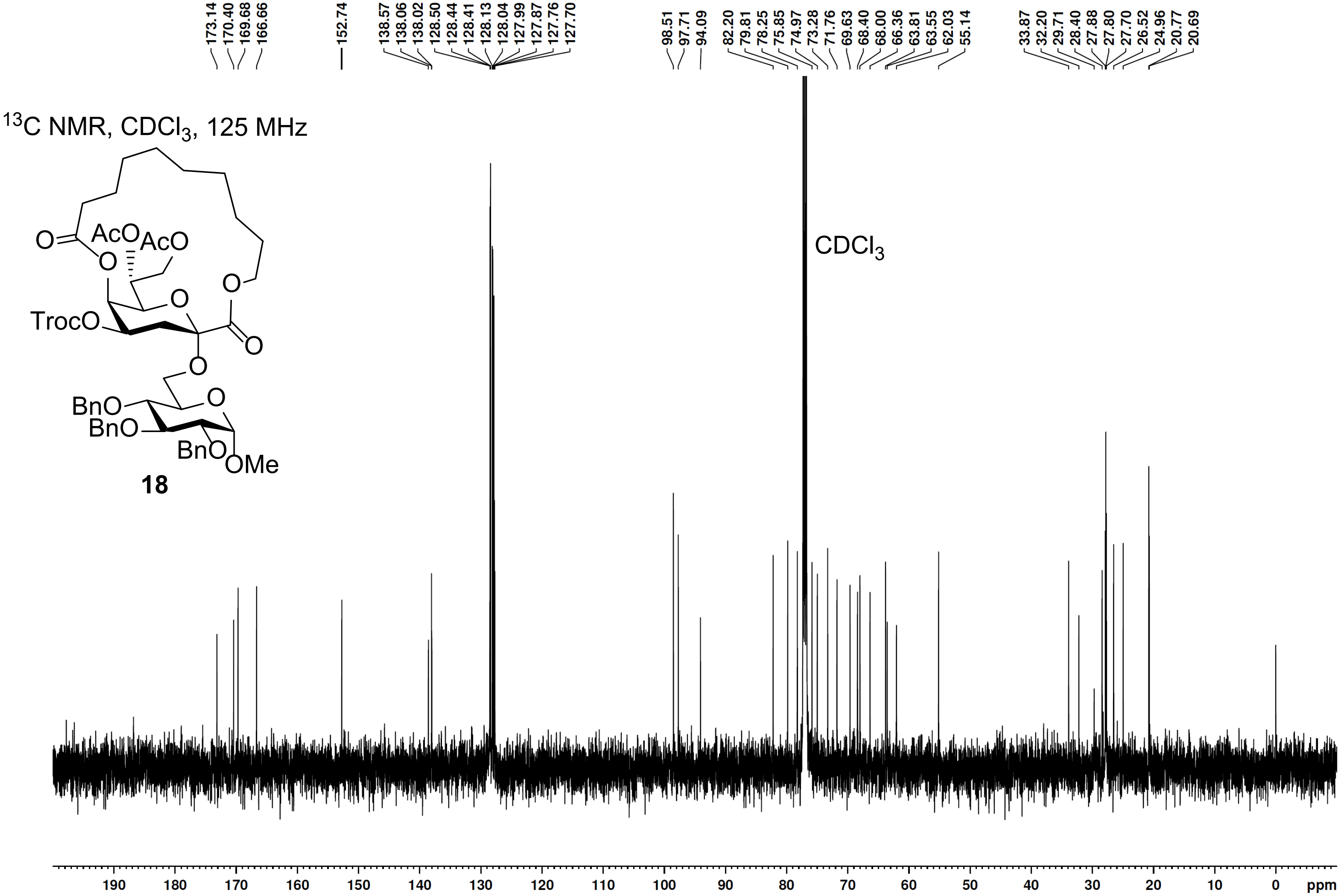

^{13}C NMR, CDCl$_3$, 125 MHz
173.14 170.40 169.68 166.66
152.74
138.57 138.06 138.02 128.50 128.44 128.41 128.13 128.04 127.99 127.87 127.76 127.70
98.51 97.71 94.09
82.20 79.81 78.25 75.85 74.97 73.28 71.76 69.63 68.40 68.00 66.36 63.81 63.55 62.03 55.14
33.87 32.20 29.71 28.40 27.88 27.80 27.70 26.52 24.96 20.77 20.69
O AcO AcO O O TrocO O O O BnO BnO O BnO OMe
18
CDCl$_3$
190 180 170 160 150 140 130 120 110 100 90 80 70 60 50 40 30 20 10 0 ppm

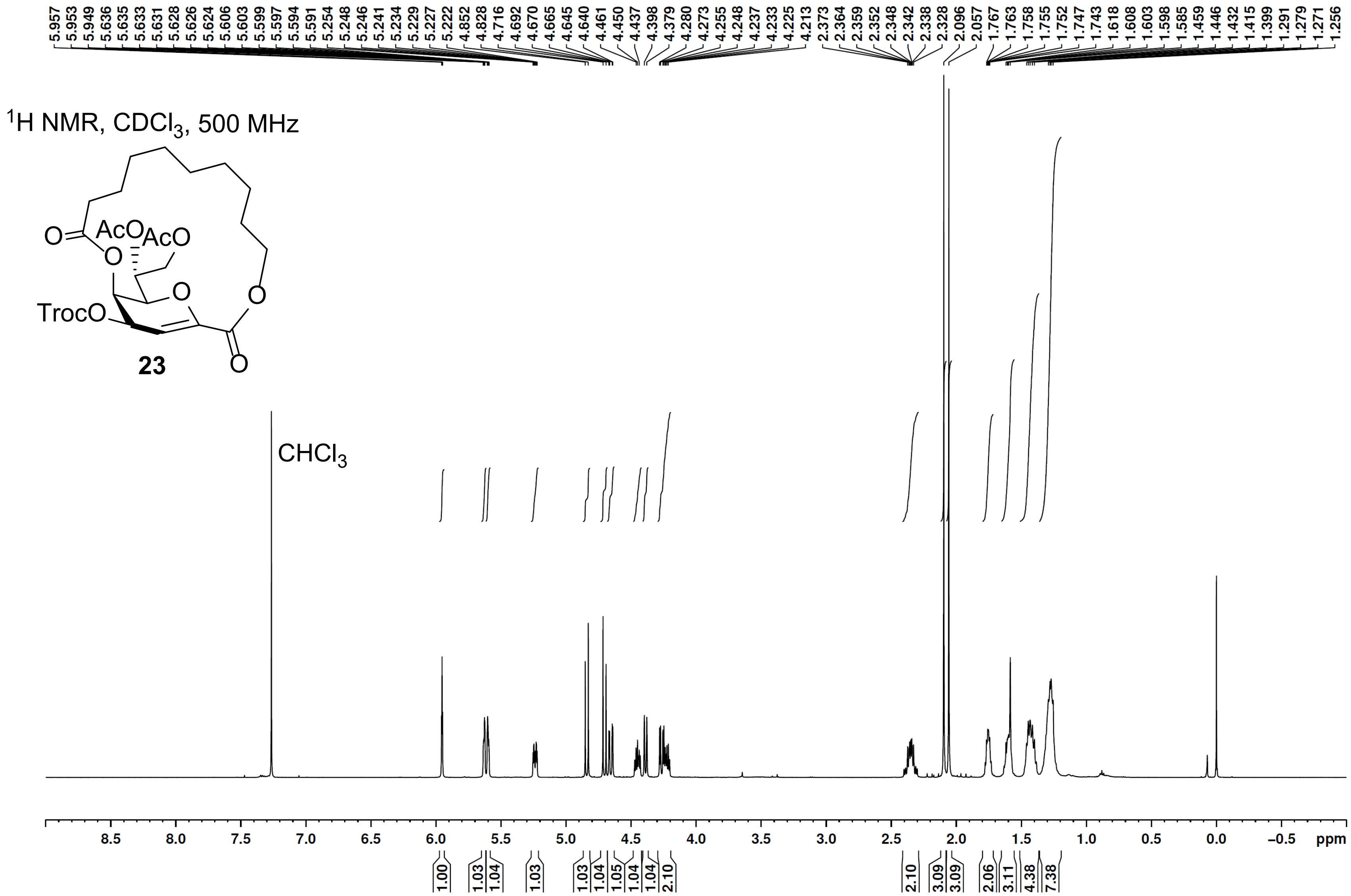

5.957 5.953 5.949 5.636 5.635 5.633 5.631 5.628 5.626 5.624 5.606 5.603 5.599 5.597 5.594 5.591 5.254 5.248 5.246 5.241 5.234 5.229 5.227 5.222 4.852 4.828 4.716 4.692 4.670 4.665 4.645 4.640 4.461 4.450 4.437 4.398 4.379 4.280 4.273 4.255 4.248 4.237 4.233 4.225 4.213 2.373 2.364 2.359 2.352 2.348 2.342 2.338 2.328 2.096 2.057 1.767 1.763 1.758 1.755 1.752 1.747 1.743 1.618 1.608 1.603 1.598 1.585 1.459 1.446 1.432 1.415 1.399 1.291 1.279 1.271 1.256
1H NMR, CDCl3, 500 MHz
AcO
AcO
O
TrocO
23
CHCl3
8.5 8.0 7.5 7.0 6.5 6.0 5.5 5.0 4.5 4.0 3.5 3.0 2.5 2.0 1.5 1.0 0.5 0.0 –0.5 ppm
1.00 1.03 1.04 1.03 1.03 1.04 1.05 1.04 1.04 2.10 2.10 3.09 3.09 2.06 3.11 4.38 7.38

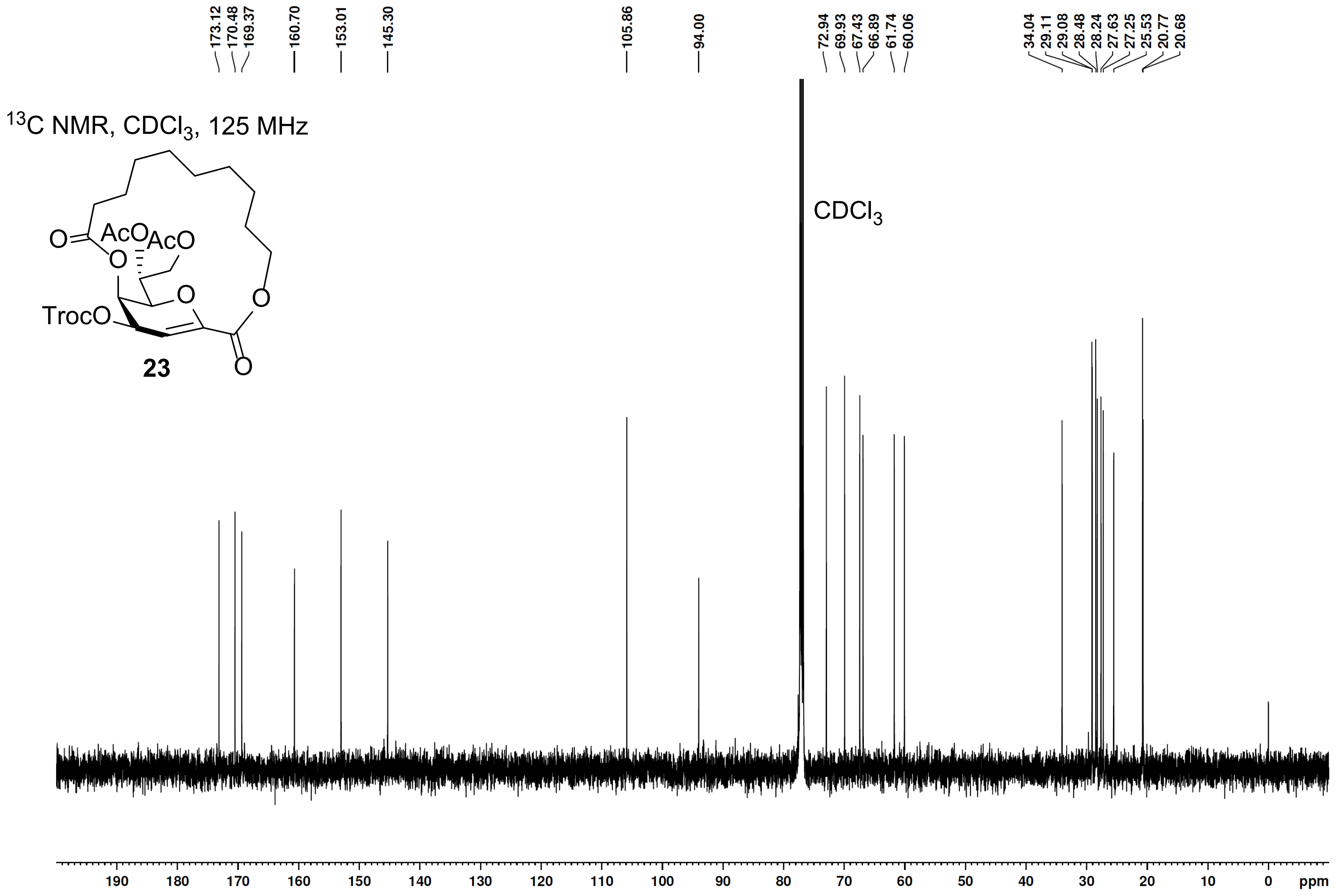

173.12
170.48
169.37
160.70
153.01
145.30
105.86
94.00
72.94
69.93
67.43
66.89
61.74
60.06
34.04
29.11
29.08
28.48
28.24
27.63
27.25
25.53
20.77
20.68
13C NMR, CDCl3, 125 MHz
CDCl3
O
AcO
AcO
O
O
TrocO
O
O
23
190
180
170
160
150
140
130
120
110
100
90
80
70
60
50
40
30
20
10
0
ppm

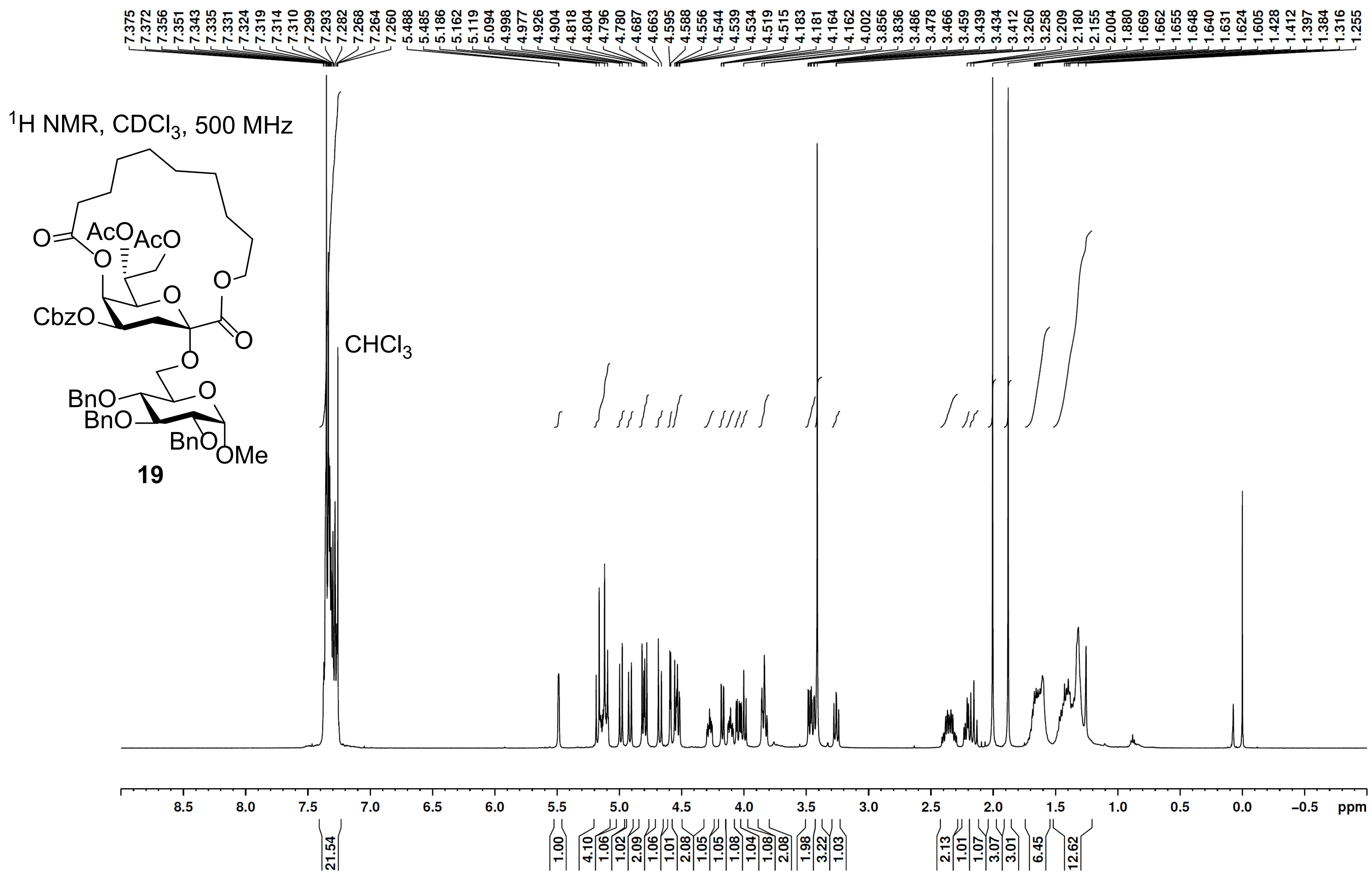
1H NMR, CDCl3, 500 MHz
AcO
AcO
O
CbzO
O
BnO
BnO
BnO
OMe
19
CHCl3
7.375 7.372 7.356 7.351 7.343 7.335 7.331 7.324 7.319 7.314 7.310 7.299 7.293 7.282 7.268 7.264 7.260 5.488 5.485 5.186 5.162 5.119 5.094 4.998 4.977 4.926 4.904 4.818 4.804 4.796 4.780 4.687 4.663 4.595 4.588 4.556 4.544 4.539 4.534 4.519 4.515 4.183 4.181 4.164 4.162 4.002 3.856 3.836 3.486 3.478 3.466 3.459 3.439 3.434 3.412 3.260 3.258 2.209 2.180 2.155 2.004 1.880 1.669 1.662 1.655 1.648 1.640 1.631 1.624 1.605 1.428 1.412 1.397 1.384 1.316 1.255
8.5 8.0 7.5 7.0 6.5 6.0 5.5 5.0 4.5 4.0 3.5 3.0 2.5 2.0 1.5 1.0 0.5 0.0 –0.5 ppm
21.54 1.00 4.10 1.06 1.02 2.09 1.06 1.01 2.08 1.05 1.05 1.08 1.04 1.08 2.08 1.98 3.22 1.03 2.13 1.01 1.07 3.07 3.01 6.45 12.62

^{13}C NMR, $CDCl_3$, 125 MHz

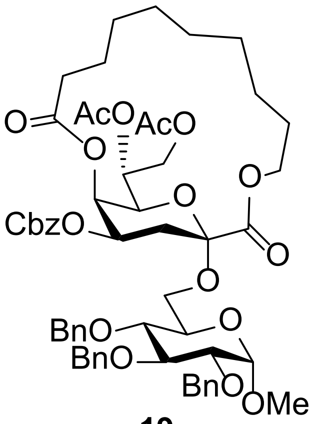

19

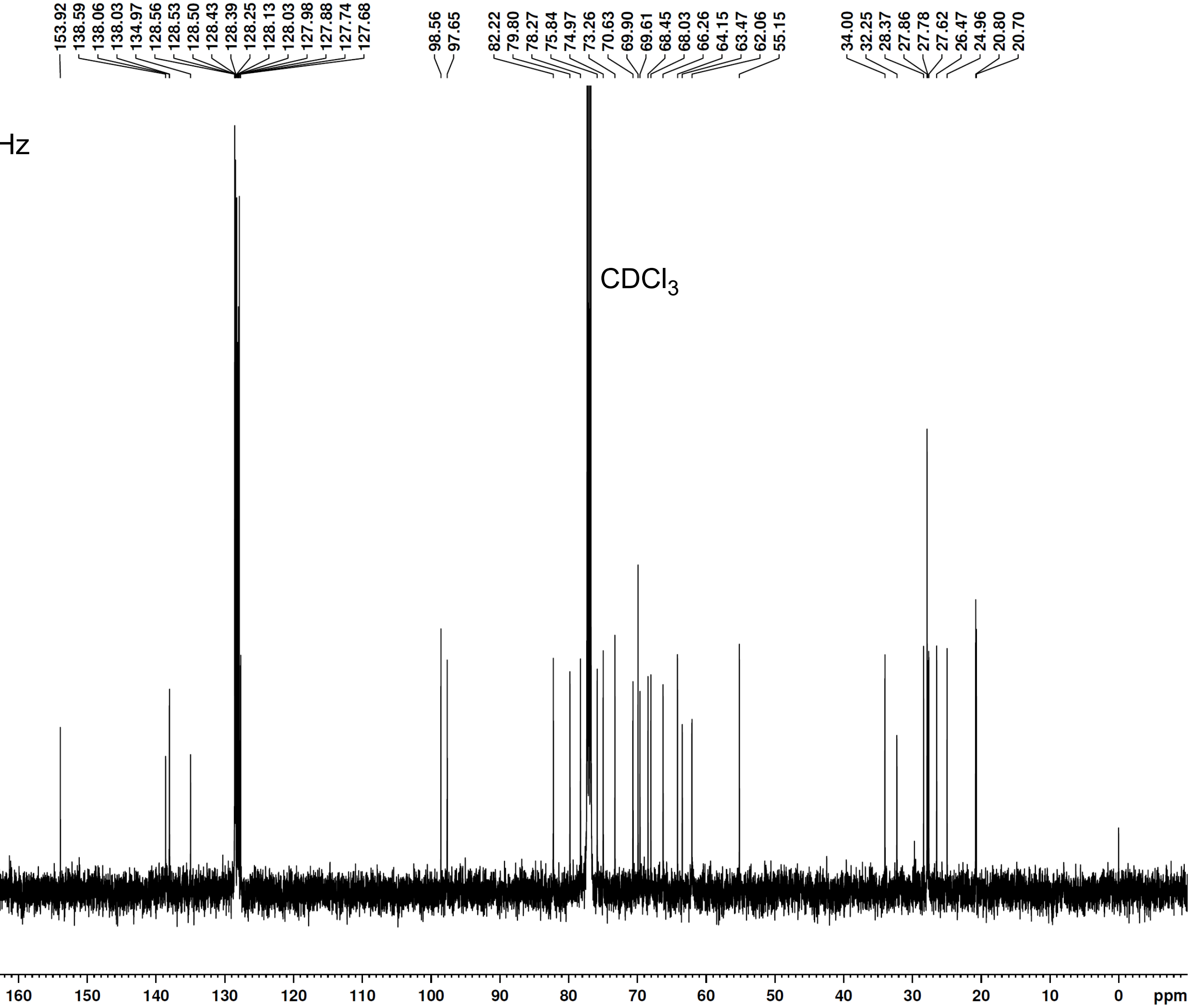

^{1}H NMR, $CDCl_3$, 500 MHz

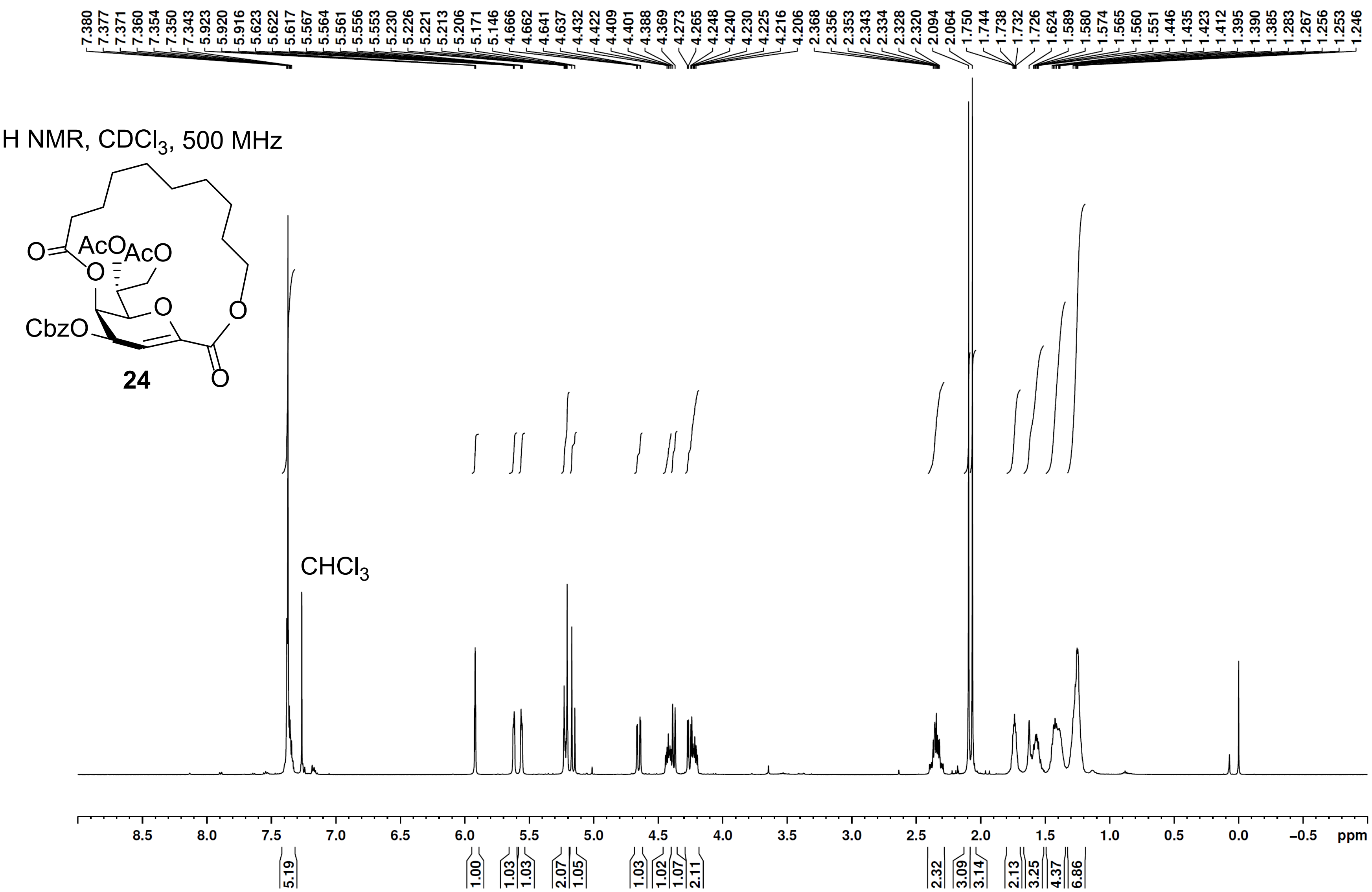

24

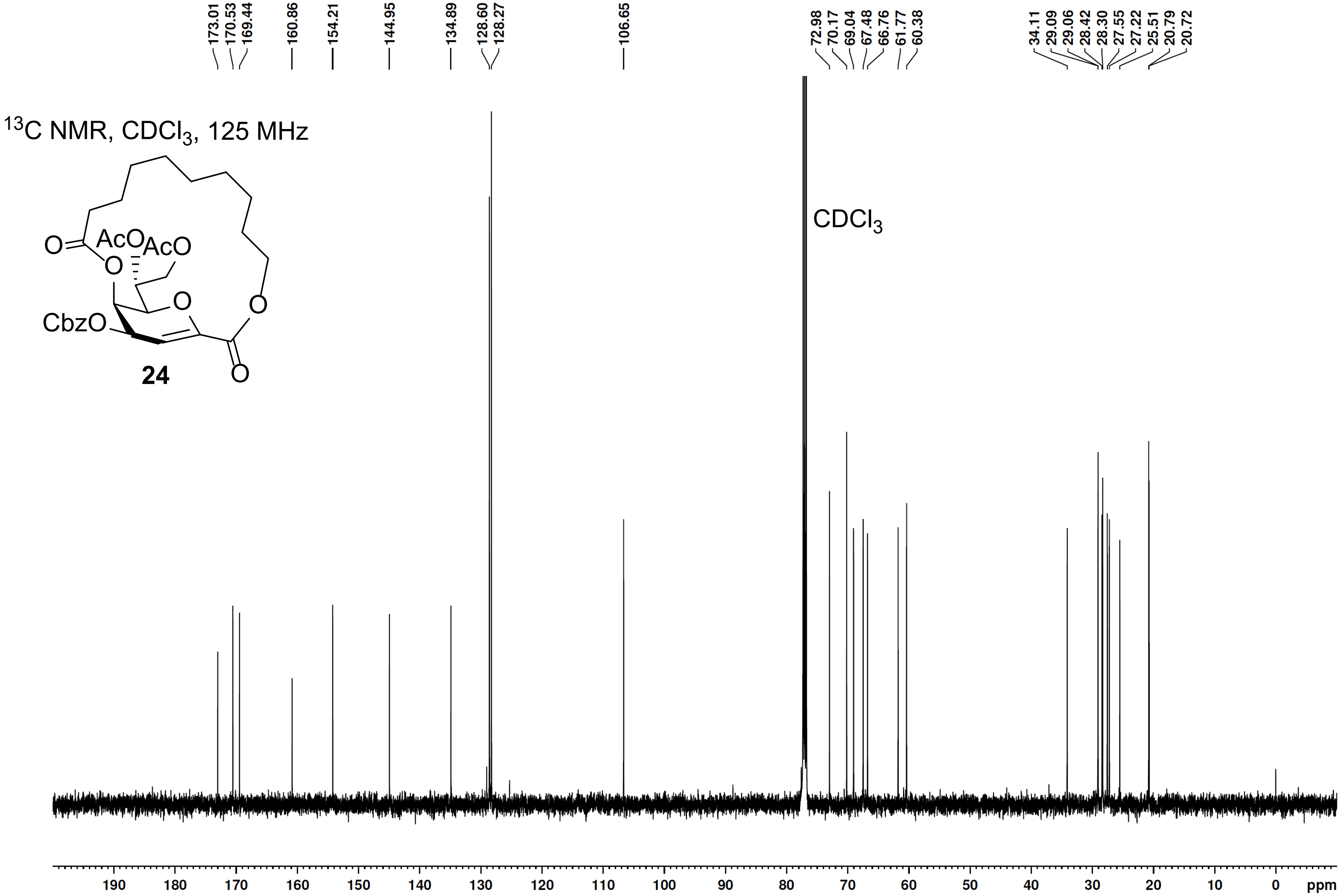
^{13}C NMR, $CDCl_3$, 125 MHz
AcO
AcO
CbzO
24
$CDCl_3$
173.01
170.53
169.44
160.86
154.21
144.95
134.89
128.60
128.27
106.65
72.98
70.17
69.04
67.48
66.76
61.77
60.38
34.11
29.09
29.06
28.42
28.30
27.55
27.22
25.51
20.79
20.72
190 180 170 160 150 140 130 120 110 100 90 80 70 60 50 40 30 20 10 0 ppm

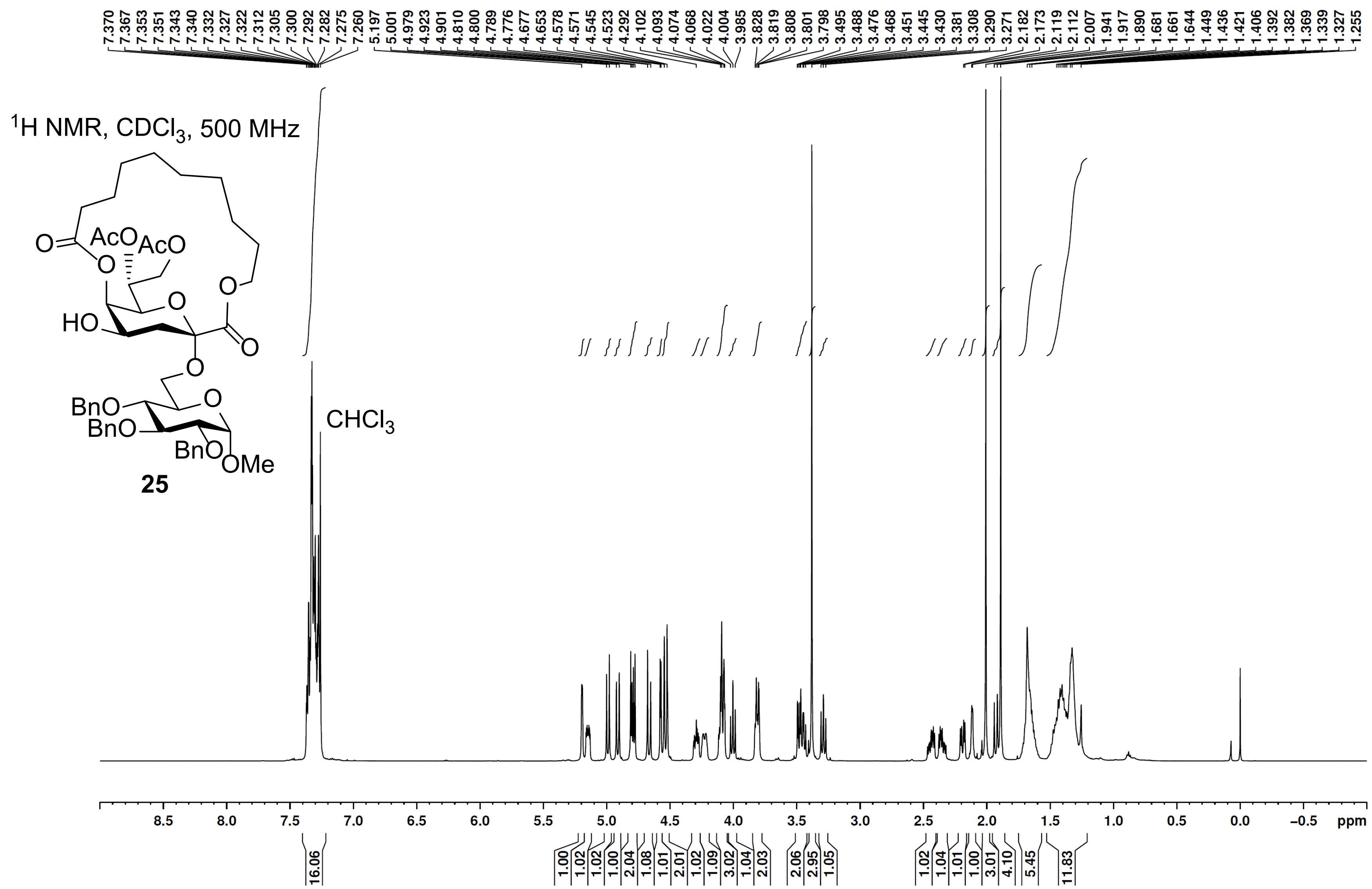
1H NMR, CDCl3, 500 MHz
CHCl3
25

^{13}C NMR, $CDCl_3$, 125 MHz

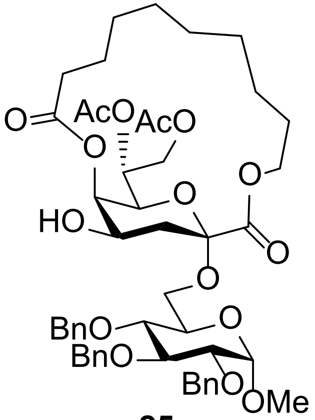

25

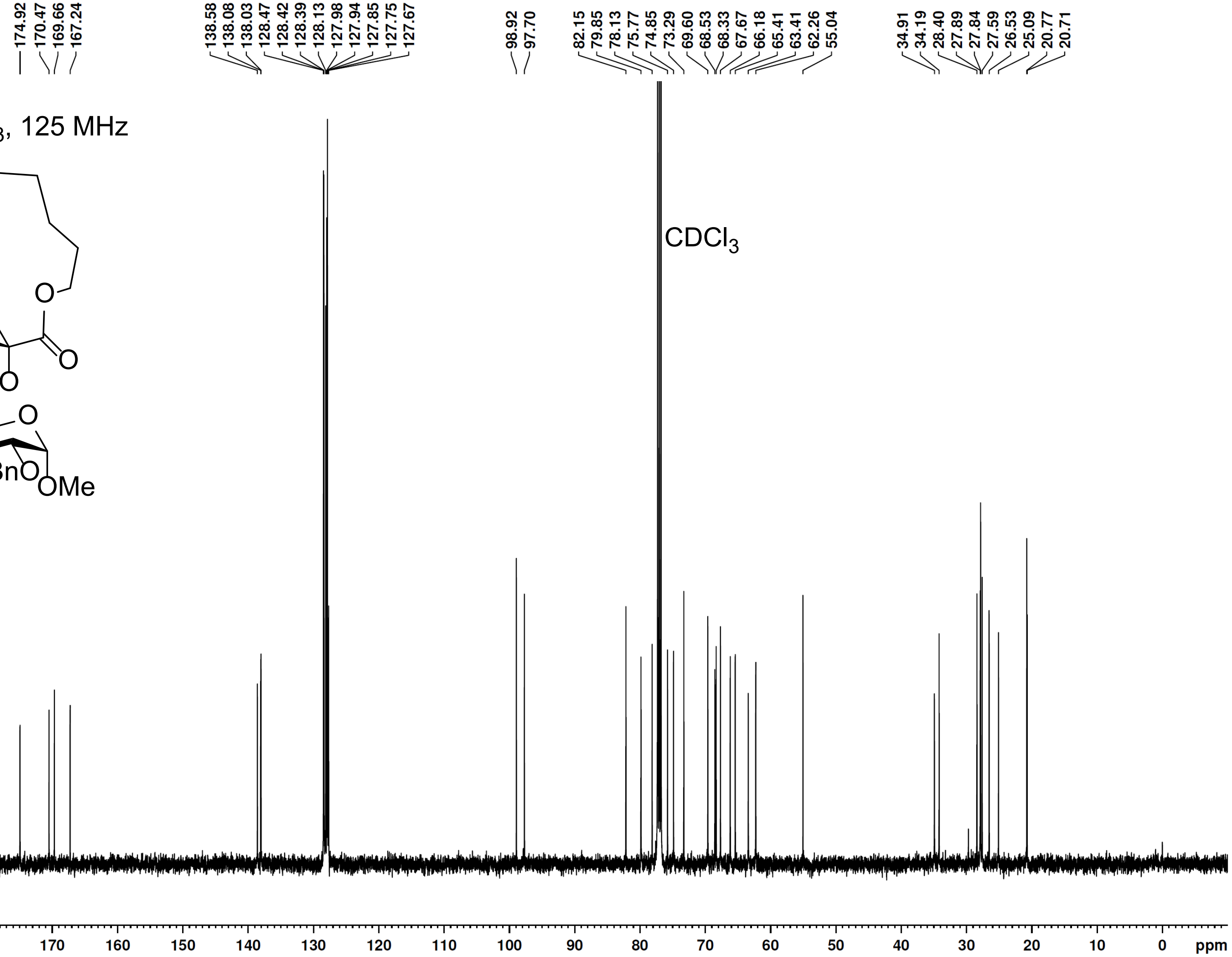

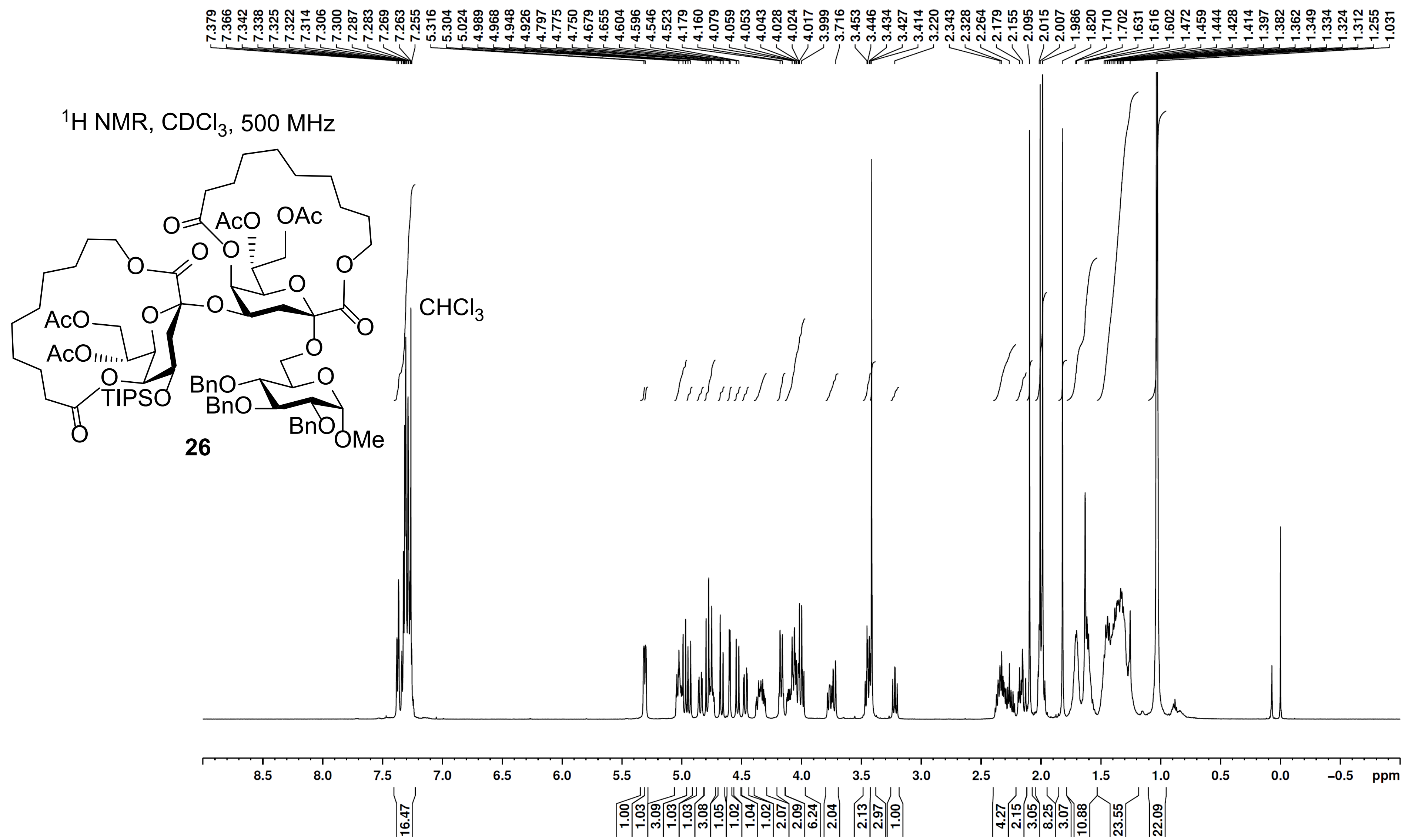

1H NMR, CDCl3, 500 MHz
CHCl3
AcO
OAc
TIPSO
BnO
OMe
26
7.379 7.366 7.342 7.338 7.325 7.322 7.314 7.306 7.300 7.287 7.283 7.269 7.263 7.255 5.316 5.304 5.024 4.989 4.968 4.948 4.926 4.797 4.775 4.750 4.679 4.655 4.604 4.596 4.546 4.523 4.179 4.160 4.079 4.059 4.053 4.043 4.028 4.024 4.017 3.999 3.716 3.453 3.446 3.434 3.427 3.414 3.220 2.343 2.328 2.264 2.179 2.155 2.095 2.015 2.007 1.986 1.820 1.710 1.702 1.631 1.616 1.602 1.472 1.459 1.444 1.428 1.414 1.397 1.382 1.362 1.349 1.334 1.324 1.312 1.255 1.031
8.5 8.0 7.5 7.0 6.5 6.0 5.5 5.0 4.5 4.0 3.5 3.0 2.5 2.0 1.5 1.0 0.5 0.0 –0.5 ppm
16.47 1.00 1.03 3.09 1.03 1.03 3.08 1.05 1.02 1.04 1.02 2.07 2.09 6.24 2.04 2.13 2.97 1.00 4.27 2.15 3.05 8.25 3.07 10.88 23.55 22.09

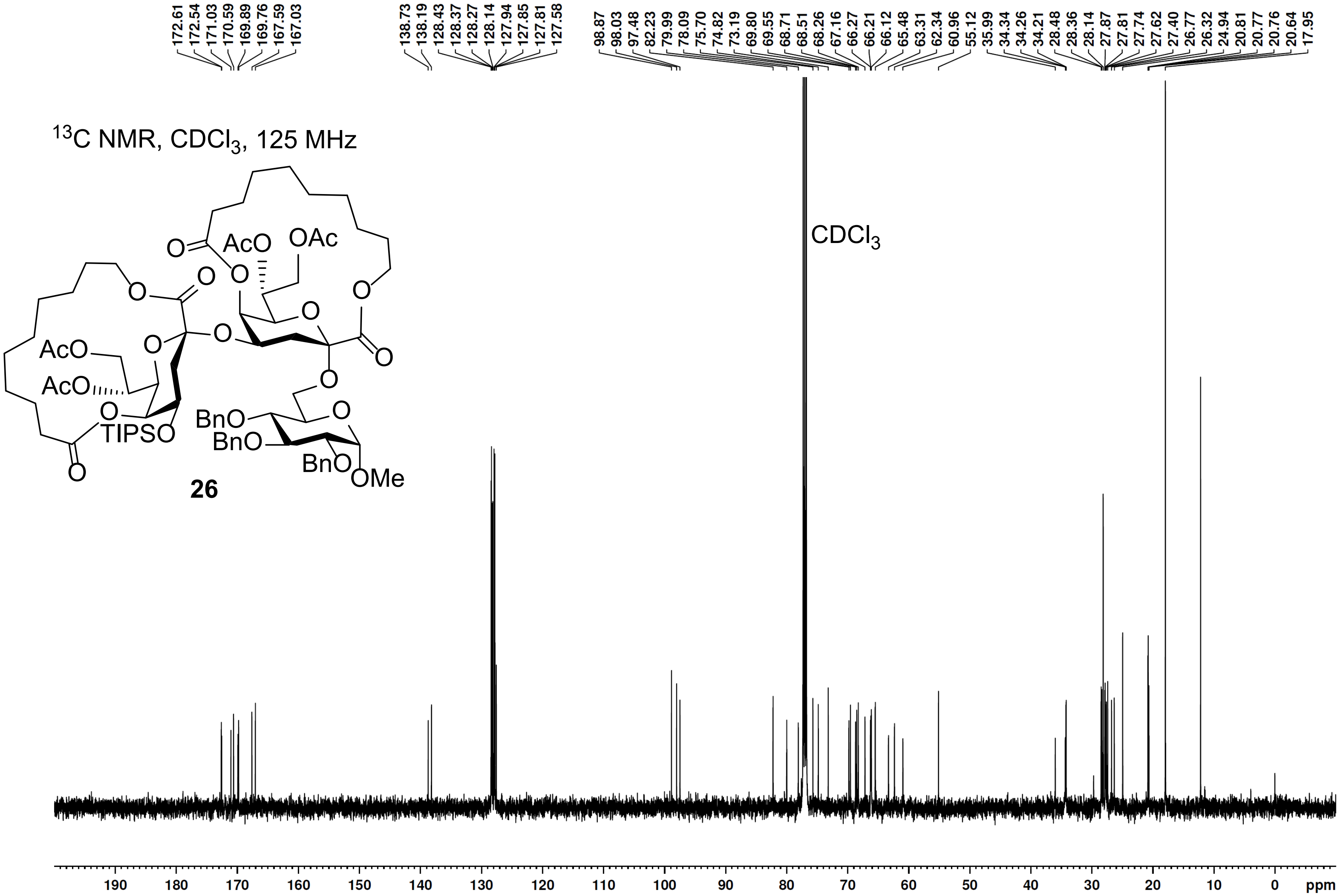
13C NMR, CDCl3, 125 MHz
26
CDCl3
172.61 172.54 171.03 170.59 169.89 169.76 167.59 167.03
138.73 138.19 128.43 128.37 128.27 128.14 127.94 127.85 127.81 127.58
98.87 98.03 97.48 82.23 79.99 78.09 75.70 74.82 73.19 69.80 69.55 68.71 68.51 68.26 67.16 66.27 66.21 66.12 65.48 63.31 62.34 60.96 55.12
35.99 34.34 34.26 34.21 28.48 28.36 28.14 27.87 27.81 27.74 27.62 27.40 26.77 26.32 24.94 20.81 20.77 20.76 20.64 17.95
190 180 170 160 150 140 130 120 110 100 90 80 70 60 50 40 30 20 10 0 ppm
